# Integration of Wallach's Rule into Intermolecular Charge Transfer: A Visual Strategy for Chiral Purification

**DOI:** 10.1002/advs.202403249

**Published:** 2024-07-16

**Authors:** Wei Wang, Jianye Gong, Jiaqiang Zhao, Hao Zhang, Wei Wen, Zujin Zhao, Yan Jie Li, Jianguo Wang, Cheng Zhi Huang, Peng Fei Gao

**Affiliations:** ^1^ Key Laboratory of Biomedical Analytics Chongqing Science and Technology Bureau College of Pharmaceutical Sciences Southwest University Chongqing 400715 China; ^2^ College of Chemistry and Chemical Engineering Inner Mongolia Key Laboratory of Fine Organic Synthesis Inner Mongolia University Hohhot 010021 China; ^3^ Chongqing Key Laboratory of Soft‐Matter Material Chemistry and Function Manufacturing School of Chemistry and Chemical Engineering Southwest University Chongqing 400715 China; ^4^ State Key Laboratory of Luminescent Materials and Devices Guangdong Provincial Key Laboratory of Luminescence from Molecular Aggregates South China University of Technology Guangzhou 510640 China

**Keywords:** intermolecular charge transfer, spectral analysis, visual chiral purification, Wallach's rule

## Abstract

Exploring the molecular packing and interaction between chiral molecules, no matter single enantiomer or racemates, is important for recognition and resolution of chiral drugs. However, sensitive and non‐destructive analysis methods are lacking. Herein, an intermolecular‐charge transfer (ICT) based spectroscopy is reported to reveal the differences in interaction between the achiral acceptor 1,2,4,5‐tetracyanobenzene (TCNB) and the chiral donors, including S, R, and racemic naproxen (S/R/rac‐NAP). In this process, S‐NAP+TCNB and R‐NAP+TCNB display a narrower band gap attributed to the newly formed ICT state. In contrast, the mixed rac‐NAP and TCNB exhibit almost no significant change due to the strong affinity between the stereoisomers according to the Wallach's rule. Thus, S/R‐NAP can be easily distinguished from rac‐NAP based on significantly different optical behavior. The single crystal analysis, infrared spectroscopy, fluorescence spectroscopy, and theoretical calculation of naproxen confirm the importance of carboxyl for this differentiation in molecular packing and interaction. In addition, the esterification derivatization of naproxen achieves the manipulation of the intermolecular interaction model of racemates from the absolute Wallach's rule to a coexisting form of Wallach's rule and ICT. Further, visualized chiral purification of naproxen by the simple cocrystallization method is achieved through the collaboration of ICT and Wallach's rule.

## Graphical abstract

Through the synergistic effect of intermolecular charge transfer (ICT) and Wallach's rule, a spectroscopy method is established to explore the different interactions between the achiral acceptor and the chiral donors. Further, visualized chiral purification of naproxen by a simple cocrystallization method is realized.

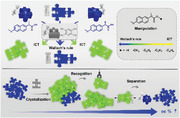

## 1 Introduction

Due to the high and continuously increasing proportion of chiral drugs in the medication therapy and newly developed drugs,^[^ ^1^ ^]^ chiral drug purification becomes more and more valuable in chiral chemistry and pharmacy. The most industrially valuable methods for chiral drug purification currently involve chiral separation,^[^ ^2^ ^]^ asymmetric synthesis,^[^ ^3^ ^]^ and crystallization methods.^[^ ^4^ ^]^ Crystallization methods for chiral drug purification are still widely used because of its simple operation, low cost, and environmental friendliness. The difference in affinity between single enantiomers or chiral isomers determines the forms and effectiveness of the separation. The most famous event is the simple separation of two single configuration ammonium tartrate salts by Pasteur using a microscope in 1848; while, chiral molecules typically follow the Wallach's rule, exhibiting stronger affinity and greater thermodynamic stability between racemates, which is often detrimental to chiral resolution based on crystallization methods.^[^ ^5^ ^]^ Thus far, through reasonable design, a series of crystallization methods‐based chiral resolution strategies has been extensively explored.^[^ ^6^ ^]^ However, the separation effect of enantioselective crystallization is always completely unknown before the evaluation by other techniques such as chiral high performance liquid chromatography (HPLC); and thus, the enantioselective crystallization is usually uncontrollable and incomplete. Therefore, a visual strategy to monitor the process of chiral purification and achieve the purpose of controllable prediction of chiral purification results is urgently needed but challenging.

The high consistency of various physical and chemical properties between the enantiomers of chiral molecules makes it difficult to achieve visual identification and resolution of the enantiomers, which has also been a long‐standing challenge. From the perspective of weak intermolecular interactions, combining the commonly used coordination interaction^[^ ^7^ ^]^ and some non‐covalent interactions, such as intermolecular hydrogen bonding,^[^ ^8^ ^]^ electrostatic interactions,^[^ ^9^ ^]^ to explore new optical indicator signals for visual analysis and purification of chiral molecules should be a reasonable approach. Among these intermolecular interactions, intermolecular charge transfer (ICT) can lead to a newly formed composite state with decreased energy gap,^[^ ^10^ ^]^ thereby, exhibiting significant changes in optical signals. This feature has enabled its varied applications in the construction of functional luminescent materials,^[^ ^11^ ^]^ near‐infrared imaging probes,^[^ ^12^ ^]^ photothermal agents,^[^ ^13^ ^]^ organic semiconductors,^[^ ^14^ ^]^ and analytical sensors.^[^ ^15^ ^]^ Moreover, it has been reported that the optical signals of ICT complexes formed between electron acceptors and different positional isomer donors exhibit significant differences.^[^ ^16^ ^]^ At the same time, Wallach's rule, which is the dominant regulatory form in racemic crystallization, has been cleverly integrated into the analysis of the affinity behavior, mechanism, and regulatory strategy of chiral molecules in recent years.^[^ ^17^ ^]^ On this basis, the synergistic ICT interaction and Wallach's rule to explore the intermolecular interaction between chiral molecules and the related visualized chiral purification strategy are highly anticipated.

**Scheme 1:**
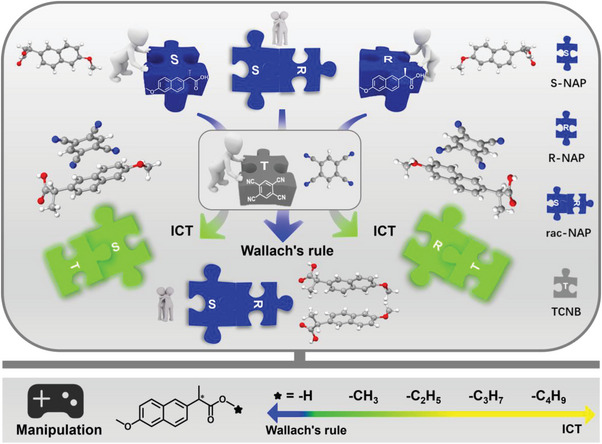
Differential steric interaction based on intermolecular charge transfer and Wallach's rule.

Herein, we integrate Wallach's rule into ICT to visually investigate the laws of intermolecular interactions of chiral drug molecules. Naproxen (S/R/rac‐NAP, donors) and 1,2,4,5‐tetracyanobenzene (TCNB, acceptor) are selected as the model donor and acceptor molecules. The single enantiomers, including S‐NAP and R‐NAP, show typical ICT feature when mixed and crystallized with TCNB; while, rac‐NAP exhibits no obvious changes in luminescence behavior when mixed with TCNB, indicating that Wallach's rule still exists and plays a major regulatory role. Single crystal analysis, infrared spectroscopy, and quantitative analysis of intermolecular interactions reveal that carboxyl in chiral side chain plays an important role in the Wallach's rule in this ICT system and leads to different luminescence behavior between single enantiomers or racemates. The characterization results of esterification products show that the intermolecular interaction behavior among the S configuration, R configuration, the racemate form of the esterification products, and TCNB tend to be similar (**Scheme** **1**). This effectively confirms the relevant mechanisms of the Wallach's rule in this ICT system. Further, by utilizing the significant differences in molecular stacking and luminescence behavior among racemates, single enantiomers, and TCNB, a simple and visualized chiral purification strategy is achieved. From the chiral HPLC results of the products purified by cocrystallization, visualized chiral purification can be achieved when the enantiomeric excess is higher than 40%, and the ee value can be easily enhanced to over 95% through several cycles of repeated crystallization and purification. This study provides an effective strategy for exploring the interactions, stacking forms, and luminescence behaviors of chiral molecules; while, demonstrating great application potential in the field of visualized chiral purification.

## 2 Results and Discussion

### 2.1 Photophysical Characterization of NAP+TCNB Complexes

First, naproxen (S configuration), one of the most consumed nonsteroidal anti‐inflammatory drugs, was selected as the charge transfer donor due to its electron‐rich naphthalene ring. Its isomer R‐NAP and the racemates rac‐NAP were also used for systematic analysis (Figure S1, Supporting Information). Second, TCNB, which contains four cyan and exhibits strong electron‐withdrawing capability, was selected as the charge transfer acceptor. Actually, both the number and the spatial distribution of the cyan in the acceptor were confirmed to be crucial for the efficient ICT interaction (Figures S2–S4, Supporting Information). In addition, we also explored the ICT interaction between nine naproxen structural analogues and TCNB, and all of these donor‐acceptor pairs exhibited obvious red shift in the fluorescence emission, suggesting that the electron‐rich naphthalene ring was important for the intense ICT interaction (Figure S5, Supporting Information).

**Figure 1:**
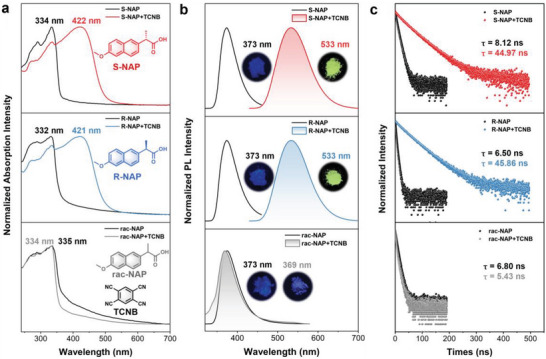
a) Molecular structures and normalized absorption spectra of S‐NAP, R‐NAP, and rac‐NAP before and after interaction with TCNB. b) Normalized PL spectra of S‐NAP, R‐NAP, and rac‐NAP before (*λ*_ex_ = 300 nm) and after (*λ*_ex_ = 410 nm; rac‐NAP+TCNB was measured under 300 nm irradiation) interaction with TCNB. Insert: the corresponding photographs of the fluorescence emission. c) Fluorescence decay curves of S‐NAP, R‐NAP, and rac‐NAP before and after interaction with TCNB.

The self‐assembled single crystals of different ICT complexes (S‐NAP+TCNB, R‐NAP+TCNB, and rac‐NAP+TCNB) were prepared by gas phase diffusion method. There was a newly formed peak (421 or 422 nm) in the solid UV– visible absorption spectra of S‐NAP+TCNB and R‐NAP+TCNB compared with the initial state of S‐NAP and R‐NAP (**Figure** **1**a; Figures S6 and S7, Supporting Information). This suggested the formation of the ICT state, leading to an obvious red shift and resulting in yellow crystals; while, the crystals of rac‐NAP+TCNB displayed no significant change in absorption and color. Similarly, upon UV irradiation, the fluorescence of both S‐NAP+TCNB and R‐NAP+TCNB was yellowish green (533 nm); while, the rac‐NAP+TCNB showed no significant change in fluorescence emission compared with the initial state (Figure 1b; Figures S8 and S9, Supporting Information).

The fluorescence decay curve indicated that the fluorescence lifetime of the S‐NAP+TCNB and R‐NAP+TCNB cocrystals was 44.34 and 45.49 ns, respectively, which was obviously longer than those of the enantiomers (Figure 1c). The strong intermolecular interaction of the charge transfer cocrystals suppressed the rotational and vibrational non‐relaxation of the ground state; thus, extending the lifetime of the cocrystals.^[^ ^18^ ^]^ However, the fluorescence lifetime of rac‐NAP+TCNB was 5.43 ns, which was even shorter than the crystal of rac‐NAP or TCNB. Therefore, there seemed to be no ICT interaction occurring between the rac‐NAP and TCNB, which was consistent with the absorption and fluorescence characterization results.

### 2.2 Differences in Interactions Between Chiral Enantiomers and Racemates

**Figure 2:**
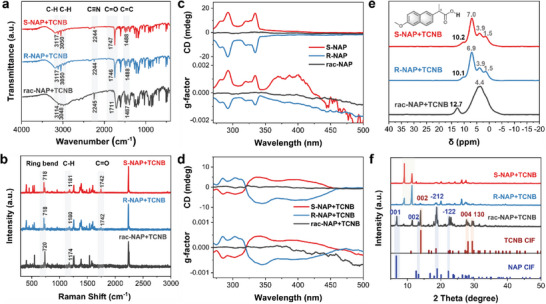
a) FTIR spectra and b) Raman spectra of the S‐NAP+TCNB, R‐NAP+TCNB, and rac‐NAP+TCNB. c) CD spectra of S‐NAP, R‐NAP, and rac‐NAP. d) CD spectra of S‐NAP+TCNB, R‐NAP+TCNB, and rac‐NAP+TCNB. e) Solid‐state ^1^H NMR spectra of the S‐NAP+TCNB, R‐NAP+TCNB, and rac‐NAP+TCNB. f) PXRD patterns of the S‐NAP+TCNB, R‐NAP+TCNB, and rac‐NAP+TCNB, and the simulated XRD signal from cif file.

In order to understand the interaction and luminescence differences in these cocrystals, further spectroscopic and structural characterization of the crystals was performed. First, the Fourier transform infrared (FTIR) and Raman spectra of the cocrystals were determined and used to investigate the non‐covalent interaction between the NAP+TCNB complexes. As shown in **Figure** **2**a; Figure S10 and Table S1, Supporting Information, the FTIR spectral bands of TCNB showed a slight shift in S‐NAP+TCNB and R‐NAP+TCNB, (C─H str: from 3114 to 3117 cm^−1^; C─H str: from 3048 to 3050 cm^−1^; C≡N str: from 2245 to 2244 cm^−1^; C═C str: from 1468 to 1488 cm^−1^). The overall red shift of wavenumber indicated an increase in the electron cloud density of the benzene ring in the TCNB molecule. Meanwhile, the corresponding peaks of S/R‐NAP in the cocrystals were shifted (C═O str: from 1728/1729 cm^−1^ to 1746/1747 cm^−1^), which also indicated the existence of ICT interactions between S/R‐NAP with TCNB; while, the wavenumber of C═O in rac‐NAP was 1710/1711 cm^−1^, which was quite different from that in S‐NAP or R‐NAP and suggested the different interacting state of the carboxyl in chiral enantiomers and racemes. Moreover, in the rac‐NAP+TCNB, there were no significant changes compared to rac‐NAP, except for a broader hydroxyl peak near 3100 cm^−1^ that predicted stronger intermolecular hydrogen bonding of racemates. On the contrary, the significant shift in the S/R‐NAP+TCNB suggested the carboxyl should be a key interaction site and crucial for the change of the physical properties. From the Raman spectra, the ring bend of TCNB in the S/R‐NAP+TCNB cocrystals was blue‐shifted from the initial 721 to 718 cm^−1^, indicating an increase in the electron cloud density. In contrast, the peak of C─H of the donor S/R‐NAP was significantly red‐shifted from 1174 to 1180 cm^−1^. A new peak of C═O appeared at 1742 cm^−1^ in the cocrystals,^[^ ^19^ ^]^ which might be attributed to the important role of carboxyl in the ICT interaction (Figure 2b; Figure S11 and Table S2, Supporting Information); while, there was no visible peak shift in the complexes formed by rac‐NAP+ TCNB, showing that there should be no significant interaction between rac‐NAP and TCNB.

To document the retention of chirality after donor–acceptor interactions, the CD signals were tested. The S/R‐NAP+TCNB cocrystals showed apparent Cotton effects and a significant red shift relative to the initial state, which was consistent with the absorption spectra and indicated the formation of ICT state (Figure 2c,d). The absence of Cotton signal in the wavelength range detected for rac‐NAP+TCNB suggested the maintenance of the racemic structure. This result was also an effective evidence of the stronger affinity between the R and S isomers than the chiral enantiomers. The fast fading fluorescence emission of the locally formed ICT complex of rac‐NAP (a small amount of S‐NAP/S‐NAP) and TCNB after heating and annealing further demonstrated the stronger affinity between R‐NAP and S‐NAP than that between the locally formed S/R‐NAP+TCNB (Figure S12, Supporting Information). Attenuation of fluorescence emission of the locally formed ICT complex was also observed in the aggregation and sedimentation of the mixed rac‐NAP and TCNB in the solution. Nonetheless, the maintained fluorescence emission of S/R‐NAP+TCNB ICT complexes revealed the stronger affinity of these pairs than that of the interior of S/R‐NAP and the interior of TCNB, which was an important foundation for the effective formation of ICT composites.

Meanwhile, the intermolecular interactions of the cocrystals were further investigated by solid‐state ^1^H NMR spectra. As shown in Figure 2e, the results provided evidence that enantiomeric selectivity might stem from differences in O···H interactions. The chemical shift of protons in carboxyl groups in both S‐NAP and R‐NAP was ≈11.5 ppm; while, in rac‐NAP, these peaks moved toward the lower field of 12.8 ppm, supporting stronger O···H interactions between enantiomers (Figure S13, Supporting Information). It was worth mentioning that the chemical shift of proton in the carboxyl group of S/R‐NAP+TCNB moved from ≈11.5 ppm to ≈10.1 ppm due to the formation of a new ICT interaction between donor and acceptor, altering the O···H interactions on naproxen. As rac‐NAP does not interact with TCNB, the chemical shift of the O···H was still 12.7 ppm. Further, we compared their stacking patterns in the powder X‐ray diffraction (PXRD) pattern and found that rac‐NAP+TCNB exhibited the superposition of the peaks of these two components. There were newly formed diffraction peaks for S/R‐NAP+TCNB, suggesting that the initial crystal structure of a single component was changed during donor–acceptor recognition process and new crystalline phases were formed in the ICT composites (Figure 2f; Figure S14, Supporting Information).

Subsequently, the thermal stability of the cocrystals and single component NAP crystals was also determined and analyzed. The thermogravimetric analysis (TGA) curves showed that the sublimation temperature of cocrystals was different from that of single component crystals, indicating that a new lattice might be formed,^[^ ^20^ ^]^ and the intermolecular interaction in cocrystals was stronger than that of single component NAP crystals (Figure S15a, Supporting Information). Differential scanning calorimetry (DSC) diagram demonstrated that the melting points of S/R‐NAP+TCNB were also different from the melting points of the individual S/R‐NAP or TCNB crystals, implying that the cocrystals formed a new lattice. Higher melting point indicated that the ICT cocrystal structure was more stable, attributed to the stronger interaction between molecules.^[^ ^21^ ^]^ In contrast, the melting point of rac‐NAP+TCNB was even lower than that of the naproxen (Figure S15b, Supporting Information). Up to now, almost all the data clearly indicated the different interaction and the corresponding photophysical properties between the S/R‐NAP+TCNB and rac‐NAP+TCNB.

### 2.3 Synergy Mechanism of ICT and Wallach's Rule

**Figure 3:**
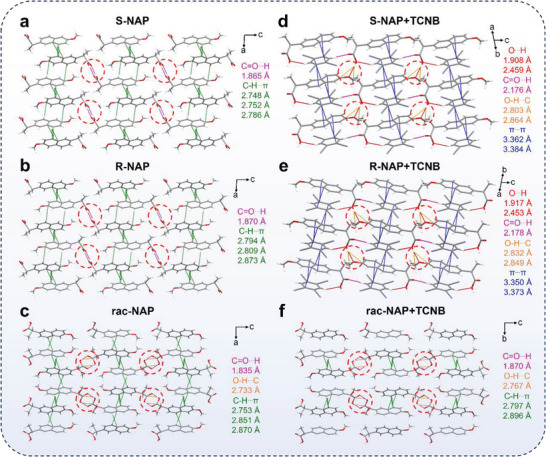
Molecular packing and intermolecular interactions of S‐NAP, R‐NAP, and rac‐NAP a–c) before and d–f) after interaction with TCNB.

To gain more insights into the mechanism of the synergism of ICT and Wallach's rule, the molecular conformation of single crystals for chiral isomers, racemates, and ICT composites was studied in detail. The S‐NAP and R‐NAP were monoclinic crystal systems; while S/R‐NAP+TCNB were triclinic crystal systems. Besides, rac‐NAP+TCNB and rac‐NAP were both rhombic crystal systems, indicating the remarkable different molecular packing modes in this system (Tables S3 and S4, Supporting Information). As shown in **Figure** **3**a,b; Figure S16, Supporting Information in S‐NAP, there were C─H···π interactions with distances ranging from 2.748 to 2.786 Å and C═O···H interaction with a distance of 1.865 Å. In comparison to S‐NAP, R‐NAP had the same types of intermolecular interactions, but only the distances of the interactions were slightly different, indicating the same molecular packing mode of S‐NAP and R‐NAP. In S/R‐NAP+TCNB, the donor and acceptor molecules were arranged alternately face to face owing to the ICT interaction. Multiple types of intermolecular interactions were formed; however, the C═O···H interaction between carboxyl groups disappeared. Although O···H interaction between carboxyl and methoxy groups and C═O···H interaction between carboxyl groups and TCNB were observed, they were passively formed and had limited effect on the overall molecular packing (Figure 3d,e). Therefore, the stronger ICT interactions between donor and accepter determined the molecular packing in S/R‐NAP+TCNB.

**Figure 4:**
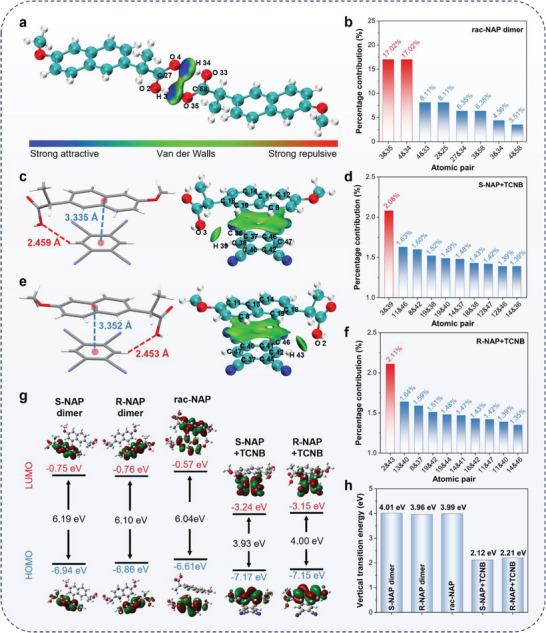
a) Visualized isosurfaces of the IGM analysis (green isosurface represents van der Waals interactions) of rac‐NAP+TCNB. b) The contribution of atomic pairs with the largest percentage to intermolecular interactions of rac‐NAP+TCNB. The intermolecular distance (left) and visualized isosurfaces of the IGM analysis and pairs of atoms interacting between molecules (right) of c) S‐NAP+TCNB and e) R‐NAP+TCNB. The contribution of ten atomic pairs with the largest percentage to intermolecular interactions of d) S‐NAP+TCNB and f) R‐NAP+TCNB. g) Calculated HOMOs and LUMOs of the single crystals. h) Calculated the first singlet excited state (S_1_) based on TD‐DFT.

To further illustrate the change in mode of action induced by structural isomerization, the stabilization energies of S/R‐NAP isomers and S/R‐NAP+TCNB ICT composites were calculated (Table S5, Supporting Information). Adjacent dimers were extracted for detailed analysis. The calculated stabilization energies of S‐NAP dimer and R‐NAP dimer compared to the enantiomer were −161.174 and −173.827 eV, respectively. The stabilization energies of S‐NAP+TCNB and R‐NAP+TCNB were −418.593 and −422.920 eV, respectively, indicating that S/R‐NAP+TCNB were more stable than individual isomers. These results explained the possible formation reasons of ICT cocrystals. For rac‐NAP, S‐NAP and R‐NAP were preferentially bound due to the Wallach's rule. As shown in Figure 3c, the strong intermolecular interactions between carboxyl groups of S‐NAP and R‐NAP were formed, including double C═O···H interactions (1.835 Å) and double O─H···C interactions (2.733 Å). Meanwhile, multiple C─H···π interactions (2.753–2.870 Å) were formed in intermolecular in rac‐NAP. When TCNB was added, the types of intermolecular interactions of rac‐NAP+TCNB had no significant changes, and S‐NAP and R‐NAP were still tightly bound according to the Wallach's rule (Figure 3f). These results indicated that the influence of Wallach's rule in molecular packing of rac‐NAP was larger than that of ICT interactions. Notably, the double C═O···H interaction was the strongest interaction type both in rac‐NAP and rac‐NAP+TCNB, which might be the critical factor to Wallach's rule in this system. To confirm this hypothesis, the independent gradient model (IGM) analysis^[^ ^22^ ^]^ was conducted to visualize and analyze the weak interactions by using the Multiwfn program^[^ ^23^ ^]^ and the visual molecular dynamics (VMD) program.^[^ ^24^ ^]^ A pair of S‐NAP and R‐NAP from rac‐NAP was selected as the research object. As seen in **Figure** **4**a, the obvious blue isosurfaces were observed between carboxyl groups of S‐NAP and R‐NAP, indicating the formation of double C═O···H interaction. The contribution of atomic pairs to the total interaction was further investigated. The percentage contributions of 3 & 35 and 4 & 34 atomic pairs that were matched with double C═O···H interaction were both 17.02%, which was the highest proportion in all atomic pairs (Figure 4b). These results not only further illustrated the formation of double C═O···H interaction but also revealed the key action site of the Wallach's rule.

In addition, compared to isomers and racemates, ICT composites showed obviously different photophysical properties. In order to elucidate the structure–activity relationships, a pair of ICT composites, one from S‐NAP+TCNB and the other from R‐NAP+TCNB, was extracted for detailed analysis. As illustrated by Figure 4c–f, the intermolecular interactions of ICT composites were mainly distributed in two sites. One was the π–π interaction between the benzene ring of TCNB and the naphthalene ring of S/R‐NAP, with a distance of ≈3.335 Å for S‐NAP+TCNB and 3.352 Å for R‐NAP+TCNB. Another was the O···H interaction between the carboxyl in the chiral side chain of S/R‐NAP and the hydrogen in the benzene ring of TCNB (2.459 Å for S‐NAP+TCNB and 2.453 Å for R‐NAP+TCNB). Remarkably, the isosurface distributions of π–π interactions were significantly larger than that of O···H, indicating that π–π interactions were major components of ICT interactions. Moreover, the contribution of atomic pairs to the total intermolecular interaction showed that 3&39 of S‐NAP+TCNB (2.08%) and 2&43 of R‐NAP+TCNB (2.11%) were both ranked first among all atomic pairs (Table S6, Supporting Information). Therefore, π–π interactions and O···H interactions together affected the degree of ICT interaction and influenced the photophysical properties of ICT composites. There was no significant difference between S‐NAP+TCNB and R‐NAP+TCNB, and even the isosurfaces and the distribution of δg^inter^ (the descriptor for defining intermolecular interaction regions) showed no apparent distinction (Figure S17, Supporting Information). These results explained that S‐NAP+TCNB and R‐NAP+TCNB had no distinguishable photophysical properties.

To gain deep insight into the red‐shifted optical properties of ICT composites, theoretical calculations were performed based on density functional theory (DFT). Δ*E*_g_ is the energy gap of the highest occupied molecular orbital (HOMO) and lowest unoccupied molecular orbital (LUMO). As shown in Figure 4g; Table S7, Supporting Information, the Δ*E*_g_ of S‐NAP dimer, R‐NAP dimer, and rac‐NAP were 6.19, 6.10, and 6.04 eV, respectively. However, for S‐NAP+TCNB and R‐NAP+TCNB, the Δ*E*_g_ were only 3.93 and 4.00 eV. These results suggested that strong ICT interaction effectively reduced the Δ*E*_g_ and resulted in the red shift of the absorption of S/R‐NAP+TCNB. In addition, the property of the first singlet excited state (S_1_) was investigated based on time‐dependent density functional theory (TD‐DFT). The S_1_ energy levels of S‐NAP dimer, R‐NAP dimer, and rac‐NAP were 4.01, 3.96, and 3.99 eV, respectively. By contrast, S‐NAP+TCNB and R‐NAP+TCNB showed 2.12 and 2.21 eV of S1 energy levels, which was consistent with the experimental results (Figure 4h). These results explained the red shift of the fluorescence emission of S/R‐NAP+TCNB. Simultaneously, the calculated natural transition orbitals (NTOs) showed that the electronic configurations of S_1_ state in S‐NAP+TCNB and R‐NAP+TCNB were dominated by intermolecular π→π* transition. In contrast, other dimers possessed an intramolecular π→π* transition, which further demonstrated the ICT interactions in S/R‐NAP+TCNB (Figure S18, Supporting Information).

### 2.4 Manipulation of ICT and Wallach's Rule

**Figure 5:**
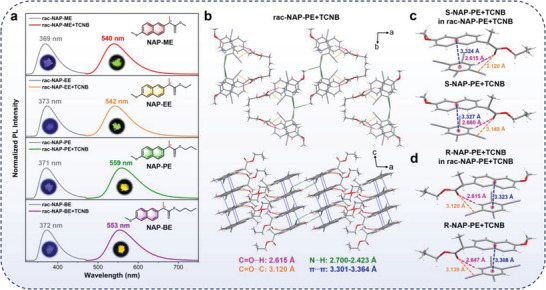
a) Molecular structures of esterification derivative products, normalized PL spectra, and photographs before (*λ*_ex_ = 300 nm) and after (*λ*_ex_ = 400 nm) interaction with TCNB. Molecular packing and intermolecular interactions of b) rac‐NAP‐PE+TCNB, c) S‐NAP‐PE+TCNB in rac‐NAP‐PE+TCNB and S‐NAP‐PE+TCNB, and d) R‐NAP‐PE+TCNB in rac‐NAP‐PE+TCNB and R‐NAP‐PE+TCNB.

According to the analysis results from the single crystal data and the calculation results, the carboxyl on the chiral side chains, especially the hydrogen atom of carboxyl, played a crucial role in manipulating ICT and Wallach's rule. To verify this hypothesis, we designed and performed the modification experiment of carboxyl. First, we successfully prepared four pairs of esterified derivatives of S/R‐NAP, namely S/R naproxen methyl ester (S/R‐NAP‐ME), S/R naproxen ethyl ester (S/R‐NAP‐EE), S/R naproxen propyl ester (S/R‐NAP‐PE), and S/R naproxen butyl ester (S/R‐NAP‐BE). In these esterified derivatives, the carboxyl was transformed into ester group (**Figure** **5**a; Figures S19–S46 and Schemes S1–S4, Supporting Information). As the alkyl chain gradually increased, its ability to form hydrogen bonds gradually decreased. Interestingly, after interacting with the acceptor TCNB, the fluorescence emission peaks of these four esterified derivatives‐TCNB complexes showed obvious red shift, exhibiting classical ICT effect (Figures S47–S50 and Table S8, Supporting Information). The cocrystals of S/R‐NAP‐PE+TCNB, and rac‐NAP‐PE+TCNB were successfully obtained (Table S9, Supporting Information). As illustrated by Figure 5b, compared to the double C═O···H interaction of rac‐NAP+TCNB, no obvious intermolecular interactions were observed between ester groups in rac‐NAP‐PE+TCNB. The ICT composites were formed, and the molecular packing of rac‐NAP‐PE+TCNB altered to be comparable to S/R‐NAP‐PE+TCNB. Specifically, the types of intermolecular interactions for S/R‐NAP‐PE+TCNB dimers in rac‐NAP‐PE+TCNB were the same as those for S/R‐NAP‐PE+TCNB except for a slight difference in the interaction distance (Figure 5c,d; Figure S51, Supporting Information). The related stabilization energies of these ICT composites were also very similar (Table S10, Supporting Information). Therefore, there were no obvious differences in fluorescence emission of rac‐NAP‐PE+TCNB compared to that of S/R‐NAP‐PE+TCNB. Collectively, for all these four esterified derivatives, their racemic forms no longer followed Wallach's rule but exhibited obvious ICT effect. These results verified that esterification derivatization of carboxyl could effectively weaken the C═O···H interaction between the enantiomers, realizing the manipulation of ICT and Wallach's rule in this system.

### 2.5 Visualized Chiral Purification Based on the Integration of ICT and Wallach's Rule

**Scheme 2:**
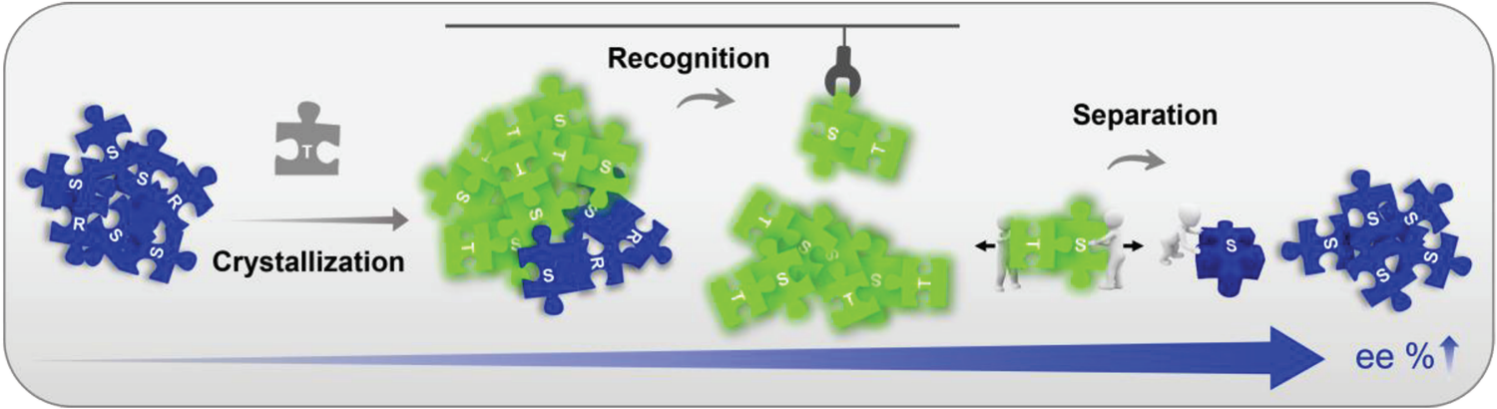
Visualized chiral purification based on the integration of Wallach's rule and ICT.

Chiral purification based on enantiomer recognition crystallization shows high selectivity, and has attracted much attention due to its economical and convenient. Considering that TCNB has a special interaction with naproxen enantiomers, we attempted to combine ICT and Wallach's rule to realize visualized chiral purification of naproxen (**Scheme** **2**). Therefore, the cocrystals of TCNB and naproxen with different ee values were grown according to the stoichiometric ratio. Due to the unique ICT interaction between naproxen enantiomers and TCNB, the absorption and fluorescence emission of ICT cocrystals were red‐shifted, which facilitated the visualization of cocrystals selecting the excess single enantiomers. Moreover, after crystallization, the purification effect could be predicted by the absorption and fluorescence emission of the cocrystals. Then, the cocrystals selected by visual recognition were separated by column chromatography to achieve the separation of TCNB and naproxen enantiomers, and the naproxen after chiral purification could be obtained. Finally, the purity of NAP enantiomers after purification was determined by chiral HPLC. As shown in **Table** **1**; Figures S52–S55, Supporting Information, chiral HPLC analysis data showed this strategy could effectively improve the chiral purity of naproxen with different ee values, regardless of S‐NAP excess or R‐NAP excess. It is noteworthy that chiral purification could be achieved when the enantiomeric excess of naproxen was 40%. Moreover, when the ee value was between 40% and 80%, an improvement of ≈15% could always be achieved. When the enantiomer excess was 90%, the enantiomeric excess could also be elevated to 95%/96%. It could be expected that if purified by repeating this procedure, higher levels of chiral purity might be obtained. To sum up, this strategy could realize the visualized chiral purification of naproxen through the economical and convenient crystallization method. In addition, the operation of separation and purification was simple and effective.

**Table 1:** Results of visualized chiral purification of naproxen with different ee values.

Entry^a)^	ee [%]^b)^	ee [%]^c)^	ee [%]^c)^	ee [%]^c)^	ee [%]^d)^
1	90.1	96.0	94.3	96.6	95.6 ± 1.2
2	81.2	90.5	92.9	94.7	92.7 ± 2.1
3	59.8	76.4	87.4	70.4	78.1 ± 8.6
4	39.5	58.8	62.1	54.2	58.4 ± 4.0
5	20.6	–	–	–	–
6	0.9	–	–	–	–
7	−20.1	–	–	–	–
8	−39.6	−52.3	−57.0	−48.9	−52.7 ± 4.1
9	−59.9	−76.2	−73.9	−67.7	−72.6 ± 4.4
10	−80.1	−94.0	−93.4	−94.6	−94.0 ± 0.6
11	−90.1	−94.8	−94.9	−96.4	−95.4 ± 0.9

^a)^ The general procedure for the visualized chiral purification: TCNB and NAP with different ee values were mixed in acetonitrile solvent at stoichiometric ratio; and then, let to stand for a certain time. The yellowish green partial fluorescent crystals were selected, NAP was obtained by column chromatography, and the chiral purity was determined by chiral HPLC;

^b)^ ee values of the sample before visualized chiral purification (definition: positive values represent excess S‐NAP and negative values represent excess R‐NAP);

^c)^ ee values of three groups of parallel samples after visualized chiral purification;

^d)^ The mean values and standard deviations (SDs) of ee values of three groups of parallel samples after visualized chiral purification. Data are presented as mean values ± SDs.

## 3 Conclusion

In summary, we designed and constructed an ICT‐based fluorescence method for investigating the different intermolecular affinities and luminescent behavior using R, S, rac‐NAP, and TCNB as model molecules. The differentiation in the conformation in the S/R‐NAP+TCNB and individual rac‐NAP confirmed from the single crystal structure analysis was the structural foundation of the ICT composites with decreased energy gap. The theoretical calculation, together with the FTIR spectroscopy, esterification derivatization, revealed the crucial role of carboxyl in the Wallach's rule and ICT interaction. The carboxyl was also the effective site to achieve the manipulation of the interaction form from Wallach's rule to a composite form of Wallach's rule and ICT. According to the intermolecular stacking priority, a simple but effective crystallization purification of naproxen was realized by using the red shift of absorption and fluorescence emission as resolution signals. This provided a feasible and sensitive analytical method in the narrow space between the interaction strengths of chiral isomers and single enantiomers; and by introducing the effective chiral recognition elements, the integration of Wallach's rule and ICT interaction would be of great value for the future.

## Conflict of Interest

The authors declare no conflict of interest.

## Supporting information

Supporting Information

## Data Availability

The data that support the findings of this study are available from the corresponding author upon reasonable request.
