# Supplementary material for: Integration of Wallach's Rule into Intermolecular Charge Transfer: A Visual Strategy for Chiral Purification
Source: Adv Sci (Weinh). 2024 Jul 16;11(35):2403249. doi: 10.1002/advs.202403249 (PMC11425254; doi:10.1002/advs.202403249)
Supplement: Supplementary file 1 — Supporting Information [file ADVS-11-2403249-s001.docx]

Supporting Information

Integration of Wallach’s Rule into Intermolecular Charge Transfer: A Visual Strategy for Chiral Purification

Wei Wang, Jianye Gong, Jiaqiang Zhao, Hao Zhang, Wei Wen, Zujin Zhao, Yan Jie Li,* Jianguo Wang,* Cheng Zhi Huang, Peng Fei Gao*

**Table of Contents**

[**Experimental Procedures** 2](#_Toc167303910)

[1.1 Main Materials 2](#_Toc167303911)

[1.2 Instruments 2](#_Toc167303912)

[1.3 Density Functional Theory Calculations 2](#_Toc167303913)

[**Synthesis Procedure** 3](#_Toc167303914)

[2.1 Synthesis and Characterization 3](#_Toc167303915)

[2.2 Crystal Growth and Sample Preparation 6](#_Toc167303916)

[**Supplementary Figures and Tables** 7](#_Toc167303917)

**Experimental Procedures**

## 1.1 Main Materials

S-naproxen (S-NAP), R-naproxen (R-NAP), rac-naproxen (rac-NAP), 1,2,4,5-tetracyanobenzene (TCNB), 1,2-dicyanobenzene (1,2-DCB), 1,3-dicyanobenzene (1,3-DCB), 1,3,5-benzenetricarbonitrile (BTC), 7,7,8,8-tetracyanoquinodimethane (TCNQ), n-propanol and acetonitrile (CH_3_CN, GC) were purchased from Aladdin Bio-Chem Technology Co., Ltd (Shanghai, China). Methanol, ethanol absolute, n-butanol, petroleum ether, dichloromethane, concentrated sulfuric acid and chloroform-d (CDCl_3_) were purchased from Chuandong Chemical Reagent Co., Ltd (Chongqing, China). S-NAP, rac-NAP and TCNB were purified by silica gel column chromatography and recrystallization. The chiral separation of R-NAP has been completed by HPLC using Chiralpak IH.

## 1.2 Instruments

Ultraviolet-visible absorption (UV) spectra were obtained by the UH4150 UV-Vis-NIR spectrophotometer combined with the diffuse reflection mode of the integrating sphere (Hitachi, Japan). Steady-state photoluminescence (PL) spectra were recorded on a F-7100 fluorescence spectrophotometer (Hitachi, Japan). PL lifetime was measured by FLS1000 fluorescence spectrometer (Edinburgh Instruments, UK). Fourier transform infrared (FTIR) spectra were measured on the IRTracer-100 spectrophotometer (Shimadzu, Japan). Raman spectra were collected on the LabRAM HR800 spectrometer with a 633 nm laser excitation source (Horiba Jobin-Yvon, France). Powder X-ray diffraction (PXRD) measurements were made by a X'Pert^3^ Powder10300 (PANalytical, Holland). Thermogravimetric analysis (TGA) curve was measured on a TG-DSC 3+ instrument under a nitrogen atmosphere, and the heating rate was 10 ^o^C·min^-1^ (Mettler Toledo, Switzerland). Differential scanning calorimetry (DSC) diagram was obtained on a DSC25 with a heating rate of 10 ^o^C·min^-1^ (TA, USA). The circular dichroism (CD) spectra were measured by a Chirascan-plus ACD instrument (Applied Photophysics Ltd, Britain). ^1^H and ^13^C NMR spectra were recorded on a Bruker Avance III instrument and using Chloroform-d (CDCl_3_) as solvent. High resolution mass spectra (HRMS) were recorded on a Bruker impact II10200 mass spectrometer. Enantiomer ratios were determined by HPLC (Chiralpak AS-H columns were purchased from Daicel Chemical Industries, LTD). All single-crystal X-ray diffraction data were collected on a XtaLAB Synergy, Dualflex, HyPix diffractometer with Cu Kα radiation (*λ*=1.54184 Å). With Olex2, all the structures were solved using the SHELXS structure solution program using the intrinsic phasing method, and all the structures were refined with the SHELXL refinement package using least-squares minimization. Photographs were taken by an iPhone 13 camera.

## 1.3 Density Functional Theory Calculations

All calculations were performed within Gaussian 16 program.^[1]^ The HOMOs and LUMOs orbital distribution of the monomer or dimer from single crystal were calculated at the level of M06-2X-D3^[2]^/6-311G(d,p). The minimum energy and stabilization energy of the monomer or dimer from single crystal were calculated at the level of M06-2X-D3/6-311G(d,p). Geometries in the crystal phase at ground state were calculated based on a combined quantum mechanics and molecular mechanics (QM/MM) model selected from 3×3×3 supercells of S-NAP dimer, R-NAP dimer, S-NAP+TCNB and R-NAP+TCNB, as well as 2.5×3×3 supercells of rac-NAP.^[3]^ The central molecule was treated as the QM part at M06-2X-D3/6-311G(d,p) level and the surrounding molecules were frozen and acted as the MM part with the universal force field (UFF). On the basis of the optimized geometries, time-dependent density functional theory (TD-DFT)^[4]^ was utilized at the M06-2X-D3/6-311G(d,p) level to calculate the optimized excited (S_1_) geometries and energy levels. The natural transition orbitals (NTOs) analysis was further carried out by Multiwfn^[5]^ to obtain the main NTOs contributing to the electron transition. IGM analysis^[6,7]^ of weak interaction based on single crystal structure was conducted by using Multiwfn. The corresponding structure and IGM isosurfaces were generated using VMD.^[8]^

**Synthesis Procedure**

## 2.1 Synthesis and Characterization

**Scheme S1.** General synthetic procedure for the preparation of S-NAP-ME and R-NAP-ME.

**Synthesis of S-NAP-ME**

S-naproxen (1.1513 g, 5 mmol) was added into 100 mL round-bottomed flask, and 30 mL methanol was added for ultrasonic shock. After dissolution, 500 μL concentrated sulfuric acid was added slowly drop by drop as catalyst, and then heated in 65^o^C oil bath for reflux for 4 hours. After the solution was cooled to room temperature, the solvent was evaporated under vacuum and purified with petroleum ether/dichloromethane 4:1 as eluent by silica gel column chromatography to obtain white powder solid. (yield: 0.92 g, 76%). ^1^H NMR (400 MHz, Chloroform-d) δ 7.71 (s, 1H), 7.69 (s, 1H), 7.66 (d, *J* = 1.8 Hz, 1H), 7.41 (d, *J* = 1.8 Hz, 1H), 7.13 (d, *J* = 8.8 Hz, 1H), 7.11 (s, 1H), 3.91 (s, 3H), 3.86 (d, *J* = 7.2 Hz, 1H), 3.66 (s, 3H), 1.57 (s, 3H). ^13^C NMR (101 MHz, Chloroform-d) δ 175.16, 157.66, 135.68, 133.71, 129.27, 128.95, 127.18, 126.19, 125.94, 118.99, 105.62, 55.32, 52.04, 45.36, 18.59. HRMS [M+H]^+^ calcd 245.1173, found 245.1172.

**Synthesis of R-NAP-ME**

R-naproxen (1.1513 g, 5 mmol) was added into 100 mL round-bottomed flask, and 30 mL methanol was added for ultrasonic shock. After dissolution, 500 μL concentrated sulfuric acid was added slowly drop by drop as catalyst, and then heated in 65^o^C oil bath for reflux for 4 hours. After the solution was cooled to room temperature, the solvent was evaporated under vacuum and purified with petroleum ether/dichloromethane 4:1 as eluent by silica gel column chromatography to obtain white powder solid. (yield: 0.87 g, 71%). ^1^H NMR (400 MHz, Chloroform-d) δ 7.71 (s, 1H), 7.69 (s, 1H), 7.66 (d, *J* = 1.9 Hz, 1H), 7.41 (d, *J* = 1.8 Hz, 1H), 7.13 (d, *J* = 8.8 Hz, 1H), 7.11 (s, 1H), 3.91 (s, 3H), 3.86 (d, *J* = 7.2 Hz, 1H), 3.66 (s, 3H), 1.57 (d, *J* = 2.2 Hz, 3H). ^13^C NMR (101 MHz, Chloroform-d) δ 175.15, 157.66, 135.68, 133.71, 129.27, 128.94, 127.17, 126.19, 125.94, 118.99, 105.62, 55.32, 52.04, 45.36, 18.59. HRMS [M+H]^+^ calcd 245.1173, found 245.1172.

**Scheme S2.** General synthetic procedure for the preparation of S-NAP-EE and R-NAP-EE.

**Synthesis of S-NAP-EE**

S-naproxen (1.1513 g, 5 mmol) was added into 100 mL round-bottomed flask, and 30 mL ethanol absolute was added for ultrasonic shock. After dissolution, 500 μL concentrated sulfuric acid was added slowly drop by drop as catalyst, and then heated in 80^o^C oil bath for reflux for 4 hours. After the solution was cooled to room temperature, the solvent was evaporated under vacuum and purified with petroleum ether/dichloromethane 5:1 as eluent by silica gel column chromatography to obtain white powder solid. (yield: 0.93 g, 72%). ^1^H NMR (400 MHz, Chloroform-d) δ 7.71 (d, *J* = 1.9 Hz, 1H), 7.69 (d, *J* = 1.4 Hz, 1H), 7.66 (d, *J* = 1.8 Hz, 1H), 7.42 (d, *J* = 1.9 Hz, 1H), 7.14 (s, 1H), 7.11 (t, *J* = 2.9 Hz, 1H), 4.13 (dd, *J* = 11.5, 7.1 Hz, 2H), 3.91 (s, 3H), 3.83 (d, *J* = 7.2 Hz, 1H), 1.56 (d, *J* = 5.4 Hz, 3H), 1.20 (t, *J* = 7.1 Hz, 3H). ^13^C NMR (101 MHz, Chloroform-d) δ 174.70, 157.62, 135.86, 133.67, 129.28, 128.95, 127.09, 126.26, 125.91, 118.92, 105.62, 60.74, 55.31, 45.50, 18.60, 14.13. HRMS [M+H]^+^ calcd 259.1331, found 259.1329.

**Synthesis of R-NAP-EE**

R-naproxen (1.1513 g, 5 mmol) was added into 100 mL round-bottomed flask, and 30 mL ethanol absolute was added for ultrasonic shock. After dissolution, 500 μL concentrated sulfuric acid was added slowly drop by drop as catalyst, and then heated in 80^o^C oil bath for reflux for 4 hours. After the solution was cooled to room temperature, the solvent was evaporated under vacuum and purified with petroleum ether/dichloromethane 5:1 as eluent by silica gel column chromatography to obtain white powder solid. (yield: 0.89 g, 69%). ^1^H NMR (400 MHz, Chloroform-d) δ 7.71 (d, *J* = 1.9 Hz, 1H), 7.69 (s, 1H), 7.66 (d, *J* = 1.8 Hz, 1H), 7.42 (d, *J* = 1.8 Hz, 1H), 7.13 (d, *J* = 8.7 Hz, 1H), 7.11 (d, *J* = 2.5 Hz, 1H), 4.13 (d, *J* = 11.5, 7.1 Hz, 2H), 3.91 (s, 3H), 3.83 (d, *J* = 7.2 Hz, 1H), 1.56 (s, 3H), 1.20 (t, *J* = 7.1 Hz, 3H). ^13^C NMR (101 MHz, Chloroform-d) δ 174.70, 157.62, 135.86, 133.67, 129.28, 128.95, 127.09, 126.26, 125.91, 118.92, 105.62, 60.75, 55.31, 45.50, 18.60, 14.13. HRMS [M+H]^+^ calcd 259.1330, found 259.1329.

**Scheme S3.** General synthetic procedure for the preparation of S-NAP-PE and R-NAP-PE.

**Synthesis of S-NAP-PE**

S-naproxen (1.1513 g, 5 mmol) was added into 100 mL round-bottomed flask, and 35 mL n-propanol was added for ultrasonic shock. After dissolution, 500 μL concentrated sulfuric acid was added slowly drop by drop as catalyst, and then heated in 85^o^C oil bath for reflux for 4 hours. After the solution was cooled to room temperature, the solvent was evaporated under vacuum and purified with petroleum ether/dichloromethane 10:1 as eluent by silica gel column chromatography to obtain white powder solid. (yield: 0.95 g, 70%). ^1^H NMR (400 MHz, Chloroform-d) δ 7.71 (d, *J* = 2.1 Hz, 1H), 7.68 (s, 1H), 7.67 (d, *J* = 2.2 Hz, 1H), 7.41 (d, *J* = 8.4 Hz, 1H), 7.14 (s, 1H), 7.11 (d, *J* = 2.6 Hz, 1H), 4.03 (t, *J* = 6.6 Hz, 2H), 3.91 (d, *J* = 2.2 Hz, 3H), 3.85 (d, *J* = 7.2 Hz, 1H), 1.60 (s, 2H), 1.56 (d, *J* = 2.3 Hz, 3H), 0.87 - 0.82 (m, 3H). ^13^C NMR (101 MHz, Chloroform-d) δ 174.76, 157.61, 135.87, 133.66, 129.27, 128.95, 127.06, 126.29, 125.91, 118.91, 105.62, 66.34, 55.31, 45.54, 21.94, 18.53, 10.28. HRMS [M+H]^+^ calcd 273.1486, found 273.1485.

**Synthesis of R-NAP-PE**

R-naproxen (1.1513 g, 5 mmol) was added into 100 mL round-bottomed flask, and 35 mL n-propanol was added for ultrasonic shock. After dissolution, 500 μL concentrated sulfuric acid was added slowly drop by drop as catalyst, and then heated in 85^o^C oil bath for reflux for 4 hours. After the solution was cooled to room temperature, the solvent was evaporated under vacuum and purified with petroleum ether/dichloromethane 10:1 as eluent by silica gel column chromatography to obtain white powder solid. (yield: 0.92g, 68%). ^1^H NMR (400 MHz, Chloroform-d) δ 7.71 (d, *J* = 2.0 Hz, 1H), 7.69 (s, 1H), 7.67 (d, *J* = 1.8 Hz, 1H), 7.42 (d, *J* = 1.9 Hz, 1H), 7.13 (d, *J* = 8.7 Hz, 1H), 7.11 (d, *J* = 2.5 Hz, 1H), 4.02 (dd, *J* = 6.6, 1.6 Hz, 2H), 3.91 (s, 3H), 3.85 (d, *J* = 7.2 Hz, 1H), 1.61 (d, *J* = 7.0 Hz, 2H), 1.57 (d, *J* = 2.4 Hz, 3H), 0.85 (t, *J* = 7.4 Hz, 3H). ^13^C NMR (101 MHz, Chloroform-d) δ 174.76, 157.61, 135.88, 133.66, 129.27, 128.95, 127.06, 126.29, 125.91, 118.91, 105.62, 66.34, 55.31, 45.54, 21.93, 18.53, 10.28. HRMS [M+H]^+^ calcd 273.1487, found 273.1485.

**Scheme S4.** General synthetic procedure for the preparation of S-NAP-BE and R-NAP-BE.

**Synthesis of S-NAP-BE**

S-naproxen (1.1513 g, 5 mmol) was added into 100 mL round-bottomed flask, and 35 mL n-butanol was added for ultrasonic shock. After dissolution, 500 μL concentrated sulfuric acid was added slowly drop by drop as catalyst, and then heated in 85^o^C oil bath for reflux for 4 hours. After the solution was cooled to room temperature, the solvent was evaporated under vacuum and purified with petroleum ether/dichloromethane 5:1 as eluent by silica gel column chromatography to obtain white powder solid. (yield :0.96 g, 67%). ^1^H NMR (400 MHz, Chloroform-d) δ 7.72 - 7.70 (m, 1H), 7.69 (s, 1H), 7.66 (s, 1H), 7.42 (d, *J* = 1.9 Hz, 1H), 7.15 - 7.12 (m, 1H), 7.11 (s, 1H), 4.10 - 4.04 (m, 2H), 3.91 (s, 3H), 3.84 (d, *J* = 7.1 Hz, 1H), 1.59 (s, 2H), 1.56 (d, *J* = 3.2 Hz, 3H), 1.32 - 1.25 (m, 2H), 0.86 (t, *J* = 7.4 Hz, 3H). ^13^C NMR (101 MHz, Chloroform-d) δ 174.77, 157.61, 135.86, 133.66, 129.27, 128.94, 127.06, 126.28, 125.91, 118.91, 105.61, 64.64, 55.31, 45.54, 30.59, 19.03, 18.54, 13.63. HRMS [M+H]^+^ calcd 287.1639, found 287.1642.

**Synthesis of R-NAP-BE**

R-naproxen (1.1513 g, 5 mmol) was added into 100 mL round-bottomed flask, and 35 mL n-butanol was added for ultrasonic shock. After dissolution, 500 μL concentrated sulfuric acid was added slowly drop by drop as catalyst, and then heated in 85^o^C oil bath for reflux for 4 hours. After the solution was cooled to room temperature, the solvent was evaporated under vacuum and purified with petroleum ether/dichloromethane 5:1 as eluent by silica gel column chromatography to obtain white powder solid. (yield :1.00 g, 70%). ^1^H NMR (400 MHz, Chloroform-d) δ 7.71 (d, *J* = 1.7 Hz, 1H), 7.69 (s, 1H), 7.66 (d, *J* = 1.8 Hz, 1H), 7.42 (d, *J* = 1.9 Hz, 1H), 7.15 - 7.12 (m, 1H), 7.11 (s, 1H), 4.10 - 4.05 (m, 2H), 3.91 (s, 3H), 3.84 (d, *J* = 7.2 Hz, 1H), 1.59 (s, 2H), 1.55 (d, *J* = 11.0 Hz, 3H), 1.32 - 1.25 (m, 2H), 0.86 (t, *J* = 7.4 Hz, 3H). ^13^C NMR (101 MHz, Chloroform-d) δ 174.77, 157.60, 135.86, 133.66, 129.27, 128.94, 127.06, 126.28, 125.90, 118.91, 105.61, 64.64, 55.31, 45.54, 30.59, 19.03, 18.54, 13.63. HRMS [M+H]^+^ calcd 287.1641, found 287.1642.

## 2.2 Crystal Growth and Sample Preparation

**Preparation of cocrystals.** Different configurations of cocrystals were obtained by solvent evaporation method. In a typical experiment, equimolar amounts of S-NAP (23.0 mg) and TCNB (17.8 mg) were dispersed in 10 mL of CH_3_CN using ultrasound to obtain a yellow solution. The cocrystals were obtained after evaporation of solvent from the solution after several days at ambient conditions.

**Preparation of single crystals.** Single crystals of different configurations were obtained by gas phase diffusion method. In a typical experiment, equimolar amounts of S-NAP (9.2 mg) and TCNB (7.1 mg) were dispersed in 4 mL of CH_3_CN using ultrasound to obtain a yellow solution, and the solution was passed through with a 0.45 μm syringe filter and transferred into a glass vial. The glass vial was placed in a sealed glass vial containing 8 mL of water under ambient conditions for several days, and high-quality single crystals were obtained by mutual diffusion of good and poor solvents.

**Method for preparing samples for testing CD spectra**. The dry solid sample was mixed with potassium bromide (KBr) at the ratio of 1:100, then finely ground, and tested at room temperature with KBr as a blank control.

**Supplementary Figures and Tables**


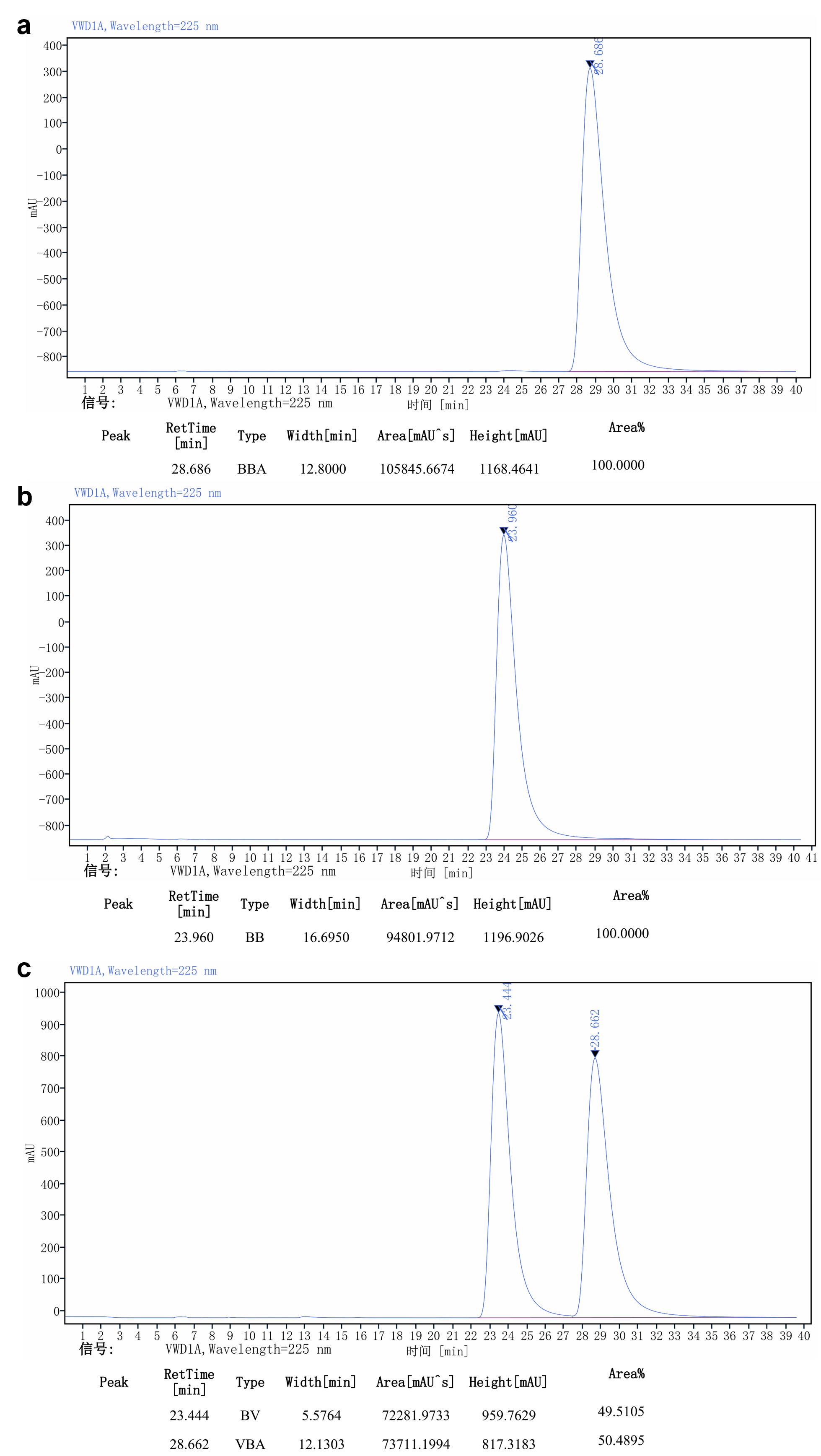


**Figure S1.** Chiral high performance liquid chromatogram (Chiral HPLC) spectrum of (a) S-NAP, (b) R-NAP and (c) rac-NAP in 10% isopropanol in n-hexane monitored at the onset absorption of 225 nm.


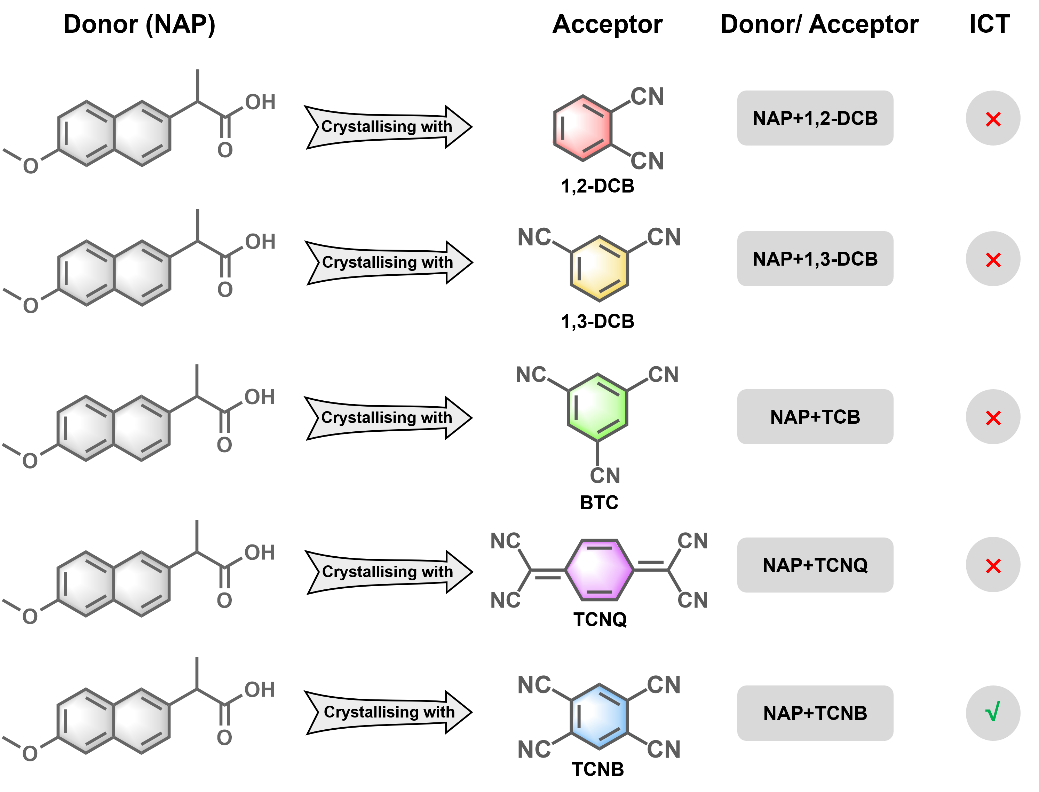


**Figure S2.** Comparison of different acceptor structures of D/A crystal solutions with donor NAP generation ICT. Abbreviations: 1,2-DCB for 1,2-dicyanobenzene, 1,3-DCB for 1,3-dicyanobenzene, BTC for 1,3,5-benzenetricarbonitrile, TCNQ for 7,7,8,8-tetracyanoquinodimethane.


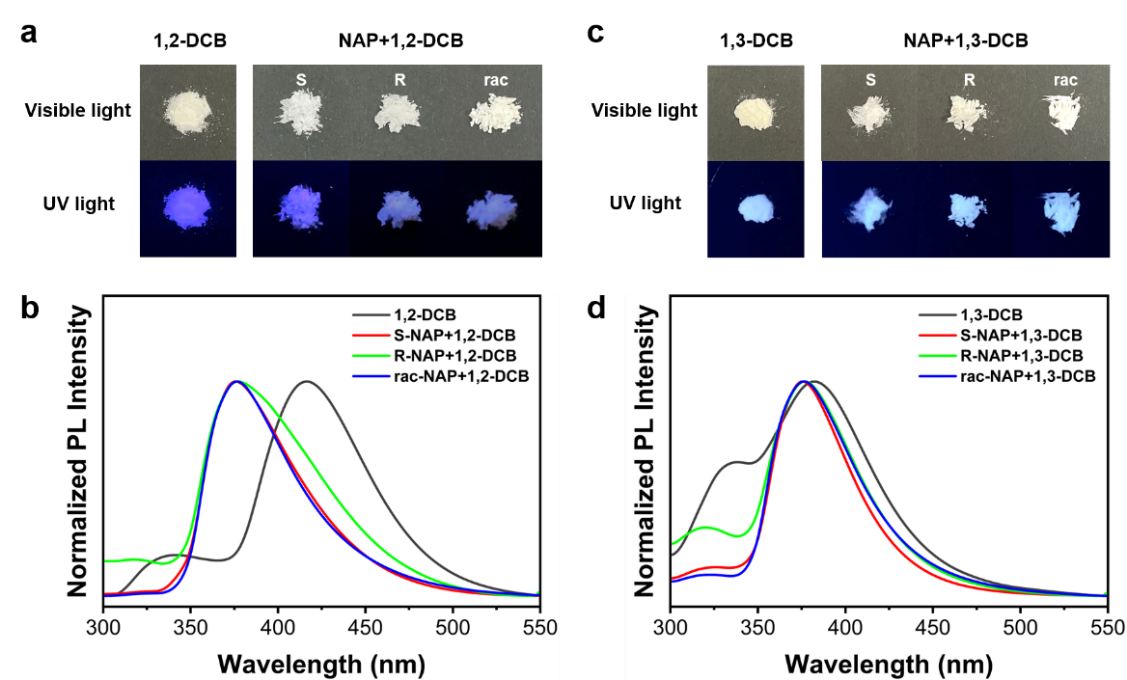


**Figure S3.** (a) Photographs under visible light and UV light (365 nm) and (b) normalized PL spectra of 1,2-DCB, S-NAP+1,2-DCB, R-NAP+1,2-DCB and rac-NAP+1,2-DCB. (c) Photographs under visible light and UV light (365 nm) and (d) normalized PL spectra of 1,3-DCB, S-NAP+1,3-DCB, R-NAP+1,3-DCB and rac-NAP+1,3-DCB.


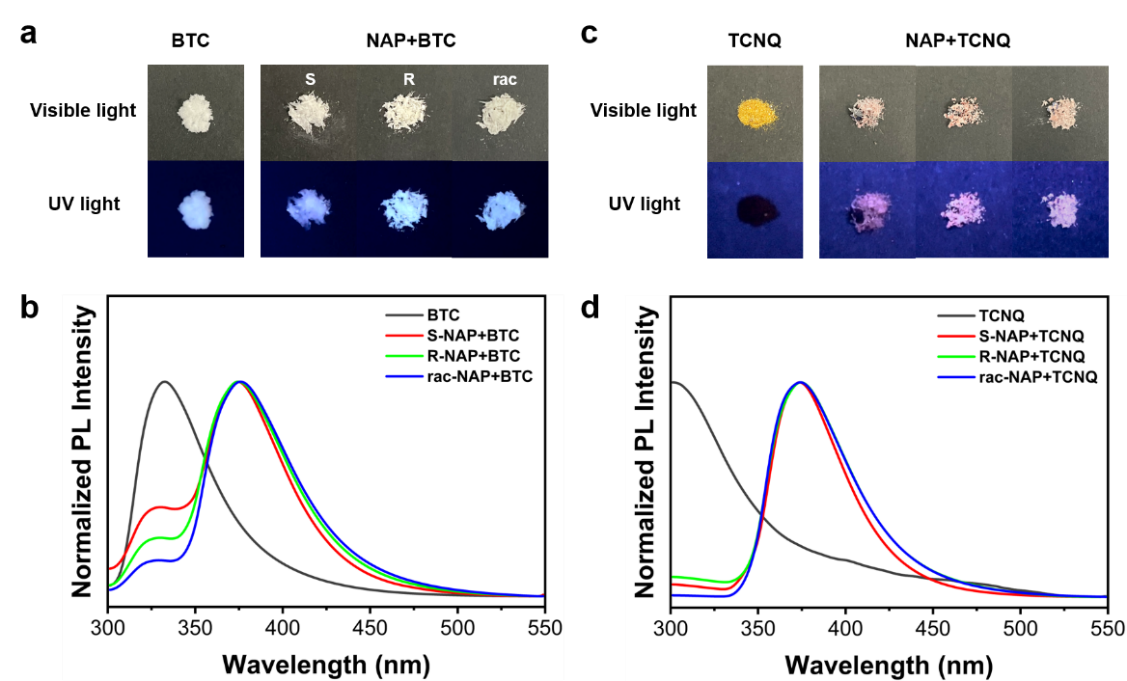


**Figure S4.** (a) Photographs under visible light and UV light (365 nm) and (b) normalized PL spectra of BTC, S-NAP+BTC, R-NAP+BTC and rac-NAP+BTC. (c) Photographs under visible light and UV light (365 nm) and (d) normalized PL spectra of TCNQ, S-NAP+TCNQ, R-NAP+TCNQ and rac-NAP+TCNQ.


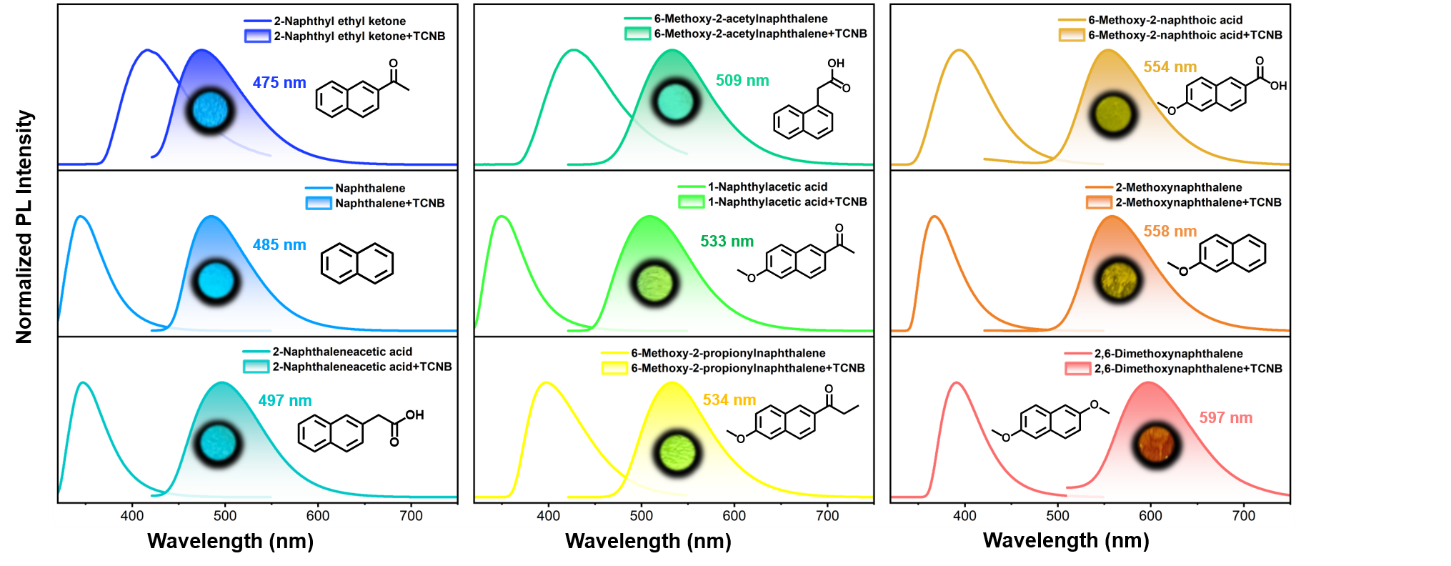


**Figure S5.** Photographs under UV light (365 nm) and normalized PL spectra of nine other donors with structural similarity to NAP after interaction with TCNB.


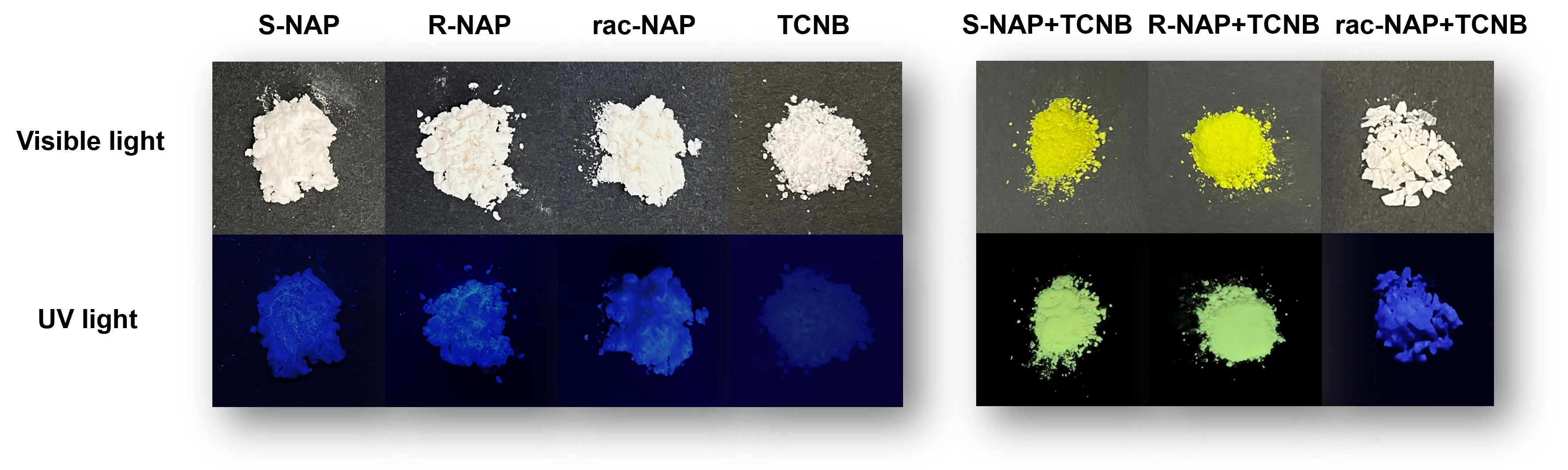


**Figure S6.** Photographs of S-NAP, R-NAP and rac-NAP interaction with TCNB under visible light and UV light (365 nm).


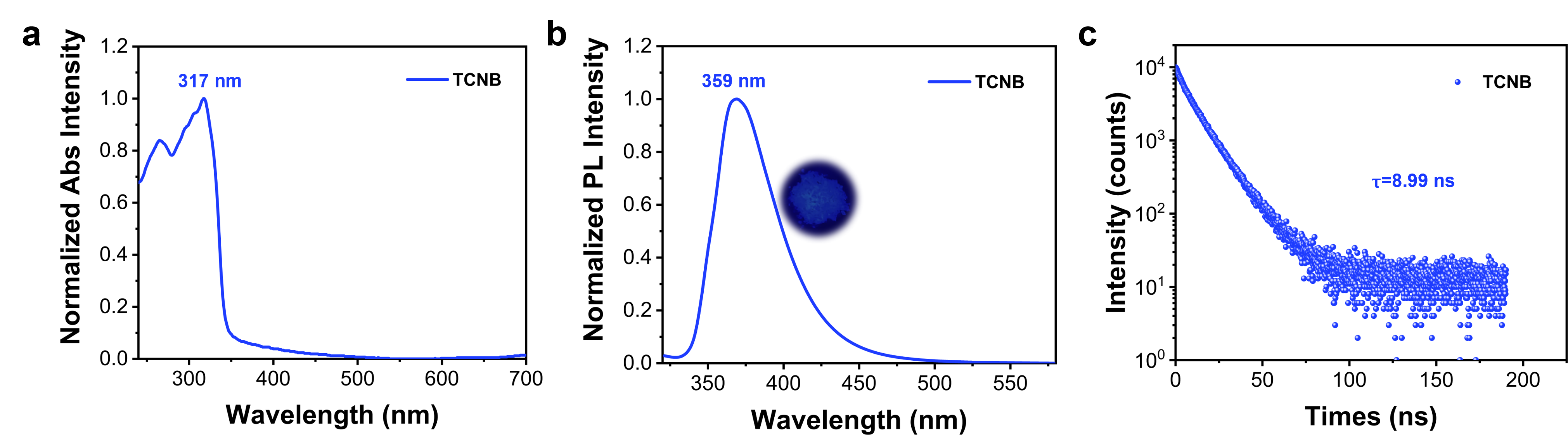


**Figure S7.** Photophysical properties of TCNB. (a) Normalized absorption spectra of TCNB. (b) Normalized PL spectra of TCNB (*λ*_ex_ = 300 nm). (c) Fluorescence decay curve of TCNB.


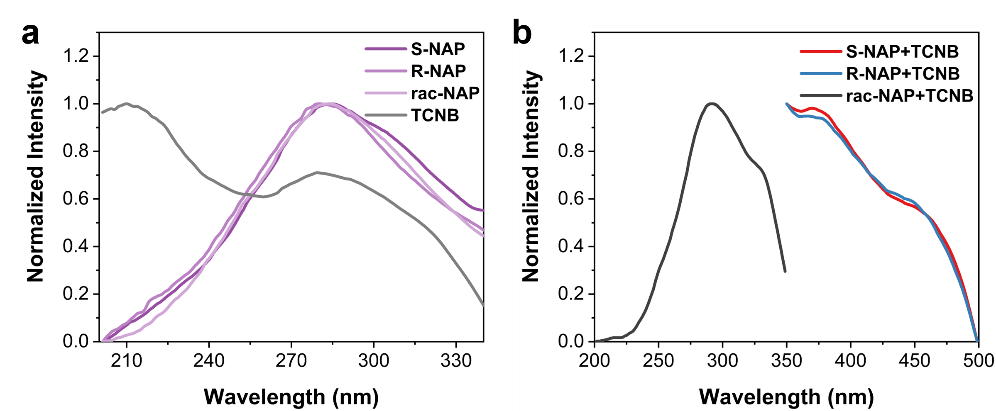


**Figure S8.** a) Excitation spectra of S-NAP (*λ*_em_ = 370 nm), R-NAP (*λ*_em_ = 370 nm), rac-NAP (*λ*_em_ = 370 nm) and TCNB (*λ*_em_ = 370 nm). b) Excitation spectra of S-NAP+TCNB (*λ*_em_ = 530 nm), R-NAP+TCNB (*λ*_em_ = 530 nm) and rac-NAP+TCNB (*λ*_em_ = 370 nm).


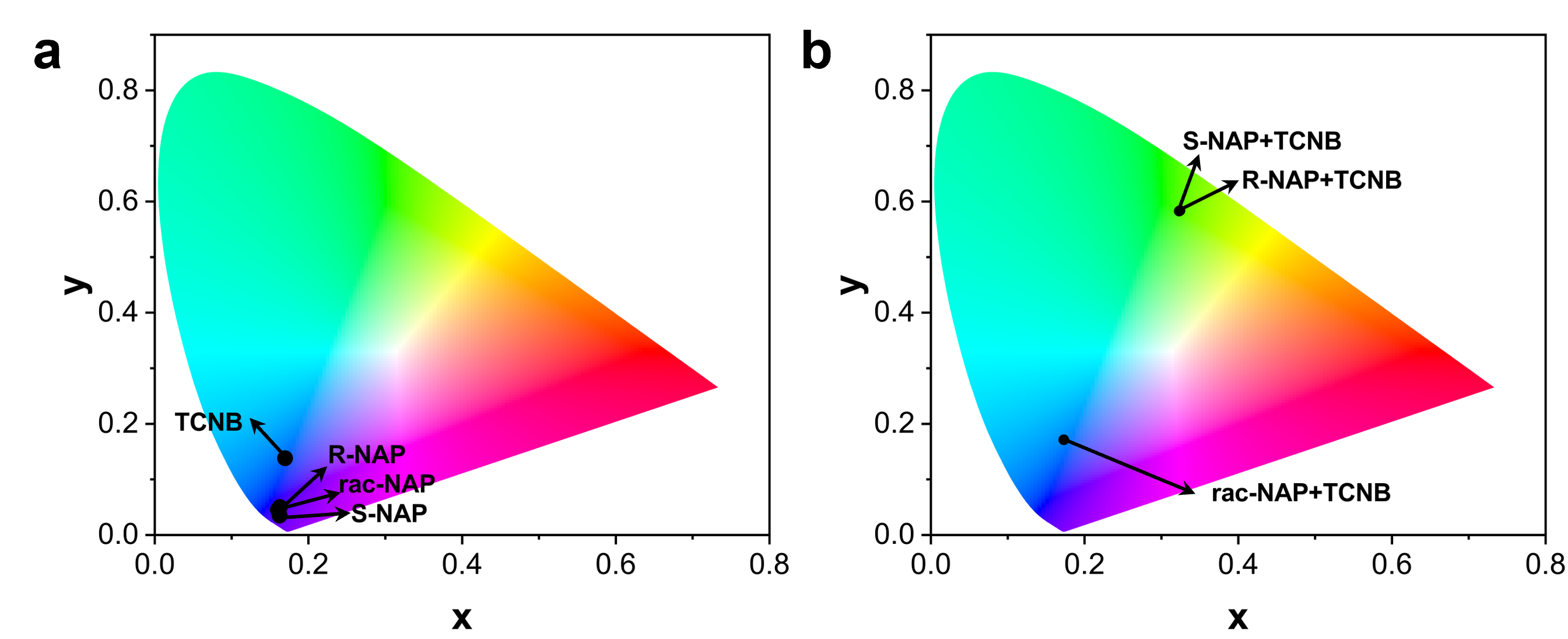


**Figure S9.** a) CIE chromaticity diagram of S-NAP, R-NAP and rac-NAP. b) CIE chromaticity diagram of S-NAP+TCNB, R-NAP+TCNB and rac-NAP+TCNB.





**Figure S10.** FTIR spectra of S-NAP, R-NAP, rac-NAP and TCNB.

**Table S1. Comparison of FTIR data before and after S-NAP, R-NAP and rac-NAP interaction with TCNB.**

| **Samples** | **C=O (cm^-1^) ^[a]^** | **C-H (cm^-1^) ^[b]^** | **C-H (cm^-1^) ^[b]^** | **C≡N (cm^-1^) ^[b]^** | **C=C(cm^-1^) ^[b]^** |
| --- | --- | --- | --- | --- | --- |
| **S-NAP** | 1728 | - | - | - | - |
| **R-NAP** | 1729 | - | - | - | - |
| **rac-NAP** | 1710 | - | - | - | - |
| **TCNB** | - | 3114 | 3048 | 2245 | 1486 |
| **S-NAP+TCNB** | 1747 | 3117 | 3050 | 2244 | 1488 |
| **R-NAP+TCNB** | 1746 | 3117 | 3050 | 2244 | 1488 |
| **rac-NAP+TCNB** | 1711 | 3114 | 3048 | 2245 | 1487 |

[a] Corresponding to FTIR spectra comparison with NAP. [b] Corresponding to FTIR spectra comparison with TCNB.





**Figure S11.** Raman spectra of S-NAP, R-NAP, rac-NAP and TCNB.

**Table S2. Comparison of Raman data before and after S-NAP, R-NAP and rac-NAP interaction with TCNB.**

| **Samples** | **C=O (cm^-1^) ^[a]^** | **C-H (cm^-1^) ^[a]^** | **Ring bend ^[b]^** |
| --- | --- | --- | --- |
| **S-NAP** | - | 1172 | - |
| **R-NAP** | - | 1172 | - |
| **rac-NAP** | - | 1172 | - |
| **TCNB** | - | - | 721 |
| **S-NAP+TCNB** | 1742 | 1181 | 718 |
| **R-NAP+TCNB** | 1742 | 1181 | 718 |
| **rac-NAP+TCNB** | - | 1774 | 720 |

[a] Corresponding to Raman spectra comparison with NAP. [b] Corresponding to Raman spectra comparison with TCNB.


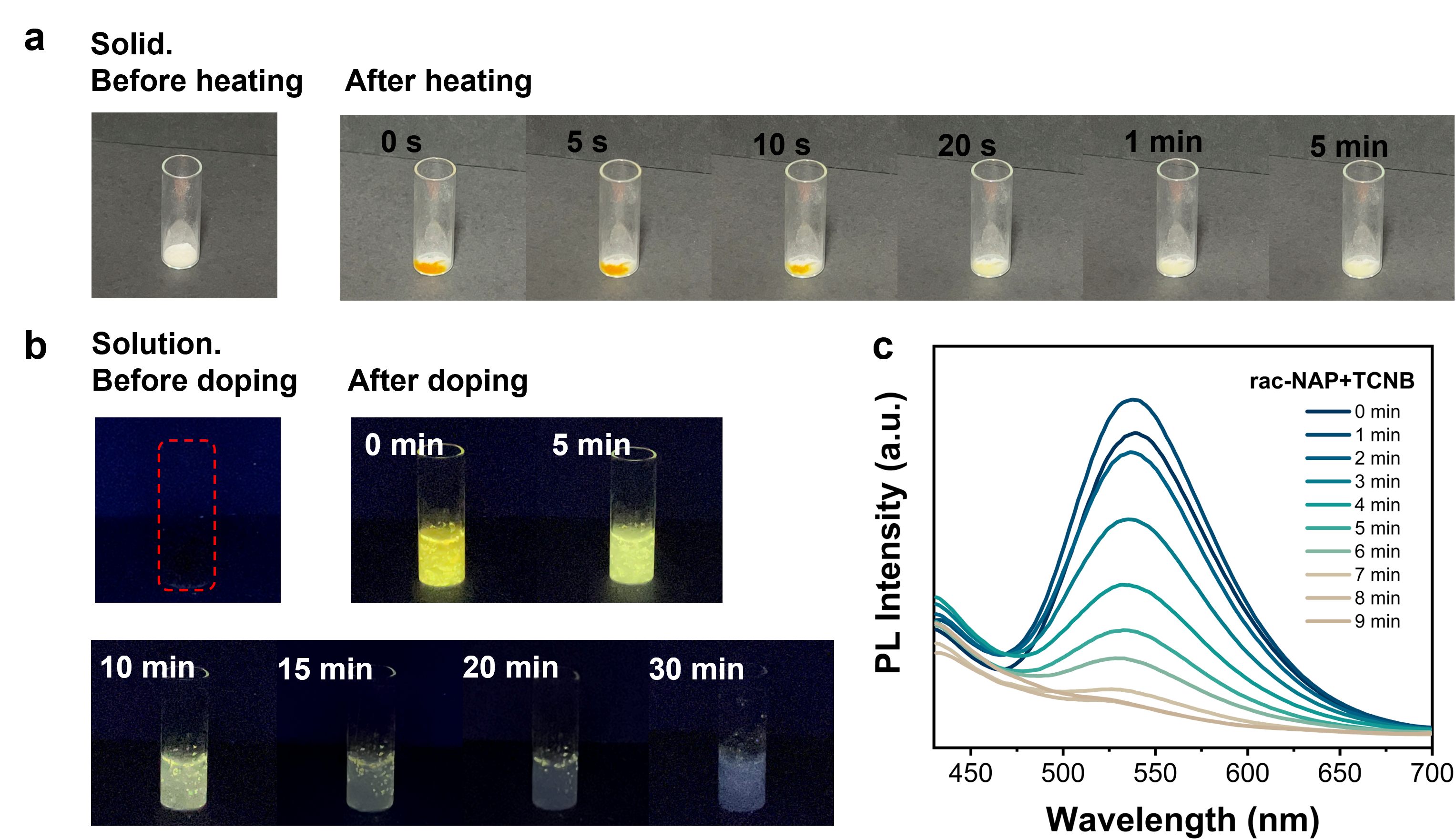


**Figure S12.** (a) Photos of the solid state heat annealing experiments. The specific procedure was to take solid rac-NAP and TCNB in a vial according to the molar ratio of 1:1, heat them at high temperature and then leave them to cool down, then take photographs under visible light. Rapid decay of ICT fluorescence (b) emission and (c) spectra in liquid state of the mixture of rac-NAP and TCNB. The specific procedure was to dissolve rac-NAP and TCNB in acetonitrile solution, transfer them to a vial according to the molar ratio of 1:1, leave them to stand, then take photos under UV light and measure the fluorescence spectra.


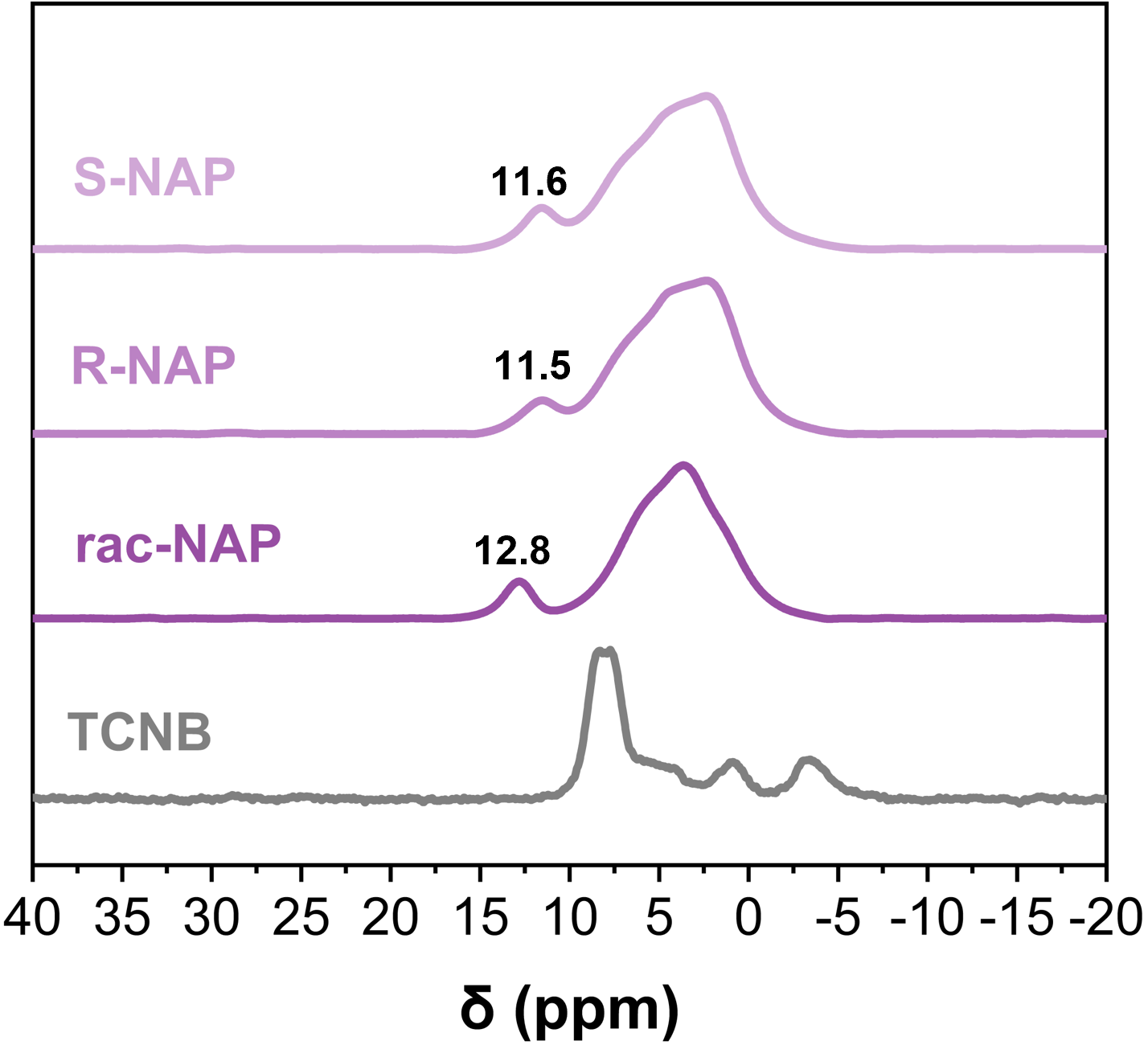


**Figure S13.** Solid-state ^1^H NMR spectra of S-NAP, R-NAP, rac-NAP and TCNB.





**Figure S14.** PXRD patterns of S-NAP, R-NAP, rac-NAP and TCNB, and the calculated XRD from cif file.


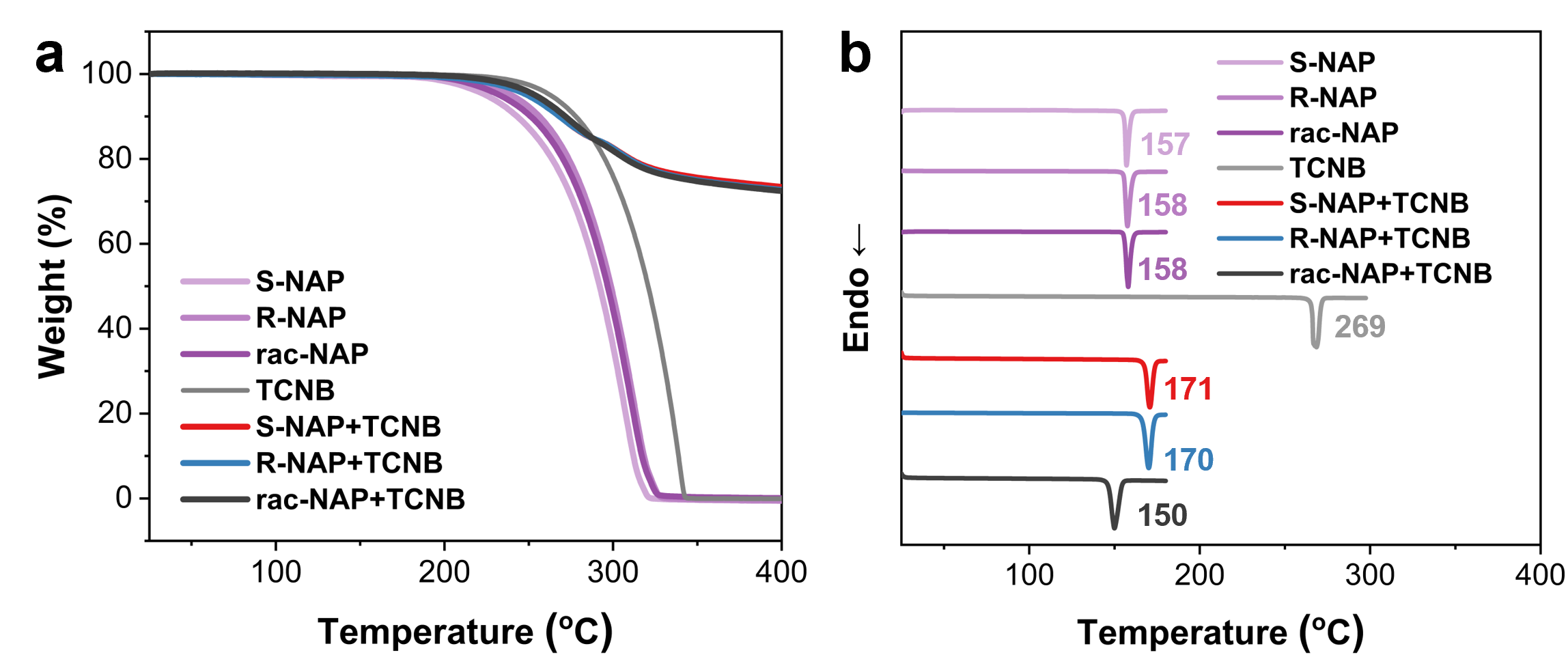


**Figure S15.** (a) TGA and (b) DSC spectra of S-NAP, R-NAP and rac-NAP before and after interaction with TCNB.

| **Table S3. The single crystal X-ray crystallographic data of S-NAP, R-NAP, rac-NAP and TCNB.** | | | | |  |
| --- | --- | --- | --- | --- | --- |
| **Compound** | **S-NAP** | **R-NAP** | **rac-NAP** | **TCNB** |  |
| **Empirical formula** | C_14_H_14_O_3_ | C_14_H_14_O_3_ | C_14_H_14_O_3_ | C_10_H_2_N_4_ |  |
| **Formula weight** | 230.25 | 230.25 | 230.25 | 178.16 |  |
| **Temperature/K** | 99.99(10) | 295.17(10) | 169.99(10) | 295.27(10) |  |
| **Crystal system** | monoclinic | monoclinic | orthorhombic | monoclinic |  |
| **Space group** | P2_1_ | P2_1_ | Pbca | P2_1_/n |  |
| **a/Å** | 7.71690(10) | 7.8804(2) | 15.1715(9) | 6.56890(10) |  |
| **b/Å** | 5.70860(10) | 5.78831(17) | 5.9497(4) | 10.3335(2) |  |
| **c/Å** | 13.3655(2) | 13.3147(4) | 25.7787(16) | 12.7777(2) |  |
| **α/°** | 90 | 90 | 90 | 90 |  |
| **β/°** | 93.742(2) | 93.932(3) | 90 | 96.286(2) |  |
| **γ/°** | 90 | 90 | 90 | 90 |  |
| **Volume/Å^3^** | 587.531(16) | 605.91(3) | 2326.9(3) | 862.13(3) |  |
| **Z** | 2 | 2 | 8 | 4 |  |
| **ρcalcg/cm^3^** | 1.302 | 1.262 | 1.314 | 1.373 |  |
| **μ/mm^‑1^** | 0.742 | 0.720 | 0.749 | 0.739 |  |
| **F(000)** | 244.0 | 244.0 | 976.0 | 360.0 |  |
| **Crystal size/mm^3^** | 0.14 × 0.13 × 0.1 | 0.15 × 0.12 × 0.09 | 0.14 × 0.13 × 0.11 | 0.15 × 0.13 × 0.12 |  |
| **Radiation** | Cu Kα (λ = 1.54184) | Cu Kα (λ = 1.54184) | Cu Kα (λ = 1.54184) | Cu Kα (λ = 1.54184) |  |
| **2θ range for data collection/°** | 6.628 to 142.314 | 6.654 to 151.572 | 6.858 to 147.266 | 11.038 to 150.534 |  |
| **Index ranges** | -7 ≤ h ≤ 9, -6 ≤ k ≤ 3, -16 ≤ l ≤ 15 | -9 ≤ h ≤ 9, -4 ≤ k ≤ 6, -16 ≤ l ≤ 15 | -18 ≤ h ≤ 8, -6 ≤ k ≤ 7, -31 ≤ l ≤ 31 | -7 ≤ h ≤ 8, -4 ≤ k ≤ 12, -15 ≤ l ≤ 15 |  |
| **Reflections collected** | 2491 | 4133 | 7270 | 5930 |  |
| **Independent reflections** | 1485 [R_int_ = 0.0090, R_sigma_ = 0.0125] | 1726 [R_int_ = 0.0312, R_sigma_ = 0.0279] | 2317 [R_int_ = 0.0348, R_sigma_ = 0.0340] | 1733 [R_int_ = 0.0215, R_sigma_ = 0.0169] |  |
| **Data/restraints/parameters** | 1485/1/158 | 1726/1/157 | 2317/0/178 | 1733/0/128 |  |
| **Goodness-of-fit on F^2^** | 1.017 | 1.091 | 1.084 | 1.033 |  |
| **Final R indexes [I>=2σ (I)]** | R_1_ = 0.0264, wR_2_ = 0.0741 | R_1_ = 0.0342, wR_2_ = 0.0913 | R_1_ = 0.0571, wR_2_ = 0.1361 | R_1_ = 0.0384, wR_2_ = 0.1117 |  |
| **Final R indexes [all data]** | R_1_ = 0.0265, wR_2_ = 0.0743 | R_1_ = 0.0406, wR_2_ = 0.1022 | R_1_ = 0.0750, wR_2_ = 0.1477 | R_1_ = 0.0411, wR_2_ = 0.1145 |  |
| **Largest diff. peak/hole / e Å^-3^** | 0.22/-0.12 | 0.12/-0.17 | 0.21/-0.25 | 0.15/-0.16 |  |
| **CCDC Number** | 2303237 | 2303238 | 2303239 | 2303240 |  |

| **Table S4. The single crystal X-ray crystallographic data of S-NAP+TCNB, R-NAP+TCNB and rac-NAP+TCNB.** | | | |
| --- | --- | --- | --- |
| **Compound** | **S-NAP+TCNB** | **R-NAP+TCNB** | **rac-NAP-TCNB** |
| **Empirical formula** | C_24_H_16_N_4_O_3_ | C_24_H_16_N_4_O_3_ | C_14_H_14_O_3_ |
| **Formula weight** | 408.41 | 408.41 | 230.25 |
| **Temperature/K** | 169.99(10) | 169.94(12) | 295.2(3) |
| **Crystal system** | triclinic | triclinic | orthorhombic |
| **Space group** | P1 | P1 | Pbca |
| **a/Å** | 6.7171(4) | 6.7333(5) | 25.8561(7) |
| **b/Å** | 8.4880(6) | 8.4814(7) | 15.4081(4) |
| **c/Å** | 10.5619(7) | 10.5438(10) | 5.9843(2) |
| **α/°** | 100.192(6) | 100.128(7) | 90 |
| **β/°** | 106.753(6) | 106.740(8) | 90 |
| **γ/°** | 108.268(6) | 108.187(7) | 90 |
| **Volume/Å^3^** | 523.37(6) | 523.92(8) | 2384.11(12) |
| **Z** | 1 | 1 | 8 |
| **ρcalcg/cm^3^** | 1.296 | 1.294 | 1.283 |
| **μ/mm^‑1^** | 0.721 | 0.088 | 0.731 |
| **F(000)** | 212.0 | 212.0 | 976.0 |
| **Crystal size/mm^3^** | 0.14 × 0.12 × 0.11 | 0.16 × 0.13 × 0.11 | 0.16 × 0.13 × 0.11 |
| **Radiation** | Cu Kα (λ = 1.54184) | Mo Kα (λ = 0.71073) | Cu Kα (λ = 1.54184) |
| **2θ range for data collection/°** | 9.148 to 147.7 | 5.286 to 59.052 | 6.838 to 154.674 |
| **Index ranges** | -8 ≤ h ≤ 4, -10 ≤ k ≤ 10, -11 ≤ l ≤ 12 | -9 ≤ h ≤ 8, -10 ≤ k ≤ 11, -13 ≤ l ≤ 9 | -31 ≤ h ≤ 32, -10 ≤ k ≤ 19, -7 ≤ l ≤ 7 |
| **Reflections collected** | 3279 | 3939 | 8274 |
| **Independent reflections** | 2361 [R_int_ = 0.0272, R_sigma_ = 0.0435] | 3117 [R_int_ = 0.0216, R_sigma_ = 0.0450] | 2433 [R_int_ = 0.0801, R_sigma_ = 0.0586] |
| **Data/restraints/parameters** | 2361/3/284 | 3117/3/283 | 2433/0/157 |
| **Goodness-of-fit on F^2^** | 1.034 | 1.044 | 1.089 |
| **Final R indexes [I>=2σ (I)]** | R_1_ = 0.0399, wR_2_ = 0.1063 | R_1_ = 0.0475, wR_2_ = 0.1101 | R_1_ = 0.1183, wR_2_ = 0.3003 |
| **Final R indexes [all data]** | R_1_ = 0.0422, wR_2_ = 0.1099 | R_1_ = 0.0535, wR_2_ = 0.1178 | R_1_ = 0.1379, wR_2_ = 0.3119 |
| **Largest diff. peak/hole / e Å^-3^** | 0.23/-0.19 | 0.27/-0.35 | 0.75/-0.45 |
| **CCDC Number** | 2303241 | 2303242 | 2303243 |

**Figure S16.** Molecular packing structures of S-NAP and R-NAP along the directions of a and b axes.


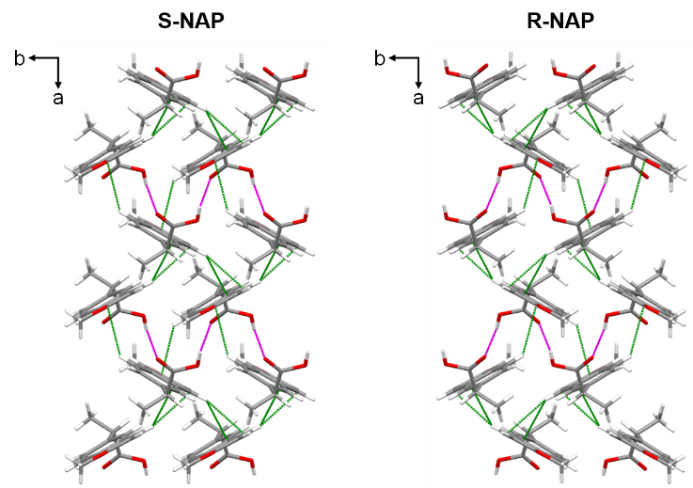


**Table S5. Calculated minimum energy and stabilization energy from single crystal.**

|  | **Minimum energy (eV)** | **Stabilization energy (eV)** |
| --- | --- | --- |
| **S-NAP monomer** | -20724.855 |  |
| **S-NAP dimer** | -41610.884 | -161.174 |
| **R-NAP monomer** | -20712.256 |  |
| **R-NAP dimer** | -41598.339 | -173.827 |
| **S-NAP in rac-RAP** | -20633.316 |  |
| **R-NAP in rac-RAP** | -20633.425 |  |
| **rac-NAP** | -41519.453 | -252.712 |
| **S-NAP in S-NAP+TCNB** | -20467.816 |  |
| **TCNB in S-NAP+TCNB** | -15940.275 |  |
| **S-NAP+TCNB** | -36826.684 | -418.593 |
| **R-NAP in R-NAP+TCNB** | -20463.299 |  |
| **TCNB in R-NAP+TCNB** | -15935.948 |  |
| **R-NAP+TCNB** | -36822.167 | -422.920 |


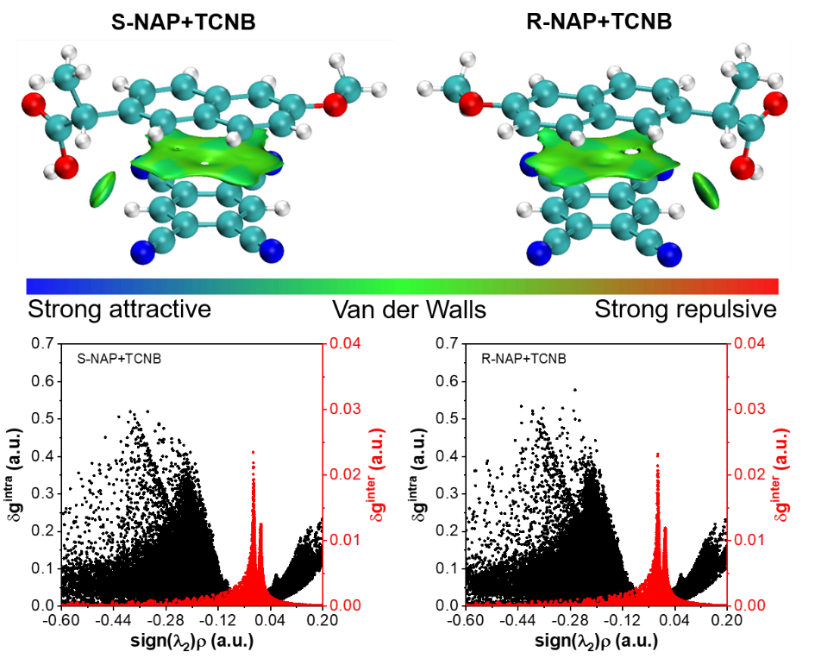


**Figure S17.** The visualized isosurfaces of the IGM analysis for S-NAP+TCNB and R-NAP+TCNB.

**Table S6.** **The contribution of ten atomic pairs with the largest percentage to intermolecular interactions of S-NAP+TCNB and R-NAP+TCNB.**

| **Atomic pair in S-NAP+TCNB** | **Delta-g**  **index** | **Percentage contribution** |  | **Atomic pair in**  **R-NAP+TCNB** | **Delta-g**  **index** | **Percentage contribution** |
| --- | --- | --- | --- | --- | --- | --- |
| 3&39 | 0.112836 | 2.08 % |  | 2&43 | 0.113885 | 2.11 % |
| 11&46 | 0.088623 | 1.63 % |  | 13&40 | 0.088477 | 1.64 % |
| 8&42 | 0.086905 | 1.60 % |  | 8&37 | 0.086034 | 1.59 % |
| 19&38 | 0.082572 | 1.52 % |  | 19&42 | 0.081602 | 1.51 % |
| 19&40 | 0.080976 | 1.49 % |  | 19&44 | 0.079979 | 1.48 % |
| 14&37 | 0.080216 | 1.48 % |  | 14&41 | 0.079502 | 1.47 % |
| 18&38 | 0.077659 | 1.43 % |  | 18&42 | 0.077269 | 1.43 % |
| 12&47 | 0.076934 | 1.42 % |  | 11&47 | 0.076727 | 1.42 % |
| 12&46 | 0.075750 | 1.39 % |  | 11&40 | 0.074959 | 1.39 % |
| 14&36 | 0.075379 | 1.39 % |  | 14&46 | 0.073227 | 1.35 % |

**Table S7. Calculated HOMOs and LUMOs from single crystal.**

|  | **LUMO (eV)** | **HOMO (eV)** | **ΔE_g_ (eV)** |
| --- | --- | --- | --- |
| **S-NAP dimer** | -0.75 | -6.94 | 6.19 |
| **R-NAP dimer** | -0.76 | -6.86 | 6.10 |
| **rac-NAP** | -0.57 | -6.61 | 6.04 |
| **S-NAP+TCNB** | -3.24 | -7.17 | 3.93 |
| **R-NAP+TCNB** | -3.15 | -7.15 | 4.00 |


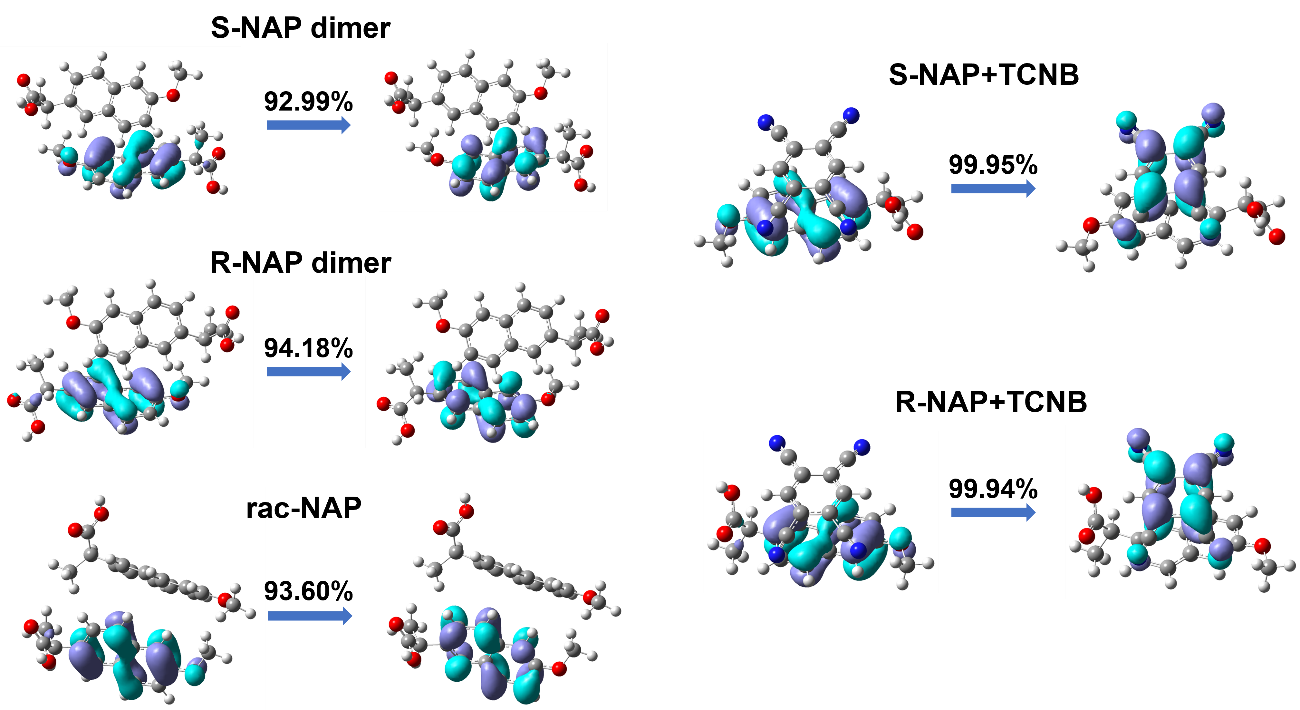


**Figure S18.** The calculated natural transition orbitals (NTOs) of the first singlet excited state (S_1_).

**
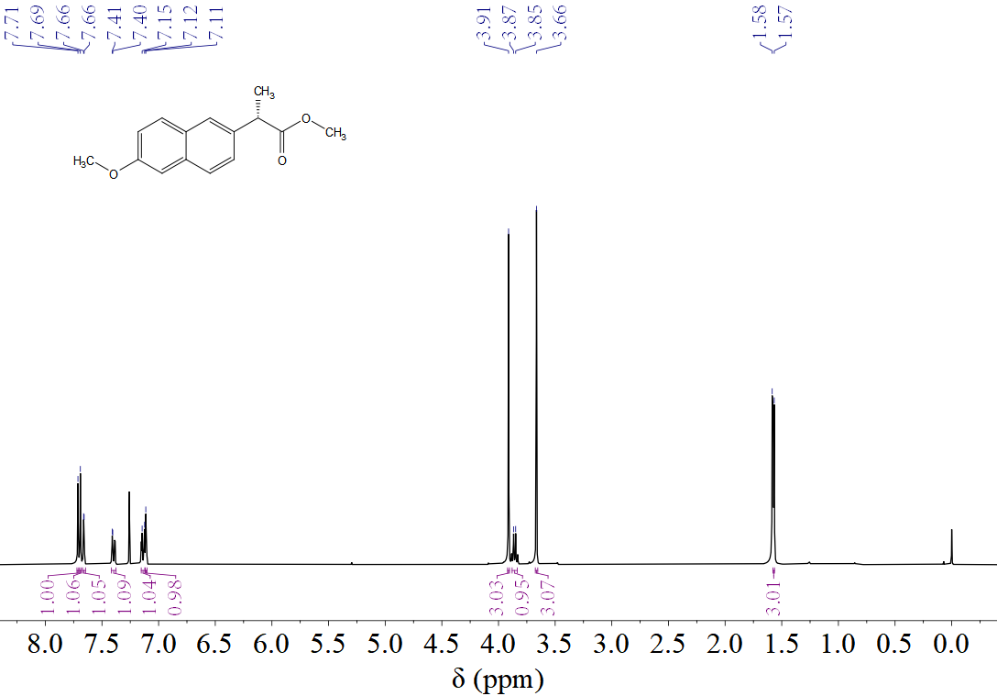
**

**Figure S19.** ^1^H NMR spectrum of S-NAP-ME in CDCl_3_ at room temperature.


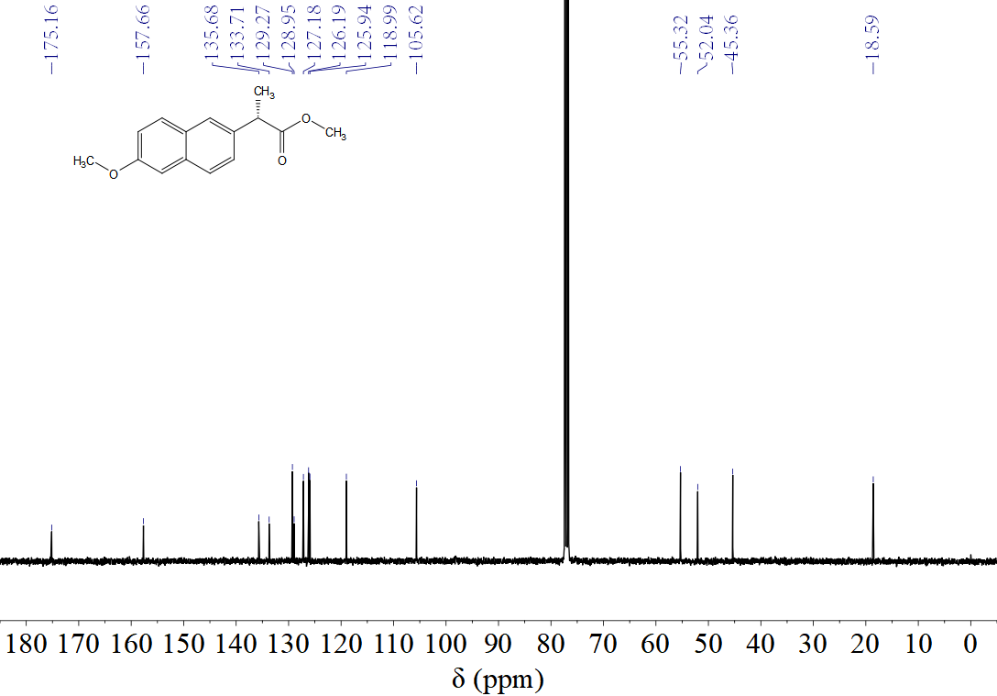


**Figure S20.** ^13^C NMR spectrum of S-NAP-ME in CDCl_3_ at room temperature.


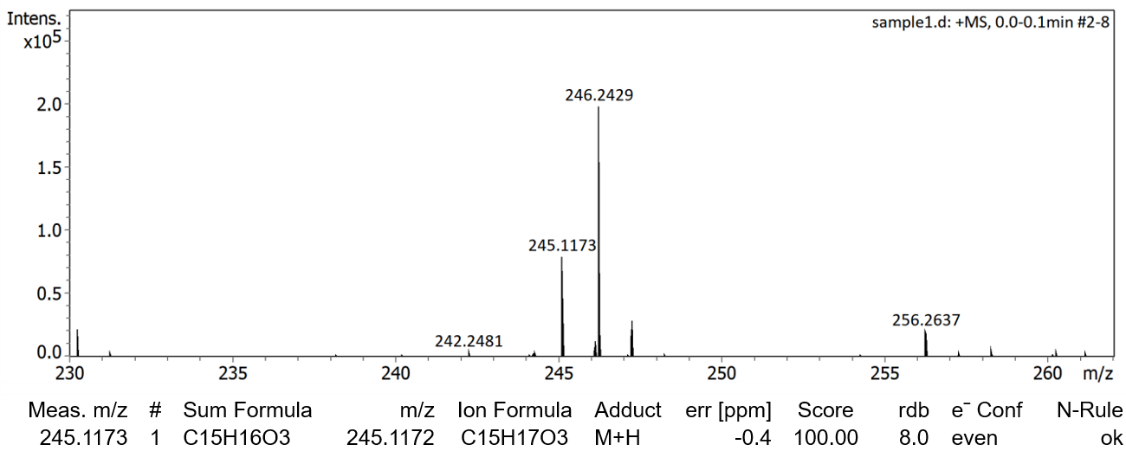


**Figure S21.** HRMS spectrum of S-NAP-ME.


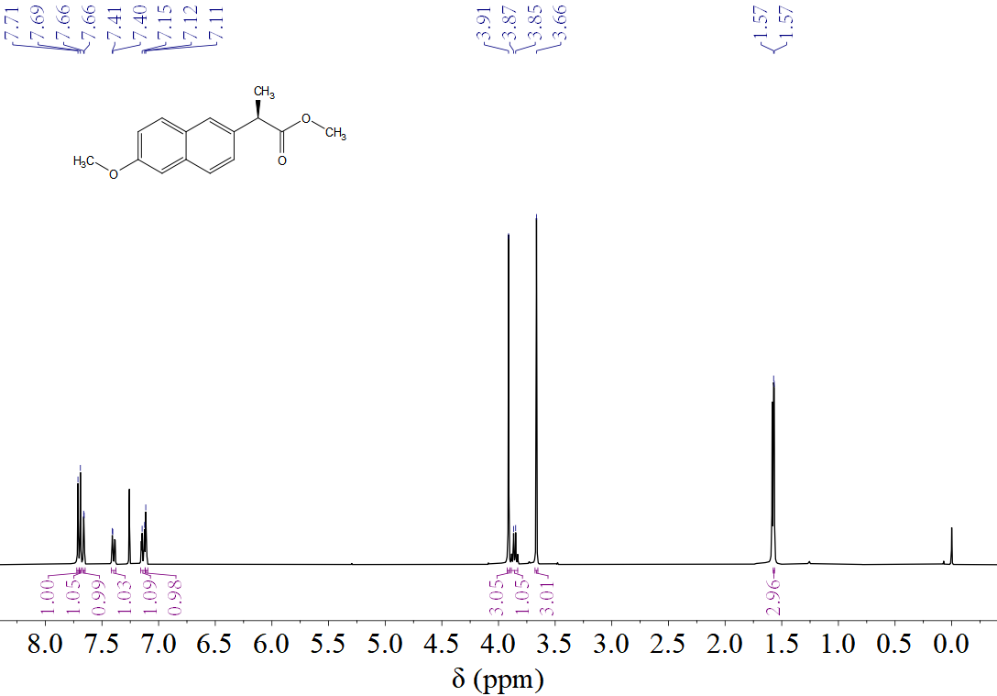


**Figure S22.** ^1^H NMR spectrum of R-NAP-ME in CDCl_3_ at room temperature.


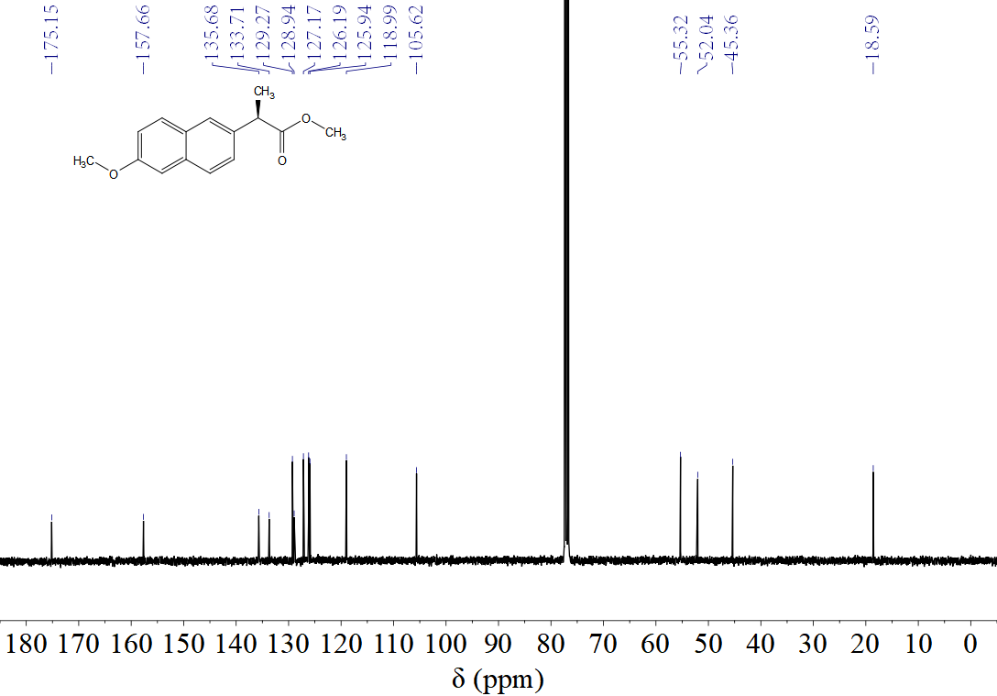


**Figure S23.** ^13^C NMR spectrum of R-NAP-ME in CDCl_3_ at room temperature.

**
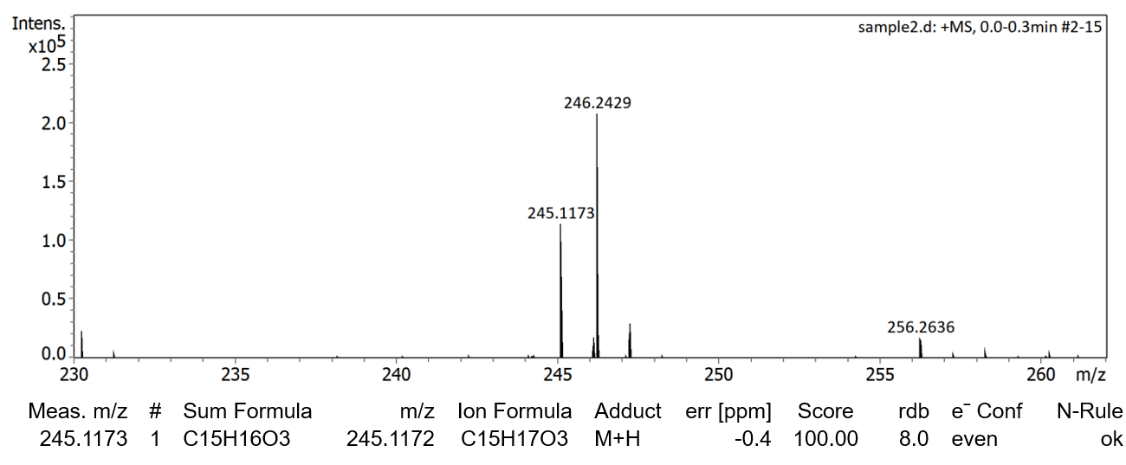
**

**Figure S24.** HRMS spectrum of R-NAP-ME.


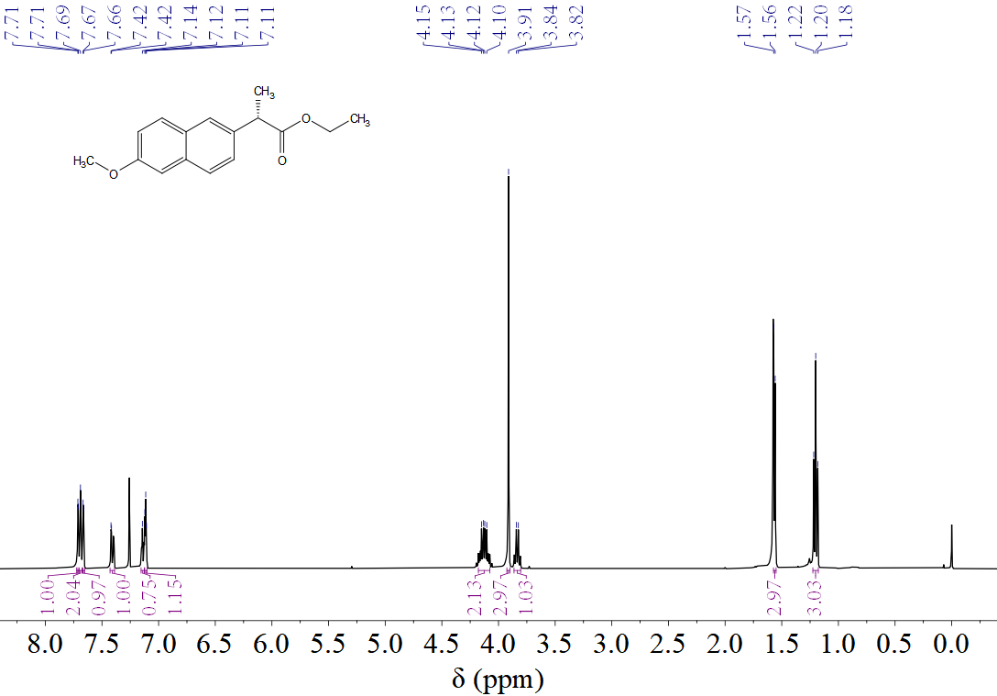


**Figure S25.** ^1^H NMR spectrum of S-NAP-EE in CDCl_3_ at room temperature.


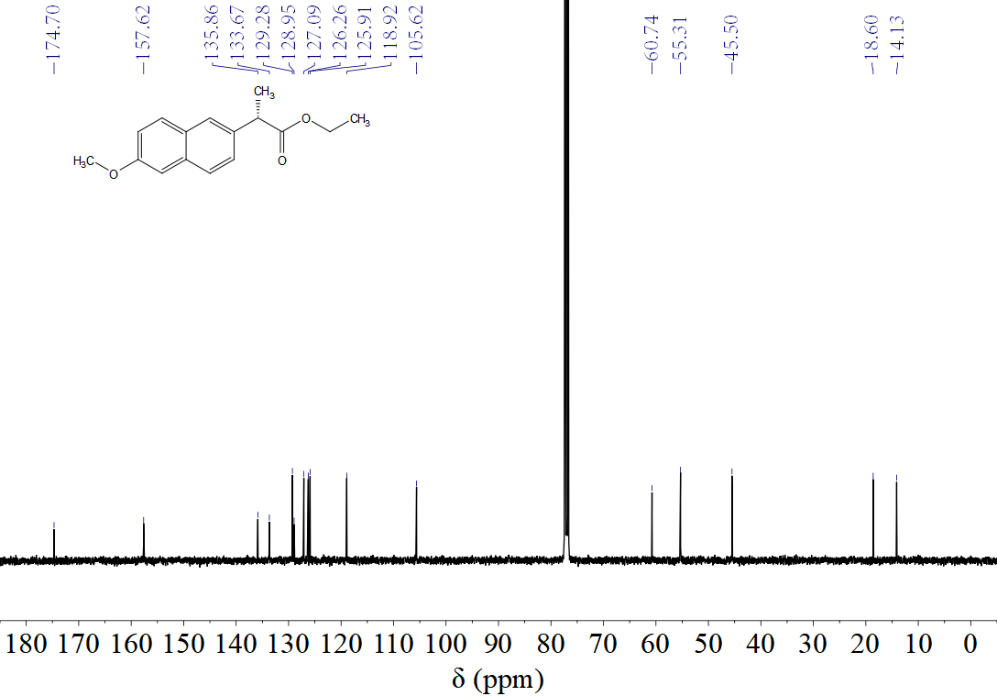


**Figure S26.** ^13^C NMR spectrum of S-NAP-EE in CDCl_3_ at room temperature.


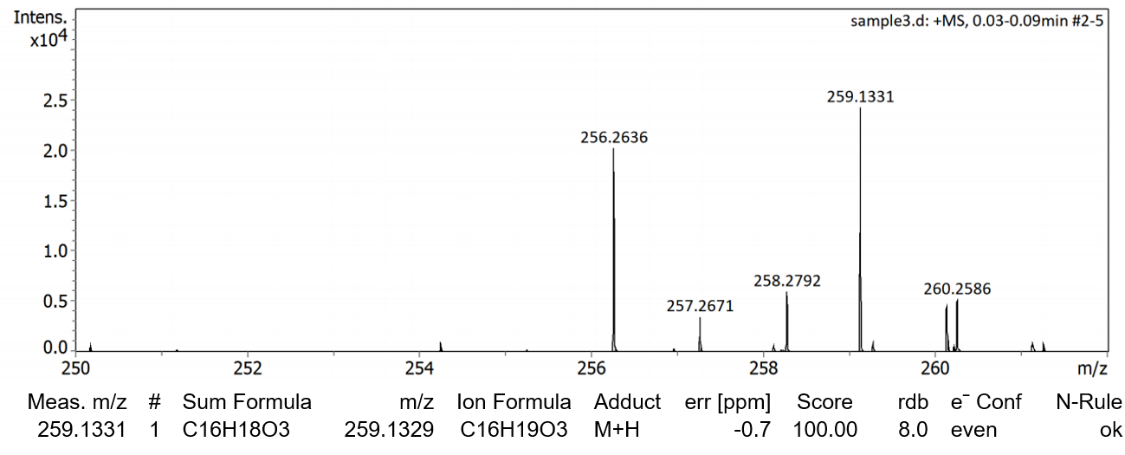


**Figure S27.** HRMS spectrum of S-NAP-EE.


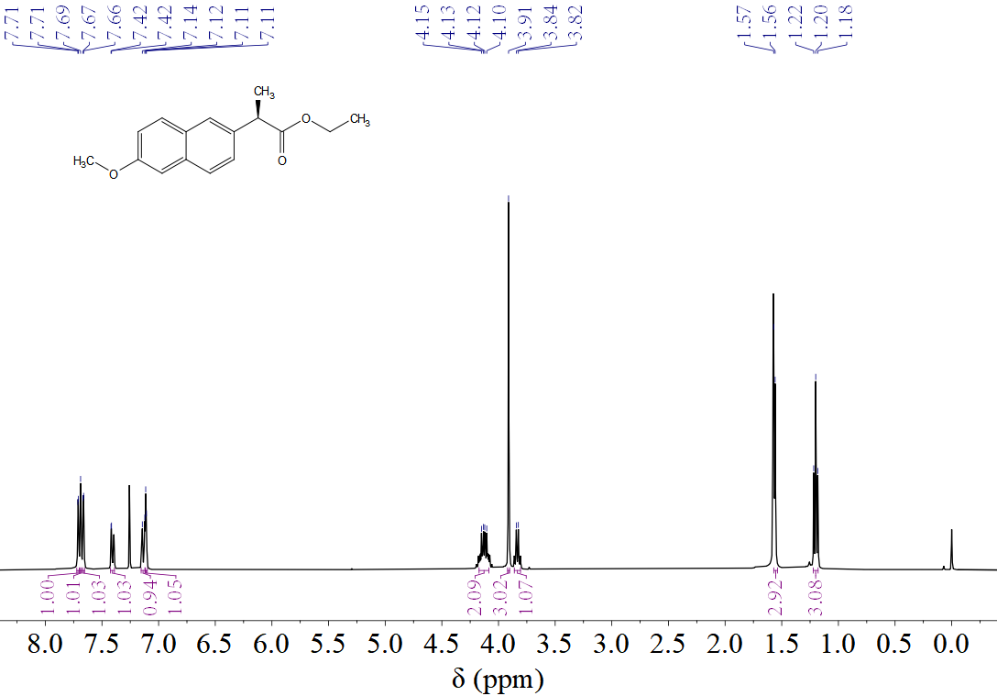


**Figure S28.** ^1^H NMR spectrum of R-NAP-EE in CDCl_3_ at room temperature.


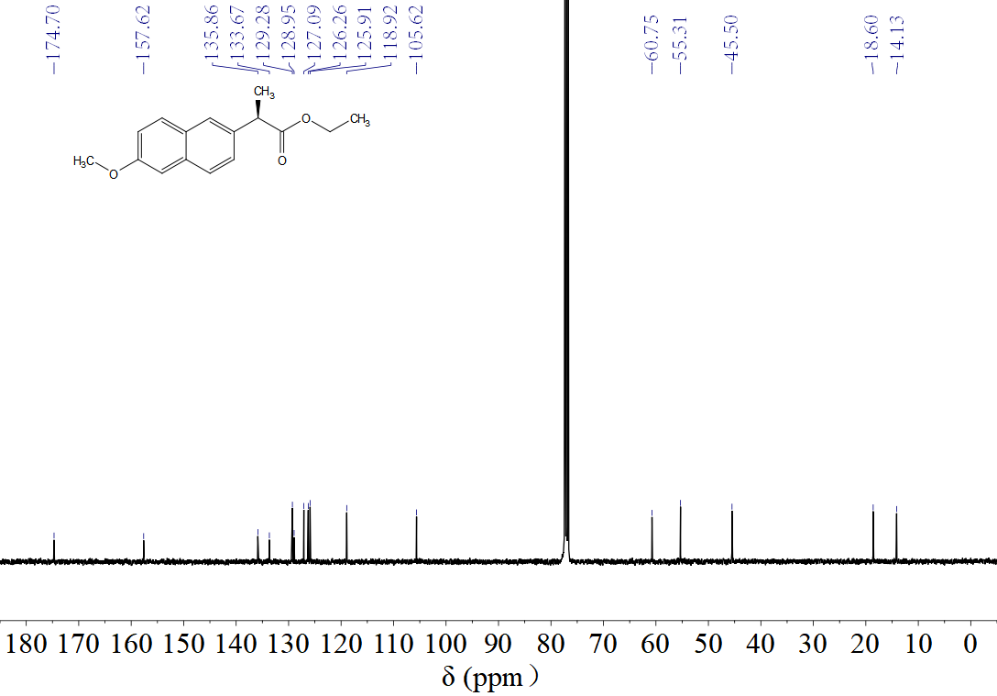


**Figure S29.** ^13^C NMR spectrum of R-NAP-EE in CDCl_3_ at room temperature.


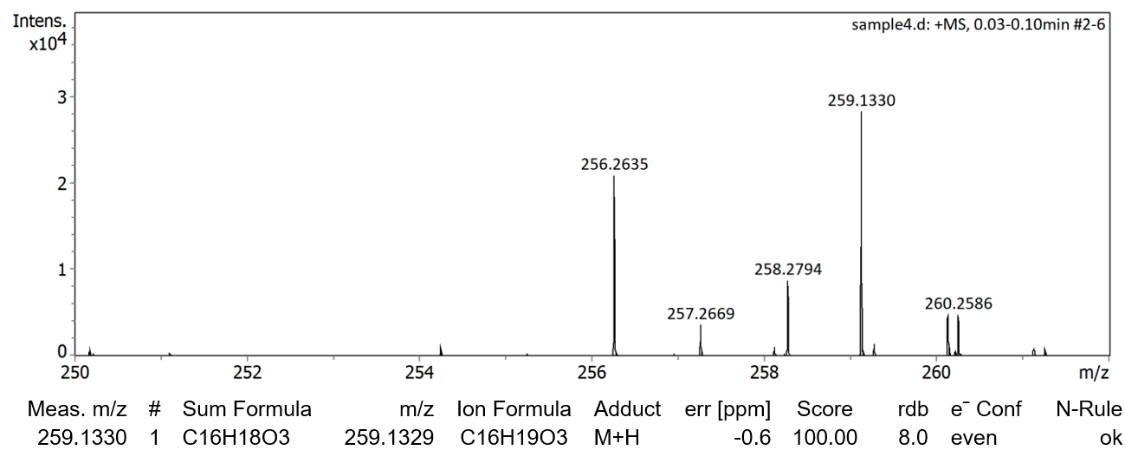


**Figure S30.** HRMS spectrum of R-NAP-EE.


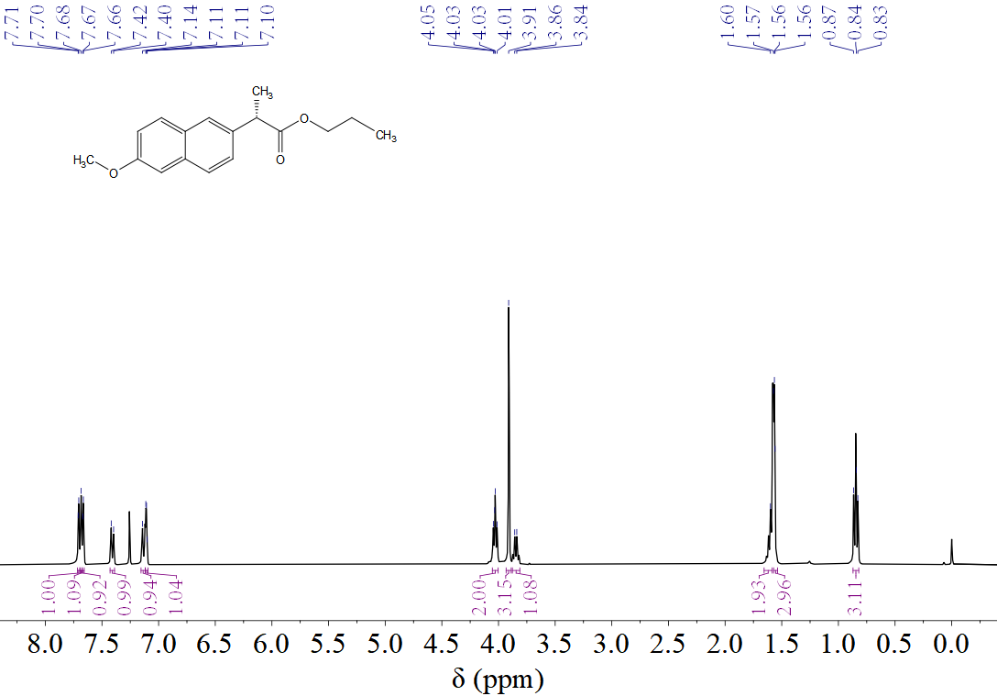


**Figure S31.** ^1^H NMR spectrum of S-NAP-PE in CDCl_3_ at room temperature.


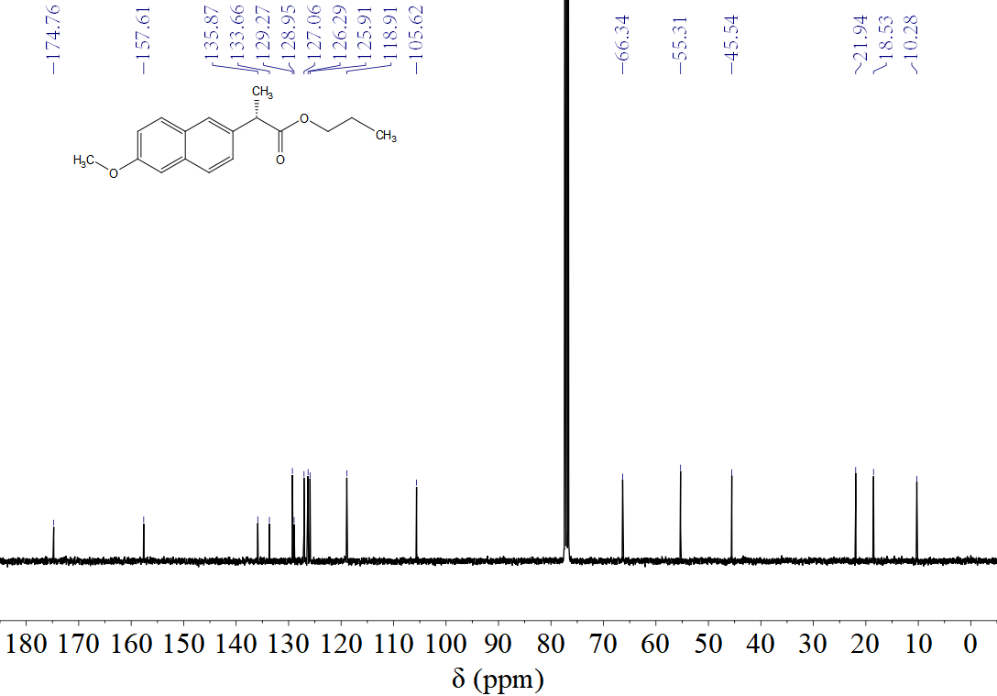


**Figure S32.** ^13^C NMR spectrum of S-NAP-PE in CDCl_3_ at room temperature.


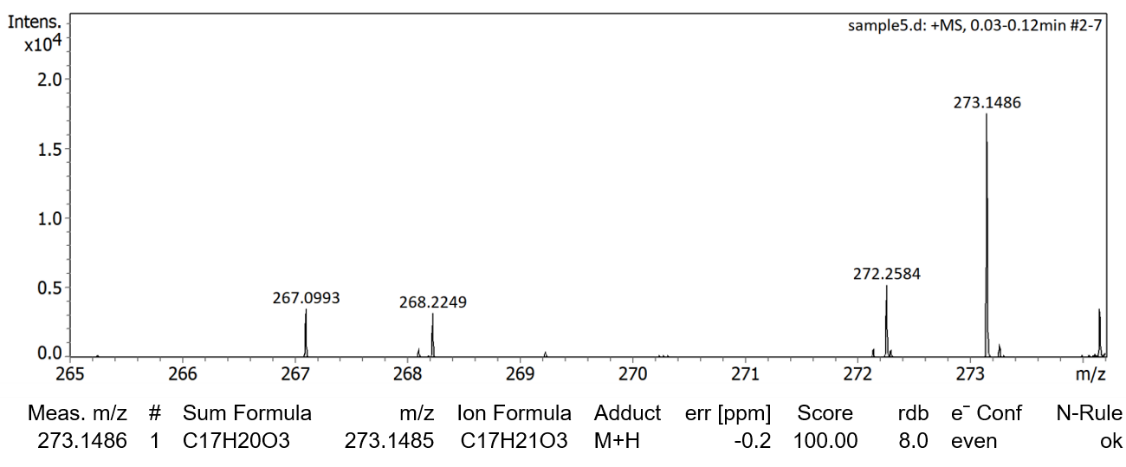


**Figure S33.** HRMS spectrum of S-NAP-PE.


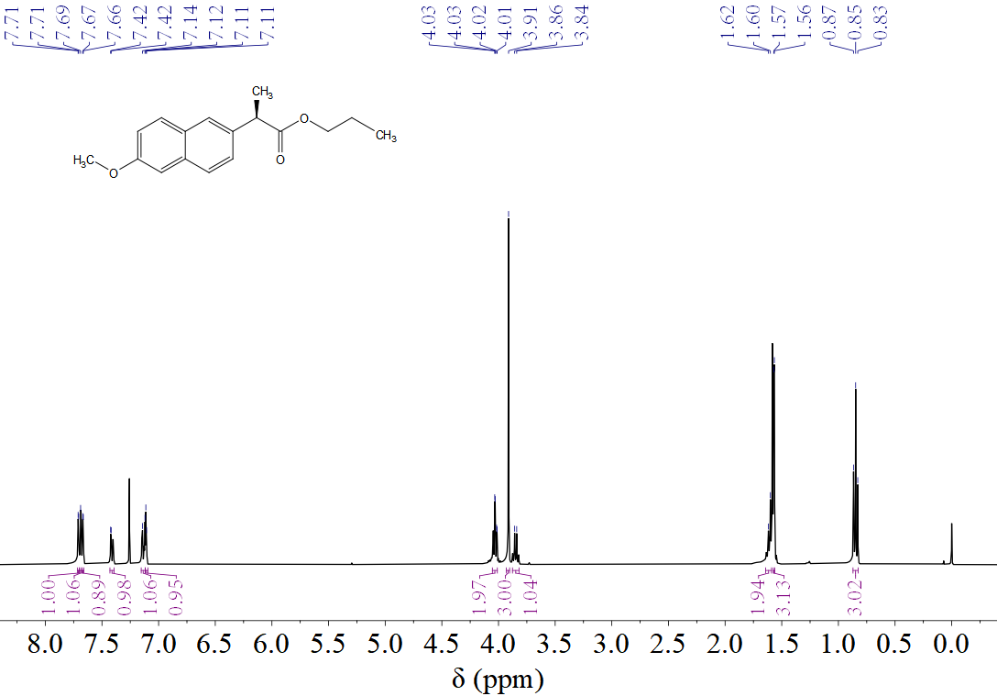


**Figure S34.** ^1^H NMR spectrum of R-NAP-PE in CDCl_3_ at room temperature.


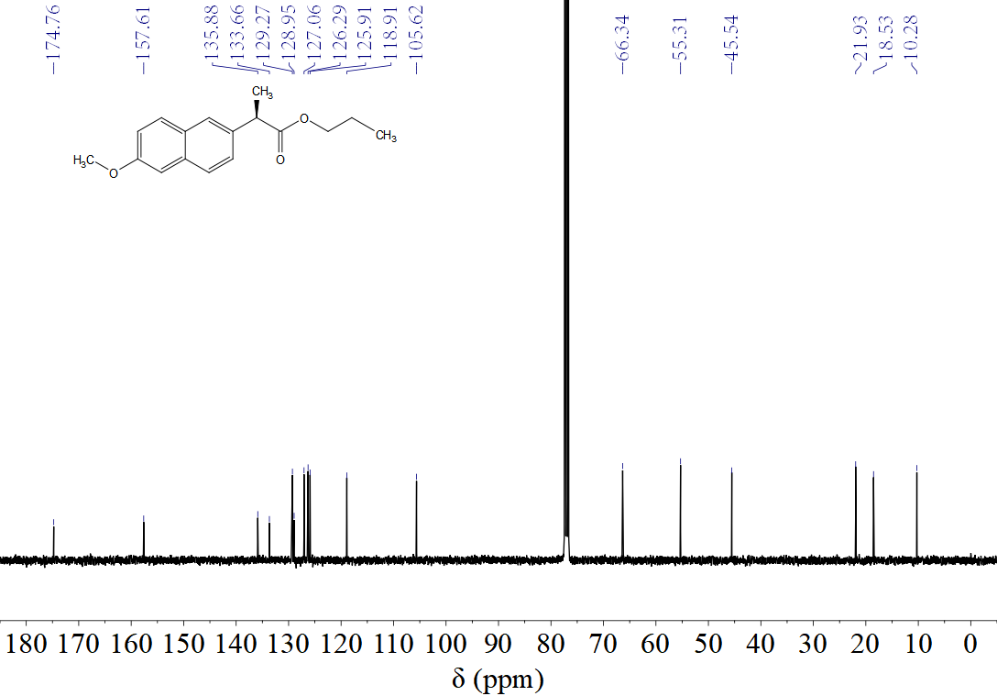


**Figure S35.** ^13^C NMR spectrum of R-NAP-PE in CDCl_3_ at room temperature.


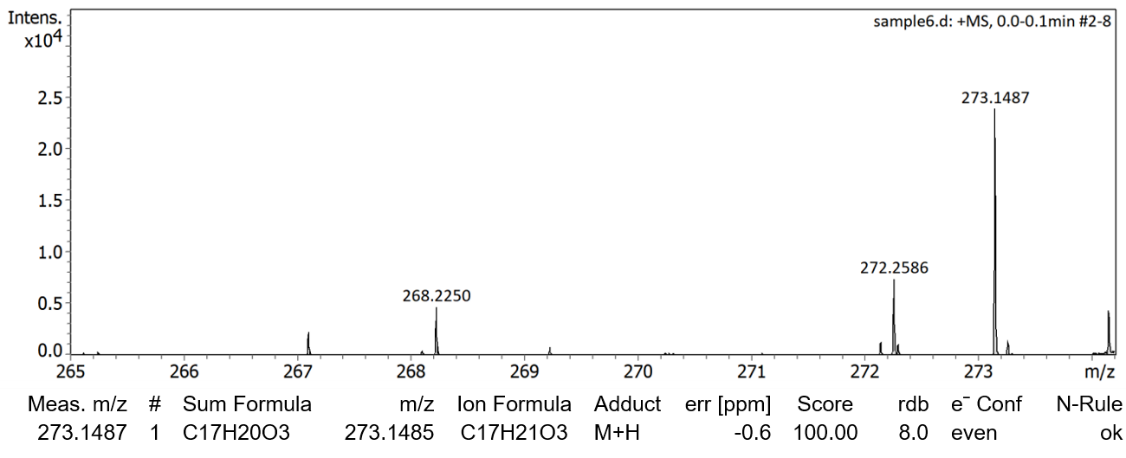


**Figure S36.** HRMS spectrum of R-NAP-PE.


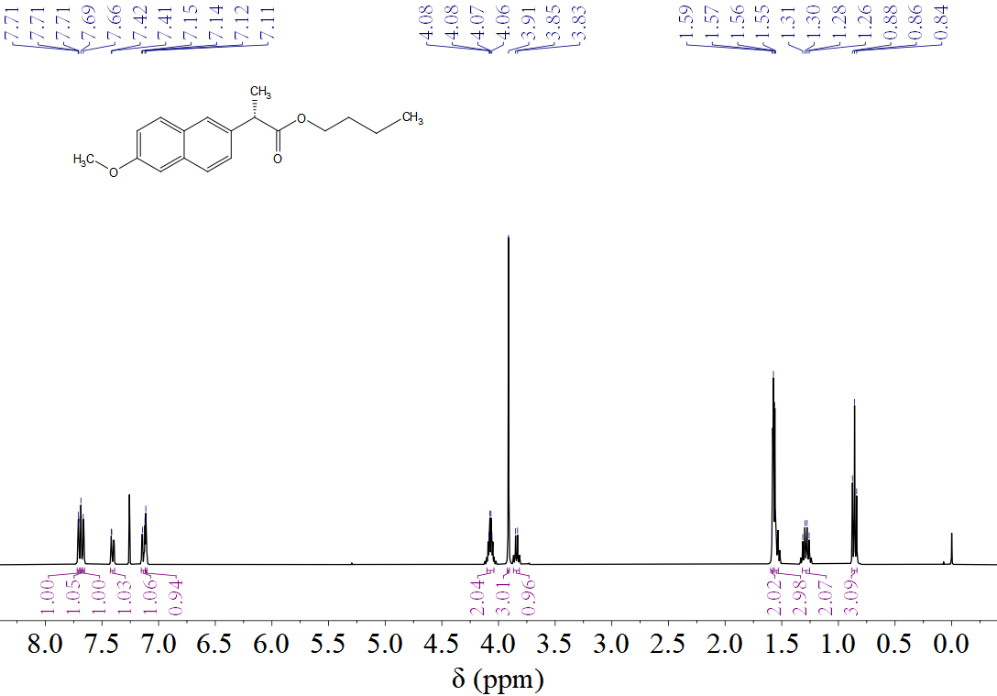


**Figure S37.** ^1^H NMR spectrum of S-NAP-BE in CDCl_3_ at room temperature.


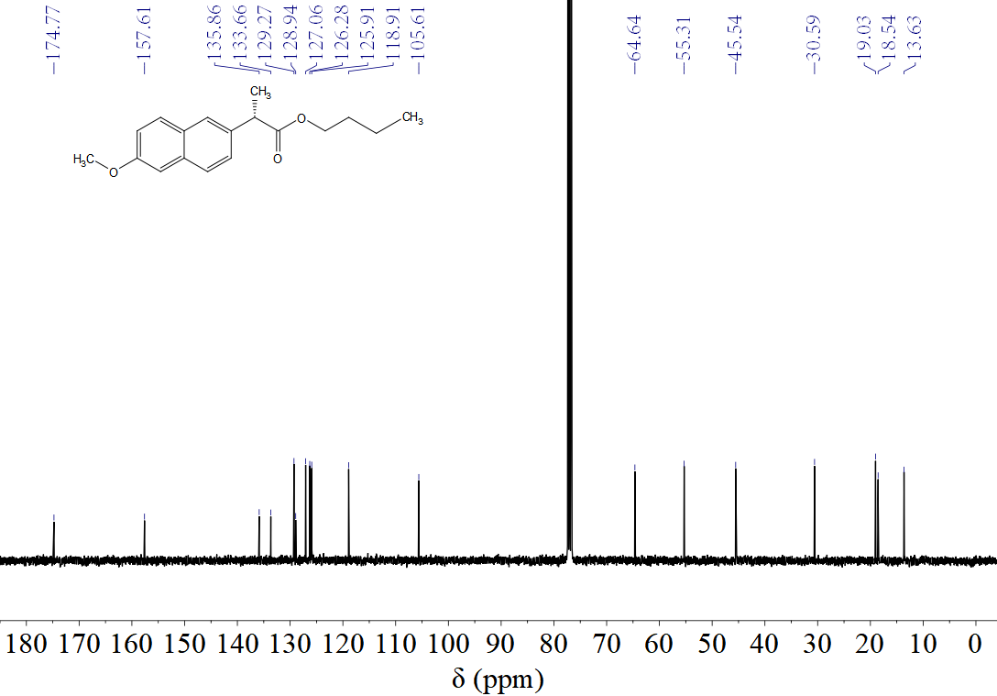


**Figure S38.** ^13^C NMR spectrum of S-NAP-BE in CDCl_3_ at room temperature.


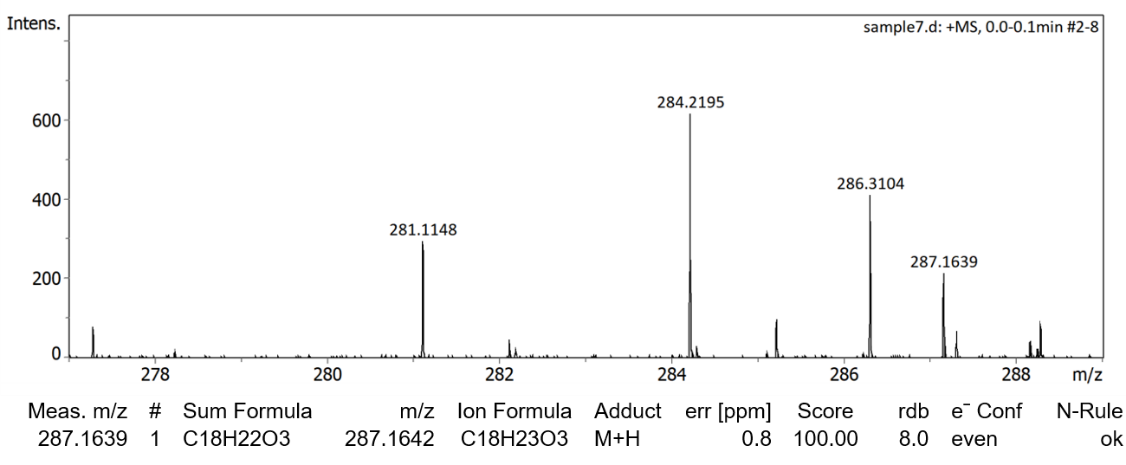


**Figure S39.** HRMS spectrum of S-NAP-BE.


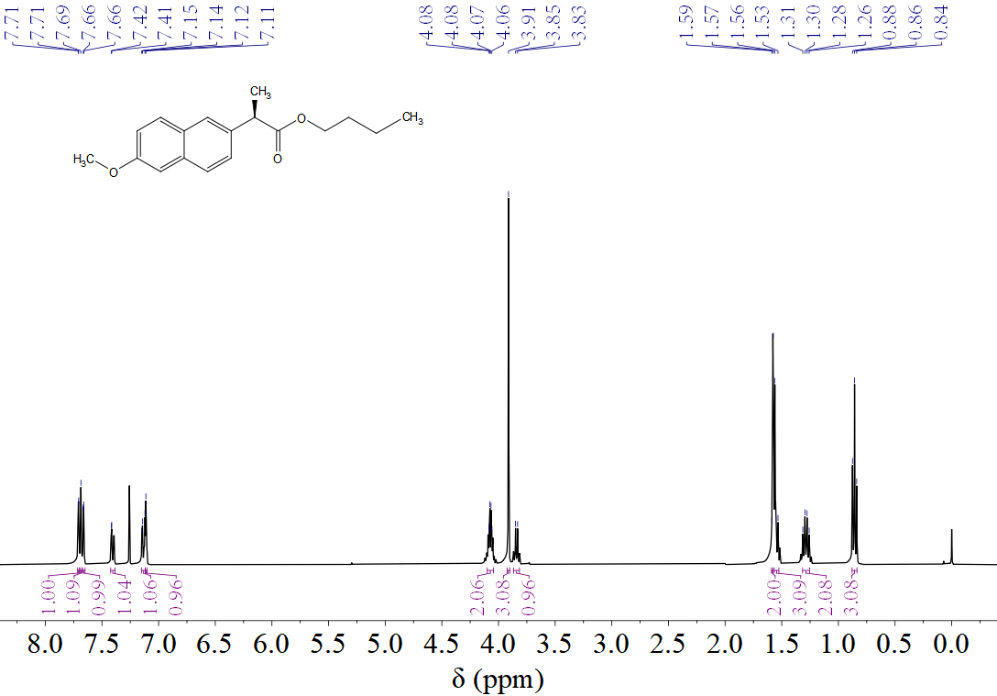


**Figure S40.** ^1^H NMR spectrum of R-NAP-BE in CDCl_3_ at room temperature.


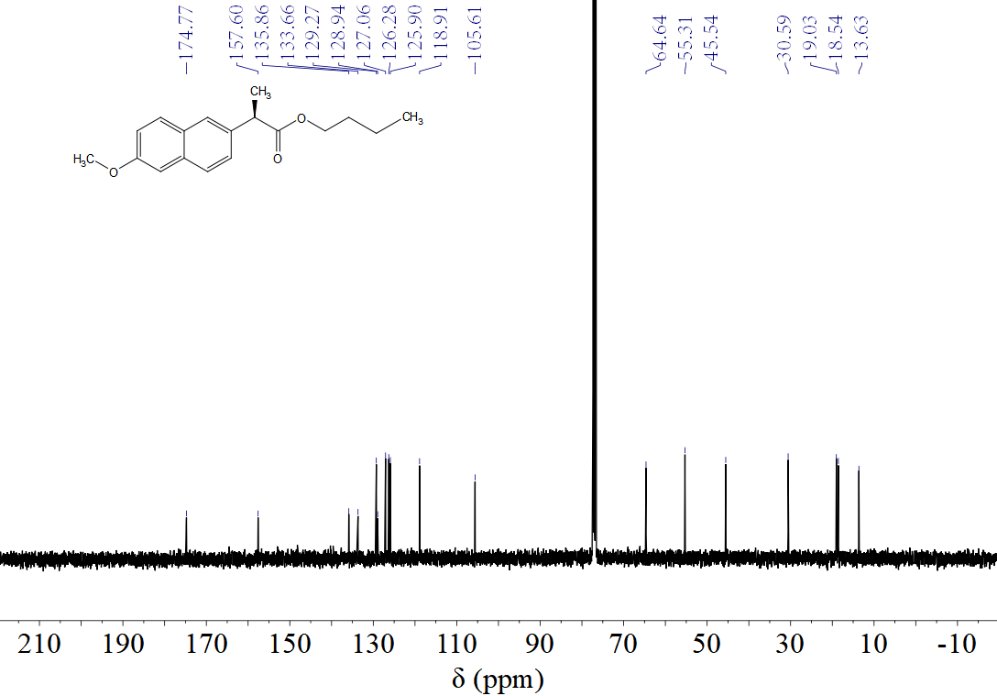


**Figure S41.** ^13^C NMR spectrum of R-NAP-BE in CDCl_3_ at room temperature.


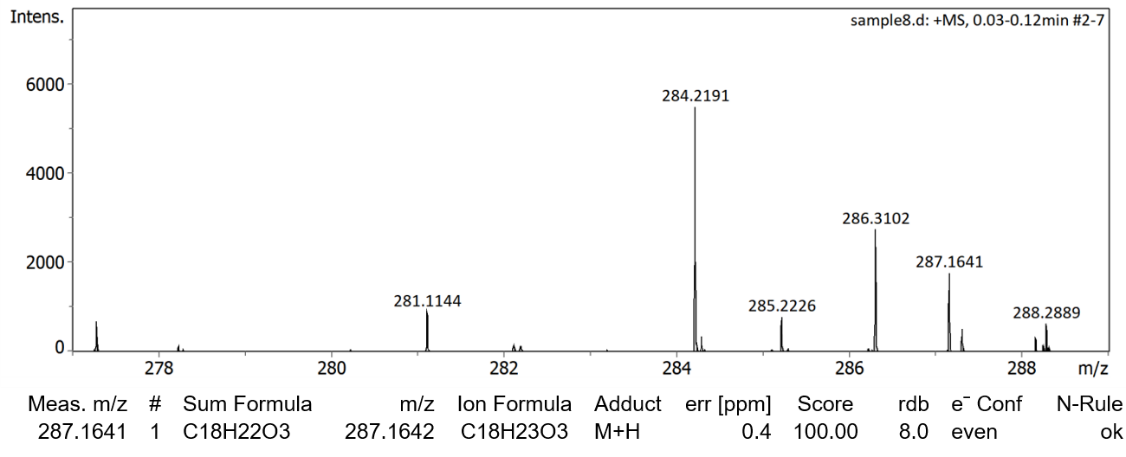


**Figure S42.** HRMS spectrum of R-NAP-BE.


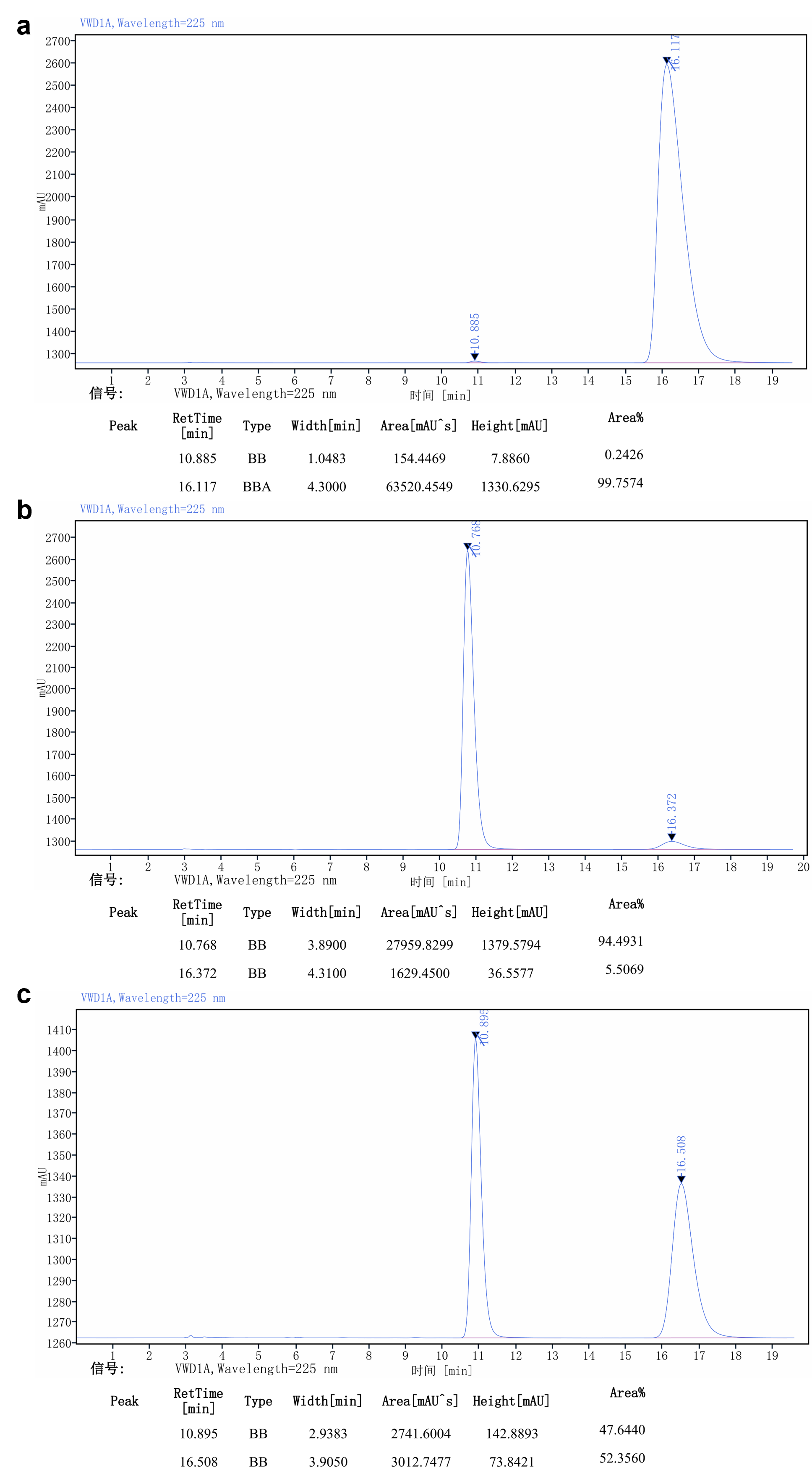


**Figure S43.** Chiral high performance liquid chromatogram (Chiral HPLC) spectrum of (a) S-NAP-ME, (b) R-NAP-ME and (c) rac-NAP-ME in 1% isopropanol in n-hexane monitored at the onset absorption of 225 nm.


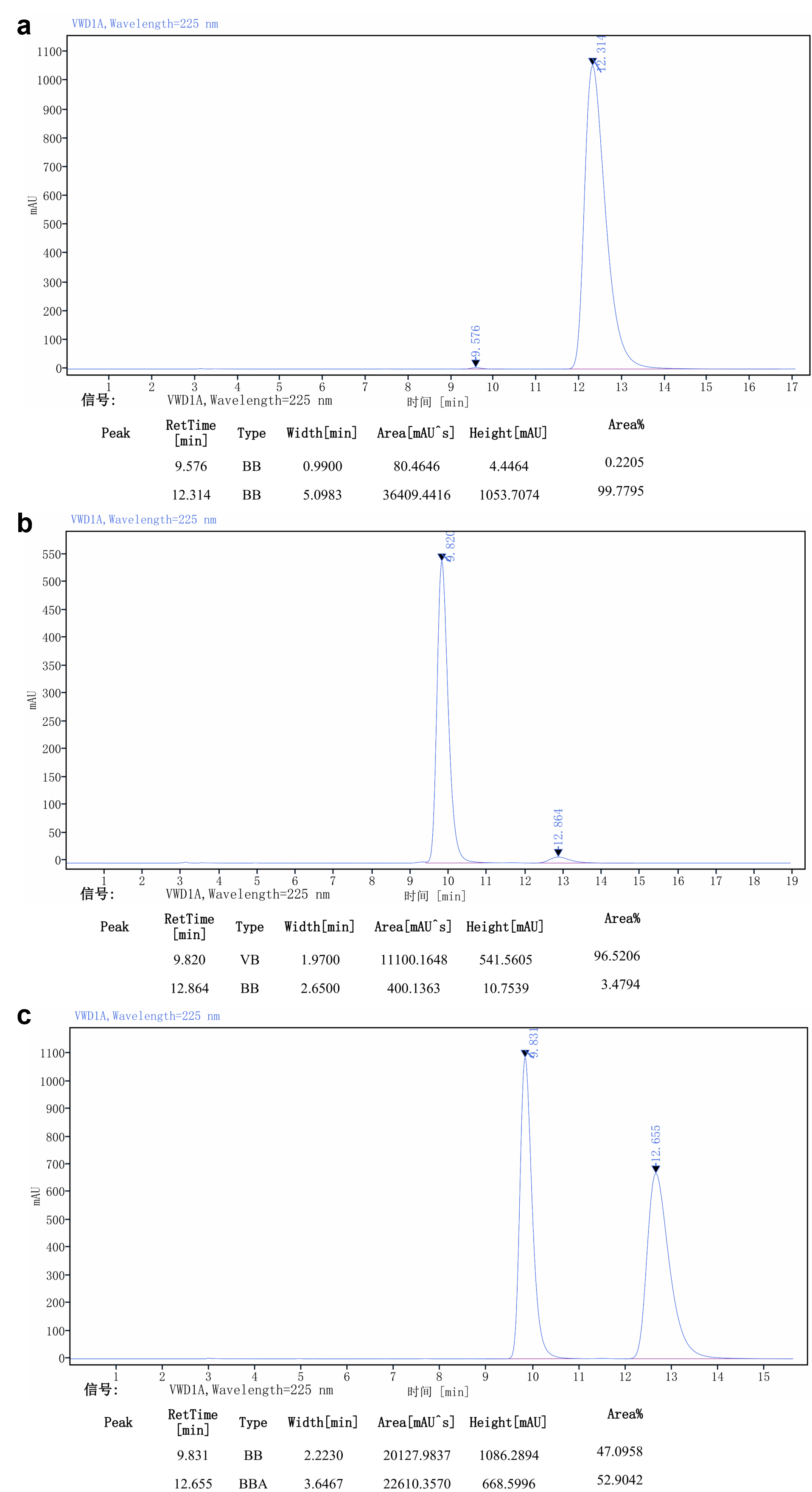


**Figure S44.** Chiral high performance liquid chromatogram (Chiral HPLC) spectrum of (a) S-NAP-EE, (b) R-NAP-EE and (c) rac-NAP-EE in 1% isopropanol in n-hexane monitored at the onset absorption of 225 nm.


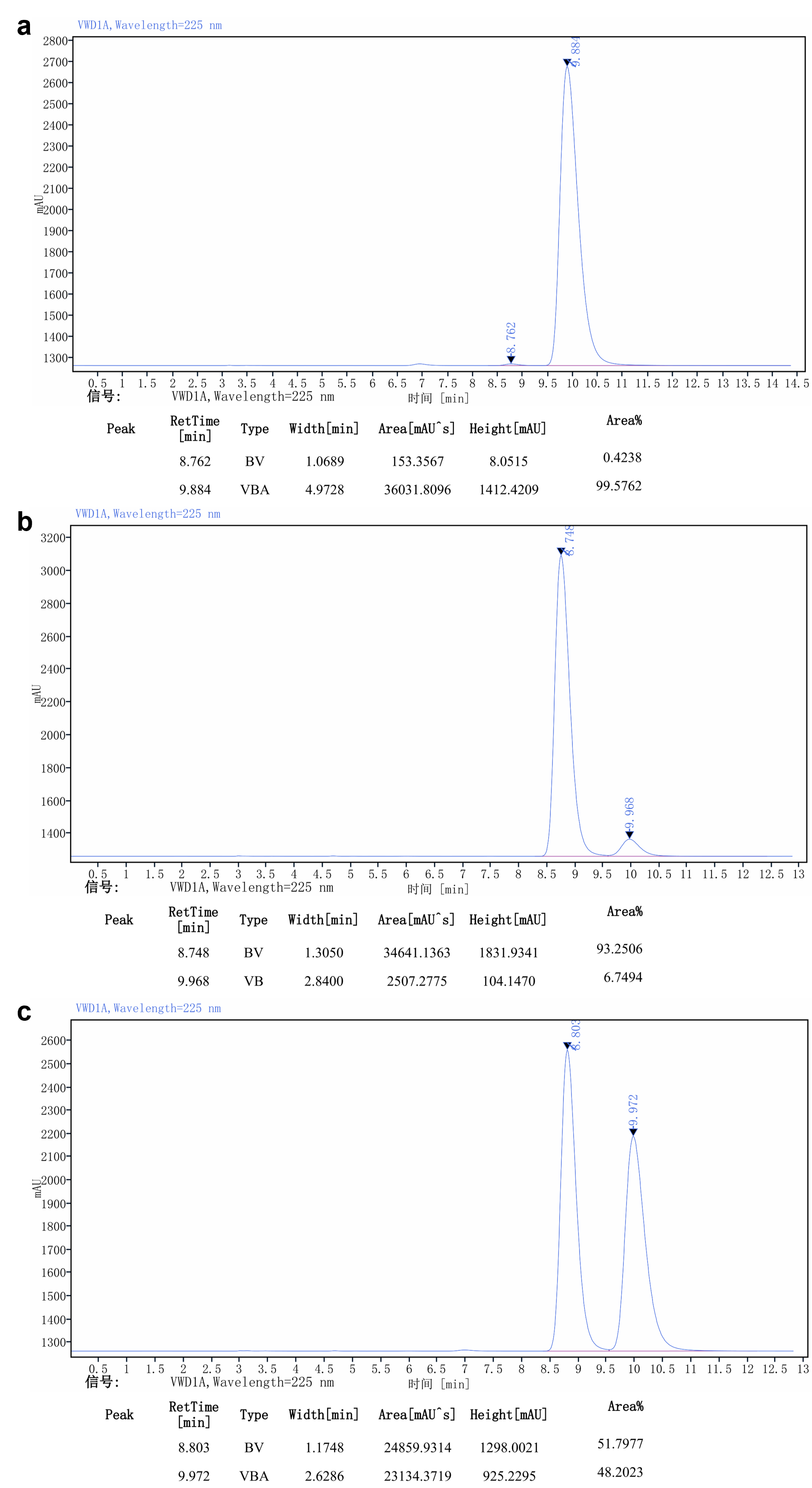


**Figure S45.** Chiral high performance liquid chromatogram (Chiral HPLC) spectrum of (a) S-NAP-PE, (b) R-NAP-PE and (c) rac-NAP-PE in 1% isopropanol in n-hexane monitored at the onset absorption of 225 nm.


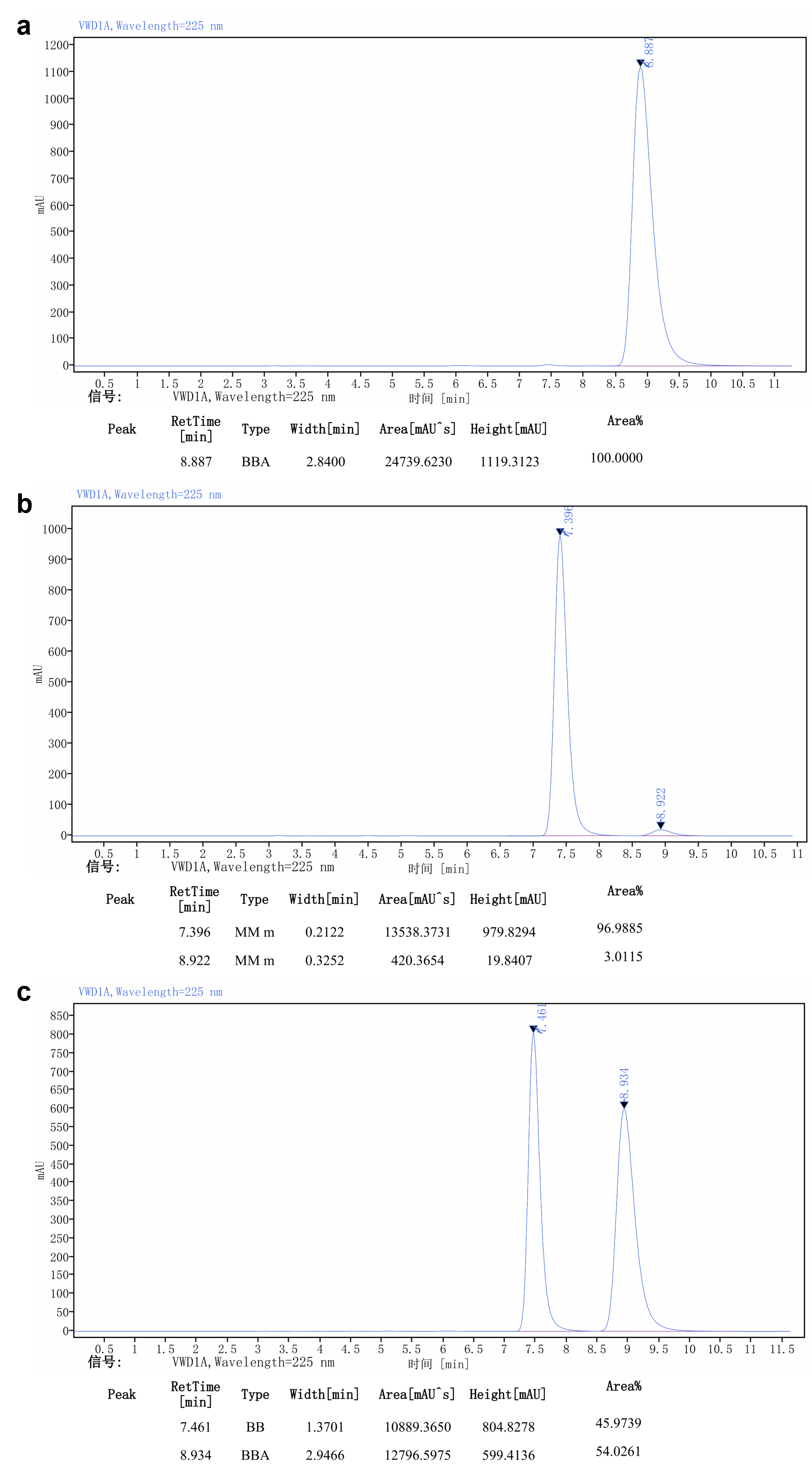


**Figure S46.** Chiral high performance liquid chromatogram (Chiral HPLC) spectrum of (a) S-NAP-BE, (b) R-NAP-BE and (c) rac-NAP-BE in 1% isopropanol in n-hexane monitored at the onset absorption of 225 nm.


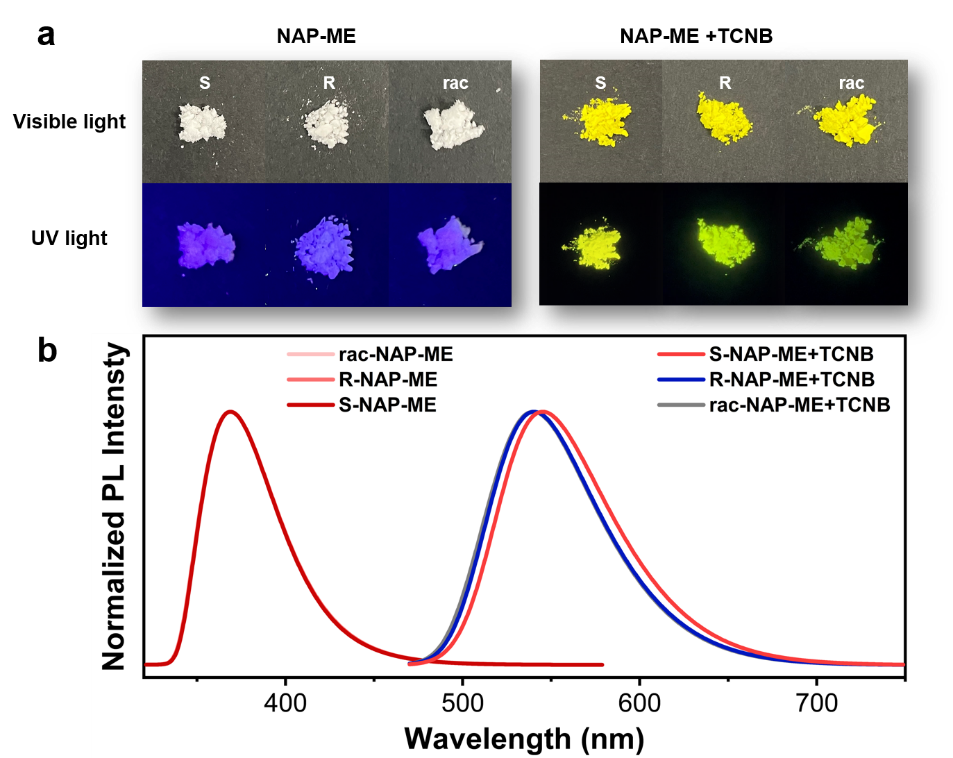


**Figure S47.** (a) Photographs of S-NAP-ME, R-NAP-ME and rac-NAP-ME before and after interaction with TCNB under visible light and UV light (365 nm). (b) Normalized PL spectra of S-NAP-ME, R-NAP-ME and rac-NAP-ME before and after interaction with TCNB.


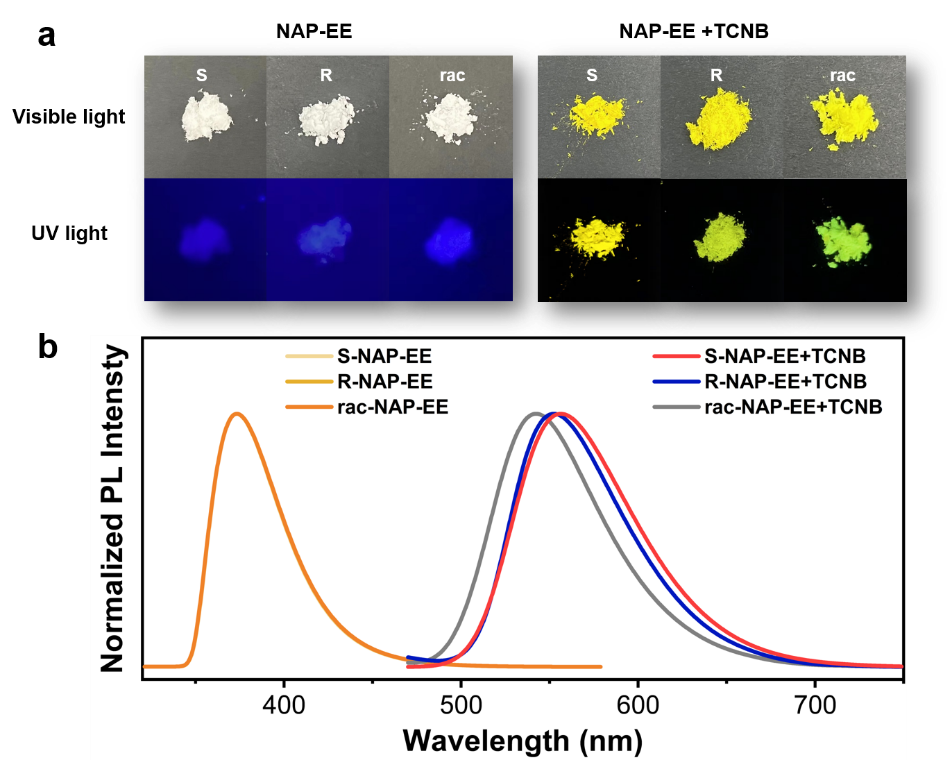


**Figure S48.** (a) Photographs of S-NAP-EE, R-NAP-EE and rac-NAP-EE before and after interaction with TCNB under visible light and UV light (365 nm). (b) Normalized PL spectra of S-NAP-EE, R-NAP-EE and rac-NAP-EE before and after interaction with TCNB.


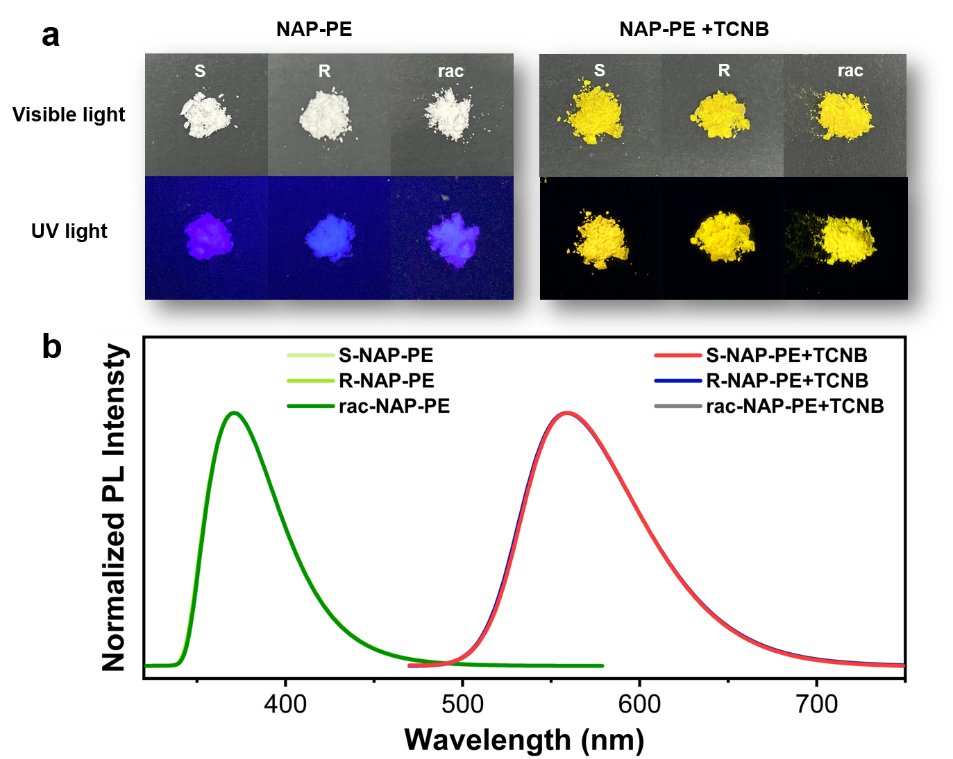


**Figure S49.** (a) Photographs of S-NAP-PE, R-NAP-PE and rac-NAP-PE before and after interaction with TCNB under visible light and UV light (365 nm). (b) Normalized PL spectra of S-NAP-PE, R-NAP-PE and rac-NAP-PE before and after interaction with TCNB.


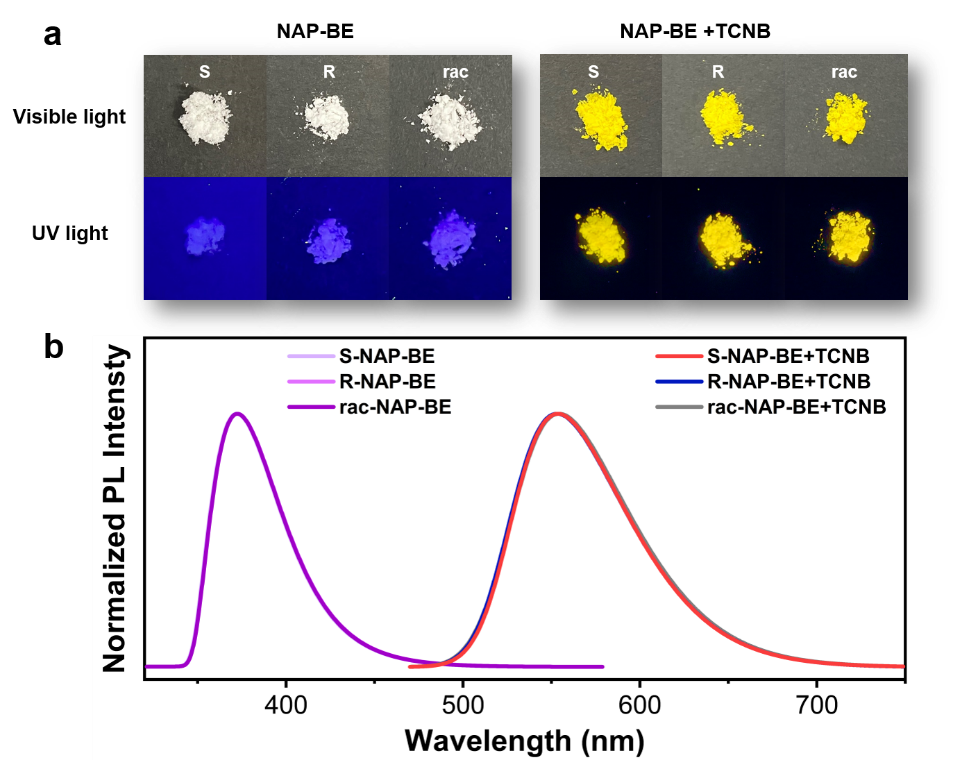


**Figure S50.** (a) Photographs of S-NAP-BE, R-NAP-BE and rac-NAP-BE before and after interaction with TCNB under visible light and UV light (365 nm). (b) Normalized PL spectra of S-NAP-BE, R-NAP-BE and rac-NAP-BE before and after interaction with TCNB.

| **Table S8. Fluorescence wavelengths of the synthesized chiral compounds before and after interaction with TCNB.** | | | | |
| --- | --- | --- | --- | --- |
| **Samples** | **PL _em_/nm ^[a]^** | **Samples** | **PL _em_/nm ^[b]^** |  |
| **S-NAP-ME** | 369 | S-NAP-ME+TCNB | 545 |  |
| **R-NAP-ME** | 369 | R-NAP-ME+TCNB | 540 |  |
| **rac-NAP-ME** | 369 | rac-NAP-ME+TCNB | 540 |  |
| **S-NAP-EE** | 373 | S-NAP-EE+TCNB | 556 |  |
| **R-NAP-EE** | 373 | R-NAP-EE+TCNB | 552 |  |
| **rac-NAP-EE** | 373 | rac-NAP-EE+TCNB | 542 |  |
| **S-NAP-PE** | 371 | S-NAP-PE+TCNB | 559 |  |
| **R-NAP-PE** | 371 | R-NAP-PE+TCNB | 559 |  |
| **rac-NAP-PE** | 371 | rac-NAP-PE+TCNB | 559 |  |
| **S-NAP-BE** | 372 | S-NAP-BE+TCNB | 553 |  |
| **R-NAP-BE** | 372 | R-NAP-BE+TCNB | 553 |  |
| **rac-NAP-BE** | 372 | rac-NAP-BE+TCNB | 553 |  |

[a] Fluorescence emission wavelength (em) under 300 nm UV excitation. [b] Fluorescence emission wavelength (em) under 400 nm UV excitation.

| **Table S9. The single crystal X-ray crystallographic data of S-NAP-PE+TCNB, R-NAP-PE+TCNB and rac-NAP-PE+TCNB.** | | | | |
| --- | --- | --- | --- | --- |
| **Compound** | **S-NAP-PE+TCNB** | **R-NAP-PE+TCNB** | **rac-NAP-PE-TCNB** |  |
| **Empirical formula** | C_27_H_22_N_4_O_3_ | C_27_H_22_N_4_O_3_ | C_27_H_22_N_4_O_3_ |  |
| **Formula weight** | 450.48 | 450.48 | 450.48 |  |
| **Temperature/K** | 149.99(10) | 99.96(18) | 99.97(19) |  |
| **Crystal system** | monoclinic | monoclinic | monoclinic |  |
| **Space group** | P2_1_ | P2_1_ | P2_1_/c |  |
| **a/Å** | 6.98728(5) | 6.9630(2) | 19.6770(5) |  |
| **b/Å** | 17.00175(14) | 16.9563(5) | 16.9602(4) |  |
| **c/Å** | 19.67581(17) | 19.6315(7) | 6.9740(2) |  |
| **α/°** | 90 | 90 | 90 |  |
| **β/°** | 98.3070(8) | 98.180(3) | 98.687(2) |  |
| **γ/°** | 90 | 90 | 90 |  |
| **Volume/Å^3^** | 2312.88(3) | 2294.24(13) | 2300.70(10) |  |
| **Z** | 4 | 4 | 4 |  |
| **ρcalcg/cm^3^** | 1.294 | 1.304 | 1.301 |  |
| **μ/mm^‑1^** | 0.700 | 0.705 | 0.703 |  |
| **F(000)** | 944.0 | 944.0 | 944.0 |  |
| **Crystal size/mm^3^** | 0.15 × 0.12 × 0.11 | 0.14 × 0.12 × 0.1 | 0.16 × 0.12 × 0.1 |  |
| **Radiation** | Cu Kα (λ = 1.54184) | Cu Kα (λ = 1.54184) | Cu Kα (λ = 1.54184) |  |
| **2θ range for data collection/°** | 4.538 to 153.164 | 6.918 to 148.748 | 6.914 to 148.53 |  |
| **Index ranges** | -4 ≤ h ≤ 8, -21 ≤ k ≤ 21, -24 ≤ l ≤ 24 | -8 ≤ h ≤ 4, -21 ≤ k ≤ 20, -24 ≤ l ≤ 24 | -24 ≤ h ≤ 24, -14 ≤ k ≤ 21, -8 ≤ l ≤ 5 |  |
| **Reflections collected** | 21225 | 12761 | 12241 |  |
| **Independent reflections** | 8521 [R_int_ = 0.0190,  R_sigma_ = 0.0151] | 7590 [R_int_ = 0.0276,  R_sigma_ = 0.0400] | 4548 [R_int_ = 0.0411,  R_sigma_ = 0.0472] |  |
| **Data/restraints/parameters** | 8521/1/620 | 7590/1/619 | 4548/0/330 |  |
| **Goodness-of-fit on F^2^** | 1.039 | 1.068 | 1.027 |  |
| **Final R indexes [I>=2σ (I)]** | R_1_ = 0.0280, wR_2_ = 0.0775 | R_1_ = 0.0453, wR_2_ = 0.1240 | R_1_ = 0.0497, wR_2_ = 0.1249 |  |
| **Final R indexes [all data]** | R_1_ = 0.0282, wR_2_ = 0.0776 | R_1_ = 0.0482, wR_2_ = 0.1257 | R_1_ = 0.0592, wR_2_ = 0.1303 |  |
| **Largest diff. peak/hole / e Å^-3^** | 0.26/-0.14 | 0.57/-0.25 | 0.38/-0.27 |  |
| **CCDC Number** | 2325365 | 2325366 | 2325367 |  |


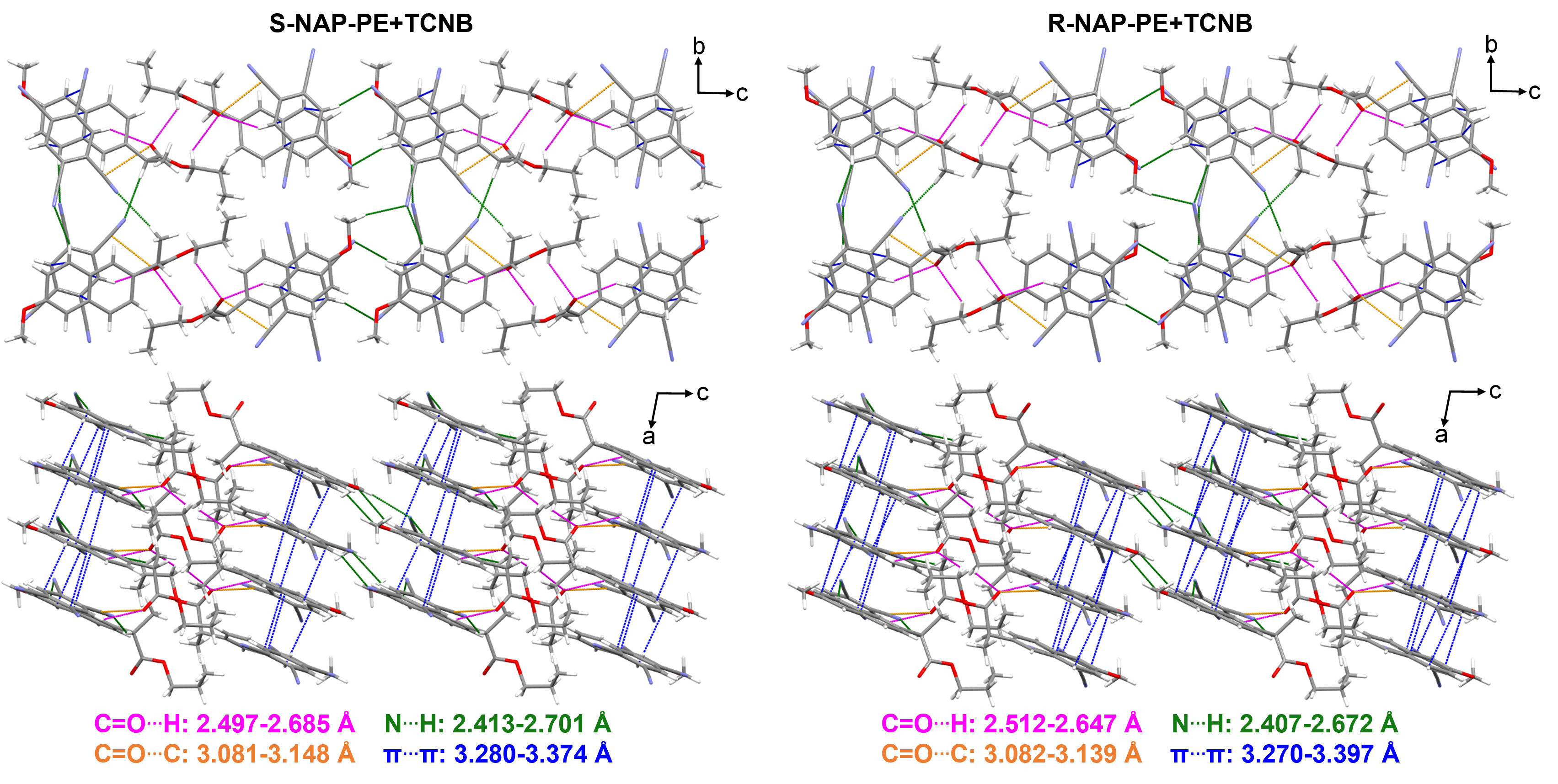


**Figure S51.** Molecular packing and intermolecular interactions of S-NAP-PE+TCNB and R-NAP-PE+TCNB.

**Table S10.** Calculated minimum energy and stabilization energy of S-NAP-PE+TCNB, R-NAP-PE+TCNB and rac-NAP-PE+TCNB from single crystal.

|  | **Minimum energy (eV)** | **Stabilization energy (eV)** |
| --- | --- | --- |
| **S-NAP-PE in S-NAP-PE+TCNB** | -23815.692 |  |
| **TCNB in S-NAP-PE+TCNB** | -16079.559 |  |
| **S-NAP-PE+TCNB** | -40356.136 | -460.885 |
| **S-NAP-PE in rac-NAP-PE+TCNB** | -23813.417 |  |
| **TCNB in S-NAP-PE+TCNB of rac-NAP-PE+TCNB** | -16077.548 |  |
| **S-NAP-PE+TCNB in rac-NAP-PE+TCNB** | -40356.166 | -465.201 |
| **R-NAP-PE in R-NAP-PE+TCNB** | -23815.899 |  |
| **TCNB in R-NAP-PE+TCNB** | -16079.899 |  |
| **R-NAP-PE+TCNB** | -40356.221 | -460.422 |
| **R-NAP-PE in rac-NAP-PE+TCNB** | -23815.844 |  |
| **TCNB in R-NAP-PE+TCNB of rac-NAP-PE+TCNB** | -16079.845 |  |
| **R-NAP-PE+TCNB in rac-NAP-PE+TCNB** | -40356.212 | -460.523 |


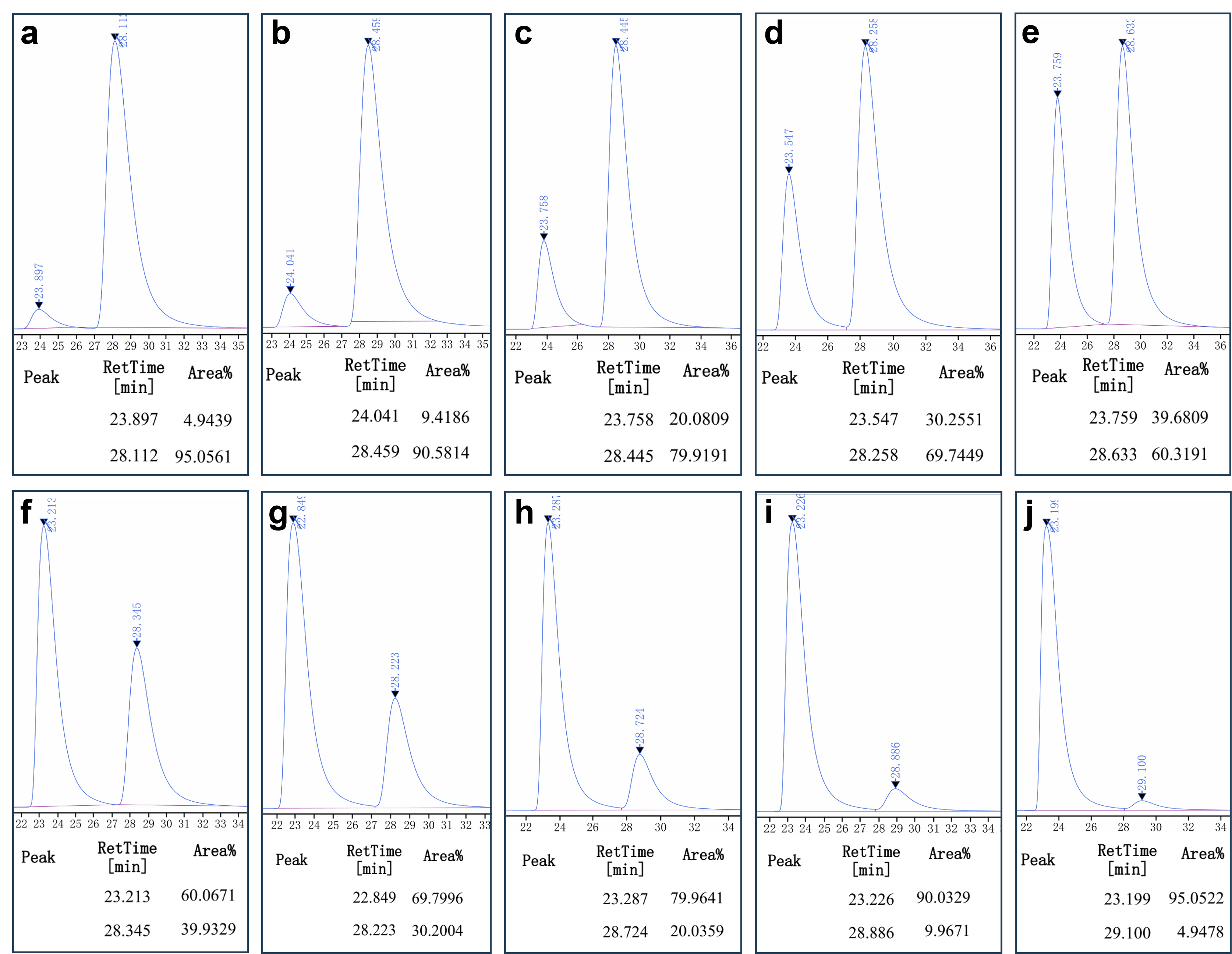


**Figure S52.** Chiral high performance liquid chromatogram (Chiral HPLC) spectra of naproxen before visualized chiral purification. (a-e correspond to 1-5 and f-j correspond to 7-11 in Table 1)


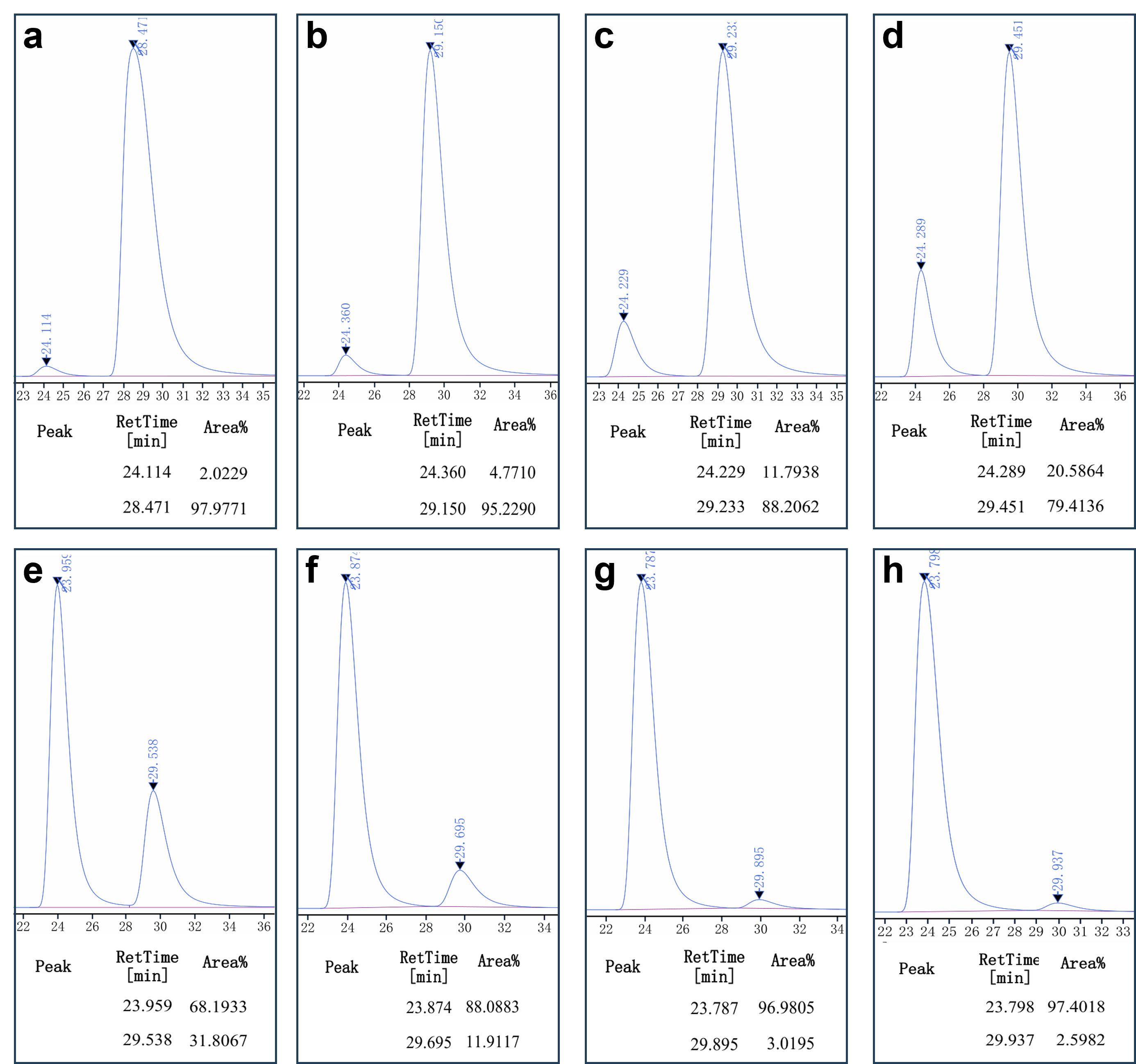


**Figure S53.** Chiral high performance liquid chromatogram (Chiral HPLC) spectra of naproxen after visualized chiral purification. (Group 1, a-d correspond to 1-4 and e-h correspond to 8-11 in Table 1)


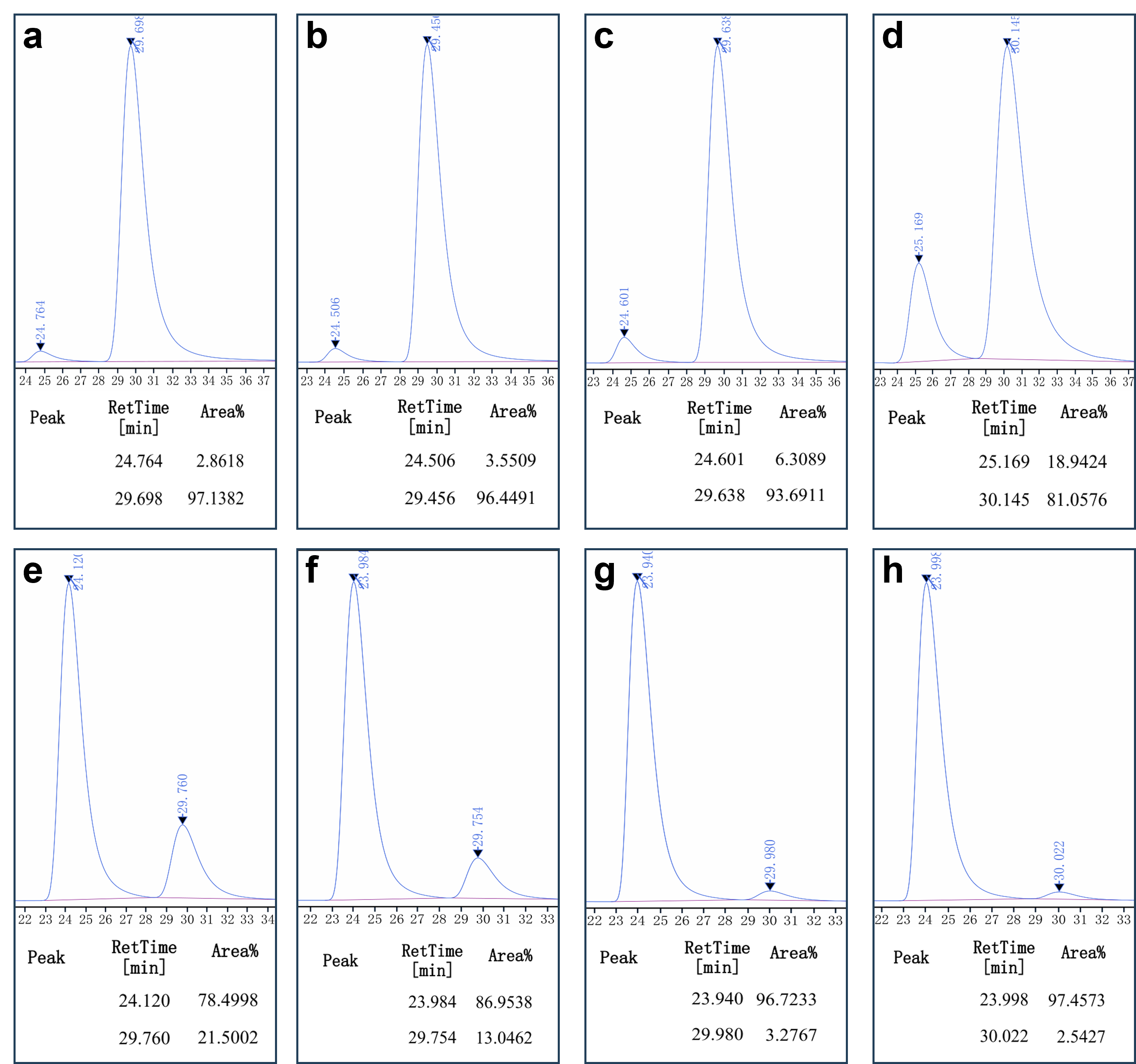


**Figure S54.** Chiral high performance liquid chromatogram (Chiral HPLC) spectra of naproxen after visualized chiral purification. (Group 2, a-d correspond to 1-4 and e-h correspond to 8-11 in Table 1)


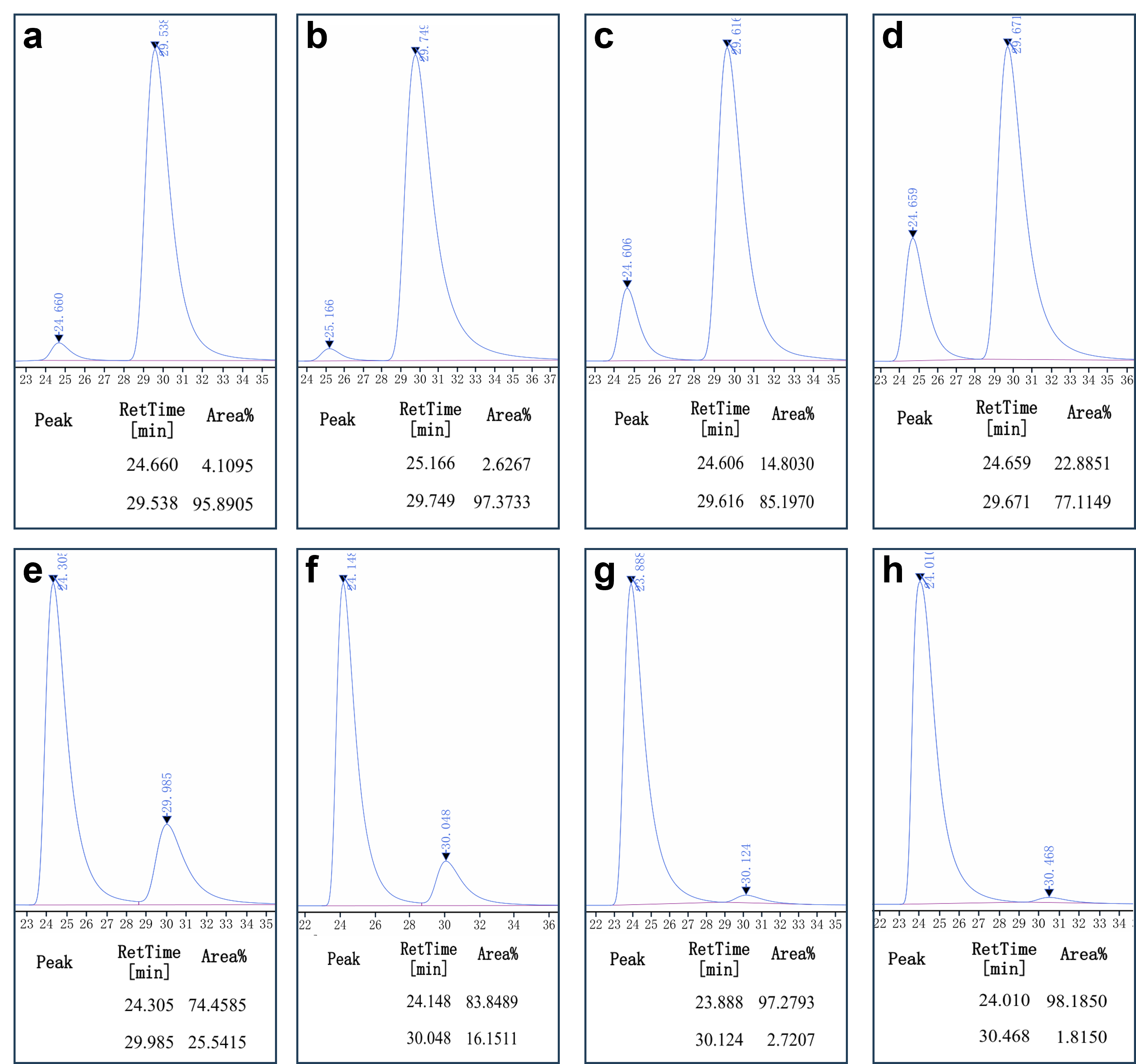


**Figure S55.** Chiral high performance liquid chromatogram (Chiral HPLC) spectra of naproxen after visualized chiral purification. (Group 3, a-d correspond to 1-4 and e-h correspond to 8-11 in Table 1)

References

[1] M. J. Frisch, G. W. Trucks, H. B. Schlegel, G. E. Scuseria, M. A. Robb, J. R. Cheeseman, G. Scalmani, V. Barone, G. A. Petersson, H. Nakatsuji, X. Li, M. Caricato, A. V. Marenich, J. Bloino, B. G. Janesko, R. Gomperts, B. Mennucci, H. P. Hratchian, J. V. Ortiz, A. F. Izmaylov, J. L. Sonnenberg, D. WilliamsYoung, F. Ding, F. Lipparini, F. Egidi, J. Goings, B. Peng, A. Petrone, T. Henderson, D. Ranasinghe, V. G. Zakrzewski, J. Gao, N. Rega, G. Zheng, W. Liang, M. Hada, M. Ehara, K. Toyota, R. Fukuda, J. Hasegawa, M. Ishida, T. Nakajima, Y. Honda, O. Kitao, H. Nakai, T. Vreven, K. Throssell, J. A. Montgomery, Jr., J. E. Peralta, F. Ogliaro, M. J. Bearpark, J. J. Heyd, E. N. Brothers, K. N. Kudin, V. N. Staroverov, T. A. Keith, R. Kobayashi, J. Normand, K. Raghavachari, A. P. Rendell, J. C. Burant, S. S. Iyengar, J. Tomasi, M. Cossi, J. M. Millam, M. Klene, C. Adamo, R. Cammi, J. W. Ochterski, R. L. Martin, K. Morokuma, O. Farkas, J. B. Foresman, D. J. Fox, Gaussian 16, Revision A.03, Gaussian, Inc., Wallingford CT, **2016**.

[2] Y. Zhao, D. G. Truhlar, *Theor. Chem. Acc.* **2008,** *120*, 215.

[3] L. W. Chung, W. M. C. Sameera, R. Ramozzi, A. J. Page, M. Hatanaka, G. P. Petrova, T. V. Harris, X. Li, Z. Ke, F. Liu, H. B. Li, L. Ding, K. Morokuma, *Chem. Rev.* **2015,** *115*, 5678.

[4] E. Runge, E. K. U. Gross, *Phys. Rev. Lett.* **1984,** *52*, 997.

[5] T. Lu, F. W. Chen, *J. Comput. Chem.* **2012,** *33*, 580.

[6] T. Lu, Q. Chen, *J. Comput. Chem.* **2022,** *43*, 539.

[7] C. Lefebvre, G. Rubez, H. Khartabil, J. C. Boisson, J. Contreras-García, E. Hénon, *Phys. Chem. Chem. Phys.* **2017,** *19*, 17928.

[8] W. Humphrey, A. Dalke, K. Schulten, *J. Mol. Graphics.* **1996,** *14*, 33.
